# Genome-wide association analyses of common infections in a large practice-based biobank

**DOI:** 10.1186/s12864-022-08888-9

**Published:** 2022-09-27

**Authors:** Lan Jiang, V. Eric Kerchberger, Christian Shaffer, Alyson L. Dickson, Michelle J. Ormseth, Laura L. Daniel, Barbara G. Carranza Leon, Nancy J. Cox, Cecilia P. Chung, Wei-Qi Wei, C. Michael Stein, QiPing Feng

**Affiliations:** 1 Division of Clinical Pharmacology, Department of Medicine, Vanderbilt University Medical Center, Nashville, TN USA; 2 Department of Biomedical Informatics, Vanderbilt University Medical Center, Nashville, TN USA; 3 Division of Rheumatology and Immunology, Department of Medicine, Vanderbilt University Medical Center, Nashville, TN USA; 4 Division of Research and Development, Tennessee Valley Healthcare System, U.S. Department of Veterans Affairs, Nashville, TN USA; 5 Division of Diabetes, Endocrinology and Metabolism, Department of Medicine, Vanderbilt University Medical Center, Nashville, TN USA; 6 Department of Medicine, Vanderbilt Genetic Institute, Vanderbilt University Medical Center, Nashville, TN USA; 7 Department of Pharmacology, Vanderbilt University, Nashville, TN USA

**Keywords:** Infection, GWAS, EHR

## Abstract

**Introduction:**

Infectious diseases are common causes of morbidity and mortality worldwide. Susceptibility to infection is highly heritable; however, little has been done to identify the genetic determinants underlying common infectious diseases. One GWAS was performed using 23andMe information about self-reported infections; we set out to confirm previous loci and identify new ones using medically diagnosed infections.

**Methods:**

We used the electronic health record (EHR)-based biobank at Vanderbilt and diagnosis codes to identify cases of 12 infectious diseases in white patients: urinary tract infection, pneumonia, chronic sinus infections, otitis media, candidiasis, streptococcal pharyngitis, herpes zoster, herpes labialis, hepatitis B, infectious mononucleosis, tuberculosis (TB) or a positive TB test, and hepatitis C. We selected controls from patients with no diagnosis code for the candidate disease and matched by year of birth, sex, and calendar year at first and last EHR visits. We conducted GWAS using SAIGE and transcriptome-wide analysis (TWAS) using S-PrediXcan. We also conducted phenome-wide association study to understand associations between identified genetic variants and clinical phenotypes.

**Results:**

We replicated three 23andMe loci (*p* ≤ 0.05): herpes zoster and rs7047299-A (*p* = 2.6 × 10^–3^) and rs2808290-C (*p* = 9.6 × 10^–3^;); otitis media and rs114947103-C (*p* = 0.04). We also identified 2 novel regions (p ≤ 5 × 10^–8^): rs113235453-G for otitis media (*p* = 3.04 × 10^–8^), and rs10422015-T for candidiasis (*p* = 3.11 × 10^–8^). In TWAS, four gene-disease associations were significant: *SLC30A9* for otitis media (*p* = 8.06 × 10^–7^); *LRP3* and *WDR88* for candidiasis (*p* = 3.91 × 10^–7^ and *p* = 1.95 × 10^–6^); and *AAMDC* for hepatitis B (*p* = 1.51 × 10^–6^).

**Conclusion:**

We conducted GWAS and TWAS for 12 infectious diseases and identified novel genetic contributors to the susceptibility of infectious diseases.

**Supplementary Information:**

The online version contains supplementary material available at 10.1186/s12864-022-08888-9.

## Introduction

Infections are among the most common causes of morbidity and mortality worldwide, resulting in millions of deaths [1, 2]. Complications of serious infection in the U.S. contribute to 1 in 3 hospital deaths and ~ 250,000 deaths annually [3]. Susceptibility to infection is highly heritable, likely due to major selection pressure over millennia, when infection was the leading cause of death and no effective antimicrobials existed [4]. More than 300 rare Mendelian disorders resulting from mutations predominantly in genes regulating immune response predispose individuals to infection [4, 5] and provide extreme proof of the critical importance of host genetic variation in susceptibility to infection. However, such variants do not account for the high heritability of susceptibility to infection seen in other studies. In a landmark twin study, adults who had been adopted as children had a 5.8-fold increased risk of dying from infection if one of their biological parents had died from infection before the age of 50 years [6]. Other twin studies have shown high heritability for traits such as infection (h^2^ = 0.43) [7], staphylococcal infection (h^2^ = 0.7) [8], and death due to infection (h^2^ = 0.4) [9].

Despite high heritability, the genetics of susceptibility to infection is poorly defined and is recognized as a neglected area of research: only 4% of the catalog of genome wide association studies (GWAS) relates to the broad area of infectious disease [10]. Many attempts to identify the genetic determinants underlying common infections have major limitations. First, associations have been sought in small candidate gene studies; second, few GWAS studies have been broadly relevant to patients in the U.S. One of the largest GWAS was performed using 23andMe data with self-reported health history for 23 infections [11]. In that study, Tian et al. identified genes that play key roles in immune response and inflammatory processes associated with susceptibility to infections. However, the identified associations have not been tested in a real-world setting with infections diagnosed by physicians, and relatively few loci have been identified.

The COVID-19 pandemic resulted in urgent work to expand our understanding of the genetic mechanisms underlying severe respiratory viral infection and its complications. A recent meta-analysis of 46 independent GWASs identified loci that contribute to susceptibility or severity of COVID-19 infection [12] — supporting the critical role of host genetics in infectious diseases. However, whether the identified COVID-19 loci are also involved in susceptibility to other respiratory infections is unclear.

Biobanks linked to patients’ electronic health records (EHRs) provide an unprecedented opportunity to perform genetic studies and understand infectious disease. The biobank at Vanderbilt (BioVU) is one of the largest practice-based biobanks in the U.S. We set out to replicate the observations from the previous 23andMe GWAS and test the associations between the identified variants and clinical phenotypes using phenome-wide association studies (PheWAS) to identify additional associated infections as well as co-morbidities that could predispose to infection. One of our primary objectives was to replicate the earlier findings from a GWAS study that used self-reported history of various infections as the phenotypes of interest with those of a GWAS study that used the more objective outcomes of medically diagnosed infections. Then, we conducted GWAS and transcriptome-wide association study (TWAS) to further define the role of host genetics in common infections. Last, we tested if previously identified COVID-19 loci also associated with susceptibility to pneumonia in our BioVU cohort [12].

## Methods

### Data sources

Data were obtained from the Synthetic Derivative (SD) and BioVU at Vanderbilt University Medical Center (VUMC) that contains a de-identified copy of the EHR for every patient and has genome-wide genotyping available for > 100,000 patients [13–15]. The BioVU follows the declaration of Helsinki. The study followed the declaration of Helsinki. The study was exempted by Vanderbilt University Medical Center Institutional Review Board.

### Study cohort

We included individuals whose race was identified as white in the de-identified EHR and who had genome-wide genotyping available. We identified patients with the infectious diseases of interest using the International Classification of Disease Clinical Modification, Ninth Revision (ICD9CM) and Tenth Revision (ICD10CM) codes (Supplement Table 1).

**Table 1:** Demographic summary for 12 common infections

Phenotype	Status	N	Female	Male	Age (years, mean ± SD)	EHR length (years, mean ± SD)
**Streptococcal pharyngitis**	**Control**	9970	6290	3680	31.8 ± 20.8	14 ± 6.8
	**Case**	997	617	380	30.7 ± 21	13.9 ± 5.8
**Candidiasis**	**Control**	11,010	6911	4099	51.2 ± 22.6	13 ± 7.4
	**Case**	2202	1385	817	52.9 ± 21.6	13.1 ± 7.4
**Herpes zoster**	**Control**	7510	4669	2841	65.8 ± 15.8	15.4 ± 7.1
	**Case**	751	455	296	66.4 ± 15.7	15.3 ± 6.7
**Hepatitis B**	**Control**	2230	710	1520	57.3 ± 19.9	14.6 ± 7.4
	**Case**	223	68	155	56 ± 14.7	14 ± 7.6
**UTI**	**Control**	46,795	26,484	20,311	53.5 ± 21.1	10.8 ± 7.1
	**Case**	9359	6889	2470	59.3 ± 21.2	13.8 ± 7.2
**Pneumonia**	**Control**	31,925	15,125	16,800	58.5 ± 21.1	11.8 ± 7.4
	**Case**	6385	3071	3314	58.8 ± 21.9	11.9 ± 7.5
**Infectious mononucleosis**	**Control**	1160	740	420	28.8 ± 18.7	13.9 ± 7.2
	**Case**	116	70	46	28.3 ± 16.3	13.7 ± 6.5
**Tuberculosis or a positive TB test**	**Control**	1020	597	423	56.1 ± 19.1	14.3 ± 6.8
	**Case**	102	56	46	56 ± 16.9	14.2 ± 6.5
**Otitis media**	**Control**	12,170	6879	5291	35.3 ± 22.8	13.8 ± 7.1
	**Case**	2434	1299	1135	32.3 ± 25.6	13.3 ± 6.3
**Chronic sinus infection**	**Control**	12,860	7969	4891	58.4 ± 18.6	15.4 ± 6.7
	**Case**	2572	1577	995	58.4 ± 17.6	15.5 ± 6.6
**Hepatitis C**	**Control**	6300	2211	4089	54.9 ± 20.4	12.3 ± 7.7
	**Case**	1260	456	804	55.5 ± 12.9	12.3 ± 7.8
**Herpes labialis**	**Control**	3560	2142	1418	51.6 ± 21.4	15.2 ± 7.1
	**Case**	356	223	133	51.3 ± 18.7	15.2 ± 6.9

We set out to replicate associations with common infections in Tian’s 23andMe GWAS study [11] which included 23 phenotypes; of those, we studied phenotypes which could be defined by ICD codes and for which we had more than 100 cases (Supplementary Table 2). These were urinary tract infection (UTI), pneumonia, chronic sinus infections, otitis media, candidiasis, streptococcal pharyngitis, herpes zoster, herpes labialis, hepatitis B, infectious mononucleosis, and tuberculosis (TB) or a positive TB test. We also included hepatitis C, a common infection that was not included in Tian’s report. The ICD diagnosis codes included in each phenotype are shown in Supplementary Table 1. For each candidate infectious disease, individuals with 2 or more codes for the phenotype on different days were considered as cases for the disease [16]. Individuals with only 1 mention of ICD code related to the disease were excluded from the analysis of that candidate infectious disease. We selected controls from individuals with no ICD codes for the candidate disease and matched these with cases of the infectious disease using year of birth, sex, and years of first and most recent EHR. We chose the matching factors to minimize important imbalances that could occur between case and control groups and thus reduce potential confounding; for example, we matched cases and controls for age and length of EHR because younger individuals and those with shorter EHRs have less time in which to accumulate clinical diagnoses, and we matched for sex because for some illnesses (.e.g., UTI) there are marked differences in prevalence among men and women. We matched controls to cases 5:1 for UTI, pneumonia, candidiasis, chronic sinus infection, otitis media, and hepatitis C. For infections with less than 1000 cases (streptococcal pharyngitis, herpes zoster, hepatitis B, infectious mononucleosis, TB or a positive TB test, and herpes labialis), we matched controls to cases 10:1(Table 1). For phenotypes with more than 1000 cases, we chose 1:5 case–control ratios based on statistical power calculations. For phenotypes with fewer than 1000 cases, we chose a 1:10 case–control ratio to take advantage of the additional small increase in power this provided for less frequent phenotypes [17].

In preliminary analyses, and as reported by others [18], we found that patients with cystic fibrosis (CF) contributed a strong genetic signal to pneumonia and chronic sinus infection; thus, to limit confounding by a CF genetic signal, we removed individuals with CF diagnosis codes (Supplementary Table 1) from the analyses of pneumonia and chronic sinus infection.

### Genotyping and SNP imputation

Genotyping was performed on the Infinium Multi-Ethnic Genotyping Array (MEGAchip). We took necessary technical measure to control genotyping quality and excluded DNA samples with (1) per-individual call rate < 95%; (2) mismatch between reported gender and X-chromosome zygosity; or (3) unexpected duplication. We performed whole genome imputation using the Michigan Imputation Server [19] with the Haplotype Reference Consortium, version r1.1 [20, 21] as reference. Principal components for ancestry (PCs) were calculated using common variants (MAF > 1%) with high variant call rate (> 98%); we excluded variants in linkage and regions known to affect PCs [HLA region on chromosome 6, inversion on chromosome 8 (8,135,000–12,000,000), and inversion on chromosome 17 (40,900,000–45,000,000); GRCh37 build]. Tian previously reported 28 genetic variants significantly associated with the infections we tested in BioVU; of these, 23 were directly available in our dataset or had another variant (within 500 kb) in high linkage disequilibrium (LD) using information for European ancestry population in the 1000 genomes database (*R*^2^ > 0.9, except for rs73015965 which was in LD with rs73027818 with *R*^2^ = 0.7) [22, 23].

### Statistical analysis

#### Genome-wide association study

We used SAIGE [24] to test associations between genotypes and risk of candidate infectious diseases using logistic regression assuming additive allelic effects and adjustment for sex, year of birth, year of first clinical visit, EHR length, and 10 PCs of ancestry to account for residual population structure [25]. Then, we conducted post-analysis quality control using EASYQC [26] to exclude (1) poorly imputed variants with r^2 ^value of < 0.3, (2) variants with minor allele frequency (MAF) < 0.5%, (3) variants with MAF different from the HRC reference panel (MAF differences > 0.3), and (4) variants significantly derived from Hardy–Weinberg equilibrium (HWE, *p* < 1 × 10^–6^). As we consider each infection an independent phenotype, we applied the standard GWAS Bonferroni correction cut-off and considered a *P*-value of less than 5 × 10^–8^ as significant.

#### Transcriptome wide association study (TWAS)

We conducted transcriptome analysis using PrediXcan (https://github.com/hakyimlab/PrediXcan) [27] with summary statistics from GWAS analyses. We leveraged all 49 available reference tissues from GTEx version 8. One approach would be to use organ- or tissue-specific prediction models, such as lung for pneumonia. However, because of the strong correlations across tissues in the genetic architecture for the regulation of gene expression (largely a function of the cell types making up that tissue), it is statistically powerful and thus we chose to utilize information from the tissues with the highest quality prediction performance or construct cross-tissue model. We also conducted cross-tissue transcriptomic analyses using MultiXcan and meta-analyzed all available tissue-based tests [28]. *P*-values of less than 2.5 × 10^–6^ (0.05/20000 genes) were considered significant.

#### Phenome-wide association studies (PheWAS)

PheWAS was conducted to identify clinical phenotypes that associate with infection-related genetic variants either reported by Tian et al. (variants in Table 2) or identified in current study [29, 30]. Specifically, we grouped each individual’s ICD codes into PheCodes following an established protocol [31, 32]. To be a case for each PheCode, an individual needs to have relevant ICD codes on at least 2 different days. Controls were individuals with no relevant ICD codes. Individuals with only one occurrence of a relevant code were excluded from the analyses. In a cohort of 65,592 white individuals, we analyzed a total of 1739 PheCodes with more than 20 cases. P-values of less than 2.9 × 10^–5^ (0.05/1739) were considered significant.

**Table 2:** Replication of previous GWAS associations from Tian *et al.* report

Phenotype	Cytoband	Gene_context	From Tian et al. report			From BioVU replication							
			**Variants**	**OR/ effectb**	**95%CI**	**SNP in BioVU**	**N**	**Effect allele**	**Other allele**	**EAF**	**OR**	**95% CI**	**P**
Herpes zoster	6p21.33	HLA	rs2523591	1.14	(1.13–1.16)	rs2523591	8261	G	A	0.59	1.01	(0.90–1.12)	0.88
	9p21.3	IFNA21	rs7047299	1.07	(1.06–1.09)	rs7047299	8261	A	G	0.56	1.18	(1.06–1.32)	0.0026^#^
Herpes labialis	6p21.33	HLA	rs885950	1.08	(1.07–1.09)	rs885950	3916	C	A	0.45	0.99	(0.85–1.16)	0.91^#^
Infectious mononucleosis	6p21.33	HLA	rs2596465	1.08	(1.06–1.09)	rs2596465	1276	T	C	0.45	1.17	(0.89–1.54)	0.26^#^
Hepatitis B	6p21.32	HLA	rs9268652	1.32	(1.25–1.38)	rs9268652	2453	G	A	0.76	1.01	(0.81–1.28)	0.9
Tuberculosis or a positive TB test	6p21.32	HLA	rs2894257	1.36	(1.33–1.39)	-	-	-	-	-	-	-	-
Streptococcal pharyngitis^*^	6p21.33	HLA	rs1055821	0.08	(0.06–0.09)	-	-	-	-	-	-	-	-
	1p36.23	ERRFI1–-[]–-SLC45A1	rs35395352	0.03	(0.03–0.04)	rs7548511	10,967	A	G	0.7	1.02	(0.92–1.13)	0.73
Pneumonia	6p21.33	HLA	rs3131623	1.1	(1.09 − 1.12)	rs3131623	38,310	T	A	0.84	1.01	(0.96–1.07)	0.67
Candidiasis^*^	18q12.1	DSG1	rs200520431	0.11	(0.09 − 0.12)	-	-	-	-	-	-	-	-
	14q23.1	PRKCH	rs2251260	0.05	(0.04 − 0.06)	rs2251260	13,212	T	C	0.19	0.99	(0.90–1.08)	0.82
	14q32.2	[]–-C14orf177	rs7161578	0.04	(0.03 − 0.04)	rs7161578	13,212	T	C	0.39	0.98	(0.91–1.05)	0.5
UTI^*^	8q24.3	JRK-[]-PSCA	rs2976388	0.04	(0.04 − 0.05)	rs2976388	56,154	G	A	0.57	1.02	(0.98–1.05)	0.32
	15q15.3	FRMD5	rs146906133	0.38	(0.32 − 0.45)	-	-	-	-	-	-	-	-
Otitis media	19q13.33	FUT2	rs681343	1.11	(1.1–1.12)	rs681343	14,604	C	T	0.51	1.05	(0.98–1.12)	0.14
	22q11.21	TBX1	rs1978060	1.09	(1.08–1.1)	rs1978060	14,604	G	A	0.59	1.03	(0.96–1.10)	0.45
	10p12.1	RAB18–[]–MKX	rs2808290	1.07	(1.07–1.08)	rs2808290	14,604	C	T	0.51	1.09	(1.02–1.16)	0.0096
	15q26.2	SPATA8–-[]–-LINC00923	rs7174062	1.08	(1.07–1.09)	rs7174062	14,604	G	A	0.73	1.03	(0.96–1.11)	0.38
	6p21.32	HLA	rs4329147	1.11	(1.09–1.13)	rs9270656	14,604	A	C	0.85	1.08	(0.99–1.18)	0.07
	9q34.2	ABO	rs8176643	1.06	(1.05–1.07)	rs600038	14,604	C	T	0.21	1.02	(0.94–1.10)	0.64^#^
	2p16.1	EFEMP1	rs1802575	1.09	(1.07–1.1)	rs1802575	14,604	G	C	0.87	0.13	(0.89–1.08)	0.72^#^
	2p24.1	NT5C1B-RDH14–-[]–-OSR1	rs5829676	1.06	(1.05–1.07)	rs11694107	14,604	A	G	0.4	1.05	(0.98–1.12)	0.16^#^
	11q13.3	FGF3–-[]–ANO1	rs72931768	1.09	(1.07–1.1)	rs72931768	14,604	G	C	0.89	0.11	(0.91–1.10)	0.99^#^
	7q11.22	AUTS2	rs35213789	1.06	(1.05–1.07)	rs35213789	14,604	C	T	0.74	0.26	(0.97–1.11)	0.31^#^
	7q22.3	CDHR3	rs114947103	1.07	(1.06–1.08)	rs114947103	14,604	C	T	0.18	1.09	(1.00–1.18)	0.0407^#^
	8q22.2	NIPAL2–[]–KCNS2	rs13281988	1.06	(1.05–1.07)	rs13281988	14,604	C	G	0.3	1.05	(0.98–1.12)	0.2^#^
	3p21.31	BSN	rs67035515	1.07	(1.05–1.08)	-	-	-	-	-	-	-	-
	6q26	PLG	rs73015965	1.43	(1.34–1.53)	rs73027818	14,604	T	C	0.01	1.44	(0.89–2.33)	0.14

^*^For streptococcal pharyngitis, candidiasis and UTI, Tian et al. used frequency of those infections as quantitative traits, while we identified cases/controls and used them as binary traits

^#^In the post-analysis power calculation there was less than 50% power to detect the odds ratios reported by Tian et al. [11]

We conducted a post-analysis power calculation to evaluate our ability to detect the odds ratios detected in the case–control phenotypes from Tian *et al.’s.* report, including herpes zoster (OR 1.07–1.14), herpes labialis (OR 1.08), infectious mononucleosis (OR 1.08), hepatitis B (OR 1.32), pneumonia (OR 1.1), and otitis media (OR 1.06 – 1.43). We could not run the power calculation for (1) continuous traits in Tian’s report, such as streptococcal pharyngitis, candidiasis, and UTI (because we applied a case–control study design); and (2) associations with variants unavailable in our cohort, such as tuberculosis (or a positive TB test). We used Genetic Association Study (GAS) power calculator [33].

### Replication of top infection hits with other clinical phenotypes

We also searched GWAS hits from the current study in the PheWeb database (http://pheweb.sph.umich.edu/) to test whether the identified top hits were associated with other clinical phenotypes from existing GWAS and PheWAS [34]. In addition, we investigated whether the identified GWAS hits for COVID-19 susceptibility or severity also contributed to susceptibility to pneumonia by querying our analysis of patients with pneumonia (none of whom had COVID-19).

## Results

### Study cohort

We identified cases and matched controls for 12 common infections, including 11 infections included in the Tian paper [11]. The number of cases ranged from 102 (TB or positive TB test) to 9359 (UTI) Table 1.

### Replication of previous GWAS of common infections

We replicated 3 associations with *p* <  = 0.05 and the same direction of effect as Tian’s report: herpes zoster with the A allele of rs7047299(*IFNA21* gene, odds ratio [OR], 1.18; 95% confidence interval [CI], [1.06–1.32]; *p* = 0.0026) and the C allele of rs2808290 (close to *MKX* gene, OR, 1.09; 95% CI [1.02–1.16]; *p* = 0.0096); and otitis media with the C allele of rs114947103 (*CDHR3* gene, OR, 1.09; 95% CI [1.00–1.18]; *p* = 0.0407) (Table 2).

### Phenome-wide association studies (PheWAS) of previous GWAS hits of common infections

We conducted PheWAS for the genetic variants in Tian *et al.’s* report and found 92 significant associations with clinical phenotypes (Supplementary Table 3, *p* < 2.9 × 10^–5^). Relating to infections, rs3131623 in *HLA* gene region was associated with chronic hepatitis infection (*p* = 5.07 × 10^–7^), and rs600038 in *ABO* gene region was associated with candidiasis (*p* = 2.35 × 10^–5^). Furthermore, 43 out of the 92 associations related to diabetes or diabetes related phenotypes and several in the HLA region associated with autoimmune diseases (Supplementary Table 3, Supplementary Fig. 1).

### New associations between genetic variants and the risk of common infections

**Fig. 1:**
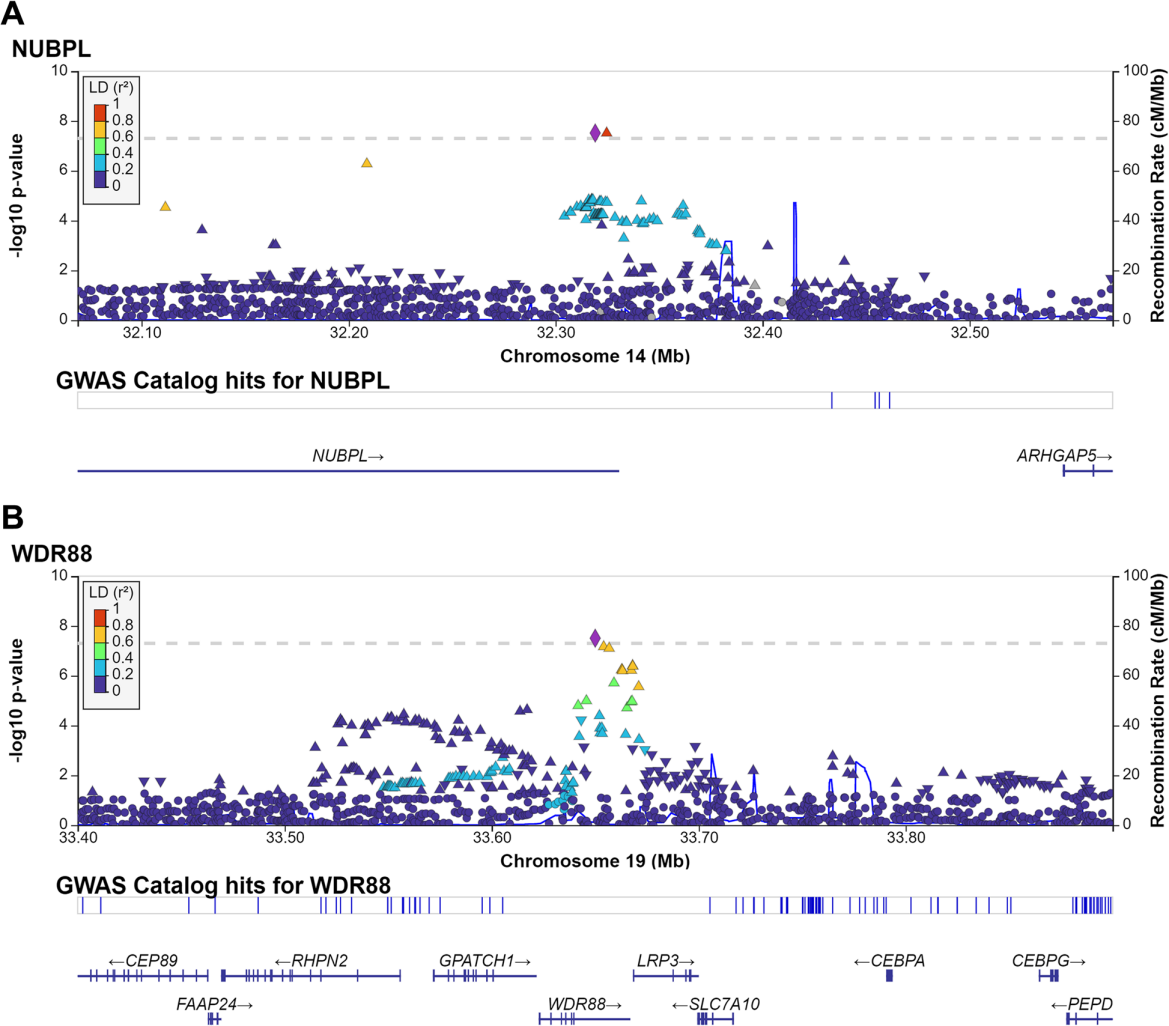
Regional plots for 2 loci that significantly associated with common infections. The color of the single nucleotide polymorphisms (SNPs) is based on the linkage disequilibrium with the lead SNP (purple). Reference sequence genes in the region are shown on the bottom. cM/Mb indicates centimorgan/mega base pair. (A) Regional plots for associations between NUBPL locus and otitis media. (B) Regional plots for associations between LRP3/WDR88 locus and candidiasis

We identified 3 new loci significantly associated with infections. (Table 3, Fig. 1, Supplementary Fig. 2) Two variants in *nucleotide binding protein like* (*NUBPL*) gene, the G allele of rs113235453 (OR, 1.50; 95% CI [1.30–1.73]; *p* = 3.04 × 10^–8^) and the A allele of rs74633202 (OR, 1.50; 95% CI [1.30–1.73]; p = 3.05 × 10^–8^) were associated with increased risk of otitis media. The T allele of rs10422015 in *WD repeat-containing protein 88* (*WDR88*) was associated with the increased risk of candidiasis (OR, 1.31; 95% [1.19–1.44]; *p* = 3.11 × 10^–8^) (Table 3).

**Table 3:** Significant associations between genetic variants and common infections

Phenotype	Cytoband	Gene context	rsNumber	Effect allele	Reference allele	Effect allele frequency	Odd Ratio	95% Confidence Interval	*p*-value
**Otitis media**	14q12	NUBPL	rs113235453	G	A	0.05	1.50	[1.30, 1.73]	3.04E-08
	14q12	NUBPL	rs74633202	A	C	0.05	1.50	[1.30, 1.73]	3.05E-08
**Candidiasis**	19q13.11	WDR88	rs10422015	T	G	0.15	1.31	[1.19, 1.44]	3.11E-08

Those variants were not significant in Tian *et al.* report. (not among top 8000 associated variants)

### Associations between the risk of common infections and the genetically predicted gene expression

In TWAS for the 12 infections studied, we found significant associations between elevated risk of (1) otitis media and genetically predicted increased expression of *solute carrier family 30 member 9* gene (*SLC30A9*, zscore = 4.93, *p* = 8.06 × 10^–7^) in brain nucleus accumbens basal ganglia; (2) candidiasis and the genetically predicted increased expression of *LDL receptor related protein 3* gene (*LRP3*, largest zscore 5.68, smallest *p*-value = 1.34 × 10^–8^) in tissues including esophagus mucosa, brain spinal cord cervical, artery, spleen, prostate, adrenal gland, and minor salivary gland; (3) candidiasis and the genetically predicted increased expression of *WDR88* (largest z-score 5.54, smallest *p*-value = 3.11 × 10^–8^) in liver and brain cortex; (4) hepatitis B and the genetically predicted decreased expression of *adipogenesis associated Mth938 domain containing* gene (*AAMDC*, smallest z-score -4.89, smallest *p*-value = 1.02 × 10^–6^) in heart atrial appendage and skin (not sun exposed, Table 4). Additionally, several of these four disease-transcriptome associations were nominally significant (*p* < 10^–5^) in several other tissues (Supplementary Table 4). In the cross-tissue analysis, only the association between increased risk of candidiasis and the genetically predicted increased expression of *WDR88* was significant (*p*-value = 1.83 × 10^–6^).

**Table 4:** Significant associations between genetically-determined gene expression and common infections

Phenotype	Tissue	gene_name	zscore	Effect size	*P* value	var_g^*^	n_snps_used^#^
**Otitis media**	**Brain_Nucleus_accumbens_basal_ganglia**	SLC30A9	4.93	1.03	8.06E-07	0.014934	2
**Candidiasis**	**Esophagus_Mucosa**	LRP3	5.68	2.13	1.34E-08	0.005041	2
	**Liver**	WDR88	5.54	0.55	3.11E-08	0.060999	1
	**Brain_Spinal_cord_cervical_c-1**	LRP3	5.17	6.50	2.34E-07	0.000383	2
	**Artery_Coronary**	LRP3	5.13	2.67	2.82E-07	0.0029	2
	**Spleen**	LRP3	5.09	2.04	3.57E-07	0.004629	2
	**Prostate**	LRP3	5.07	3.39	3.91E-07	0.001917	1
	**Adrenal_Gland**	LRP3	5.07	2.33	3.91E-07	0.003467	1
	**Minor_Salivary_Gland**	LRP3	5.07	2.36	3.91E-07	0.003164	1
	**Brain_Cortex**	WDR88	4.76	0.39	1.95E-06	0.087545	1
**Hepatitis B**	**Heart_Atrial_Appendage**	AAMDC	-4.81	-2.59	1.51E-06	0.019472	3
	**Skin_Not_Sun_Exposed_Suprapubic**	AAMDC	-4.89	-3.35	1.02E-06	0.015421	3

^*^var_g: variance of the gene expression

^#^n_snps_used: number of snps from GWAS that got used in S-PrediXcan analysis

### Associations between lead GWAS hits and other clinical phenotypes

We searched PheWeb and conducted PheWAS in BioVU for the lead GWAS hits in the current study (rs113235453 for otitis media and rs10422015 for candidiasis) for their associations with other clinical phenotypes. Both variants were significantly associated with non-infectious conditions: rs113235453 with non-traumatic intracranial hemorrhage (*p* = 6.4 × 10^–7^) and rs10422015 with heel bone mineral density T-score (*p* = 1.1 × 10^–15^). For infection-related phenotypes there were a few suggestive associations: (1) rs113235453 was associated with use of antibiotics for bacterial infections (co-Amoxiclav) (*p* = 2.4 × 10^–4^), and (2) rs10422015 with cough (*p* = 4.2 × 10^–4^) or postoperative infection (*p* = 4.4 × 10^–4^). In the PheWAS using BioVU samples, there were no significant associations with these two variants; however, leading associations included infection-related phenotypes such as hepatitis, candidiasis and abnormal findings on the examinations of urine. (Supplementary Table 5).

### Associations between top COVID-19 hits and the risk of pneumonia

When we examined 13 loci associated with COVID-19 [35] and susceptibility to pneumonia in our cohort we found an association between the C allele of rs13050728 in *IFNAR2* (Interferon Alpha and Beta Receptor Subunit 2) gene and lower risk of developing pneumonia (OR 0.94, 95%CI [0.90–0.98], *p* = 0.0028, Table 5), an observation directionally similar to that for severity of COVID-19 [35].

**Table 5:** Associations between loci associated with COVID-19 susceptibility and severity^*^ hits and the risk of pneumonia (*N* = 38,310)

chr	RSID	Reference allele	Effect allele	Effect allele frequency	*P*-value	Effect size (beta)	Nearest gene
3	**rs2271616**	G	T	0.138	0.0960	0.0482	SLC6A20
3	**rs10490770**	T	C	0.070	0.8474	-0.0076	LZTFL1
3	**rs11919389**	T	C	0.349	0.6783	-0.0086	RPL24
6	**rs1886814**	-	-	-	-	-	
8	**rs72711165**	T	C	0.010	0.1428	-0.1428	TMEM65
9	**rs912805253**	-	-	-	-	-	
12	**rs10774671**	G	A	0.646	0.6174	0.0103	OAS1
17	**rs1819040**	-	-	-	-	-	
17	**rs77534576**	C	T	0.028	0.2274	0.0718	TAC4
19	**rs2109069**	G	A	0.315	0.9981	0.0001	DPP9
19	**rs74956615**	T	A	0.023	0.9783	-0.0021	RAVER1
19	**rs4801778**	G	T	0.186	0.1619	0.0353	PLEKHA4
21	**rs13050728**	T	C	0.672	0.0028	-0.0631	IFNAR2

^*^COVID19 susceptibility and severity loci are from previous report [35]

## Discussion

The current study of the genetics of 12 common infections replicated 3 associations from previous 23andMe GWAS findings. Additionally, 2 new loci (from GWAS) and altered genetically predicted expression of 4 genes (from TWAS) were associated with altered susceptibility to infection. Last, one of the alleles identified with reduced severity risk of COVID-19 was associated with reduced risk of pneumonia.

The link between the innate immune response and infection is well established [36]. Thus the replicated association between a variant in *IFNA21* and herpes zoster previously reported by Tian et al., is of interest. *IFNA21* encodes a type I interferon, which binds to interferon alpha receptor and activates innate immune responses. Further indication of the importance of this pathway is the association between an *IFNAR2* variant and susceptibility to pneumonia. This variant was reported as one of the top hits associated with both COVID-19 susceptibility and severity [35]. By leveraging summary statistics from a COVID-19 GWAS and a Mendelian randomization approach, a recent drug repurposing study prioritized IFNAR2 as one of top two candidate drug targets for early management of COVID-19 [37]. Indeed, interferon and drugs that target interferon receptors have been used to treat infectious diseases [38–40]. Currently, there are phase II clinical trials testing interferons for COVID-19 infection, and the results of clinical trials are awaited [41–43]. In PheWAS analyses of the 23andMe variants reported to be significantly associated with infection [11], we observed associations 92 significant PheWAS associations with 6 SNPs (rs885950, rs2523591, rs2596465, rs3131623, rs9268652, rs9270656) associated with 43 clinical phenotypes related to diabetes. These SNPs are located in genes that associated with type 1 or type 2 diabetes in previous GWAS (Supplementary Table 3). Impaired glucose regulation is associated with an elevated risk of many infections, including hepatitis [44, 45], and SARS-CoV-2.[46] Future studies will need to determine if variants predispose to infection directly or through associations with co-morbidities that increase risk of infection.

Additional to replicating variants from the 23andMe study, we identified several novel variants within *NUBPL* gene region associated with otitis media. The lead hit, rs113235453, has previously been associated with heart rate in patients with heart failure and reduced ejection fraction [47]. *NUBPL* encodes nucleotide binding protein-like on chromosome 14q12, and functional variants in the gene are associated with mitochondrial complex I deficiency and linked to leukoencephalopathy and Parkinson’s disease [48, 49]. Infection is a common cause of morbidity in children with mitochondrial diseases; however, it is unclear if variation in *NUBPL* could influence the risk of infection through its role in mitochondrial complex I deficiency.

An additional new observation was an association between the *WDR88/LRP3* region and the risk of candidiasis; further, TWAS also showed that the genetically determined expression of WDR88 and LRP3 in a variety of tissues associated with altered risk of candidiasis. The underlying mechanisms are not obvious. *WDR88* has previously been associated with schizophrenia [50]; however, the function of the gene remains unclear. The association of LRP3 expression with candida infection in esophageal mucosa was interesting because the esophagus is a well-described site of candida infection. *LRP3* encodes LDL receptor related protein 3, which is involved in the internalization of lipophilic molecules [51], but whether LRP3 could affect the risk of candida infection through this mechanism is not known.

Another new observation was the association between genetically predicted expression of SLC30A9 and altered risk of otitis media. *SLC30A9* encodes solute carrier family 30 member 9, which acts as a zinc transporter involved in intracellular zinc homeostasis [52]. In vitro experiments suggested that SLC30A9 interacted with human influenza A virus [53]; therefore, SLC30A9 might alter the risk of infection through its role in recognition and binding to pathogens. Although many of the HLA/infection associations reported by Tian *et al.*, did not replicate, there was a close-to-significant association between HLA-DQB1 and the risk of infectious mononucleosis (*p* = 2.59 × 10^–6^, Supplementary Table 4). The HLA region is critical for host response to infection. Future studies using large cohorts are needed to better understand the role of the HLA region.

The study has many strengths: the use of diagnoses made by providers to identify cases of infection in a large EHR database; matching of cases and controls to limit confounding; performance of transcriptome analysis using GWAS summary statistics to further understand the associations between host genetics and common infections; and an ability to test the associations between known loci affecting COVID-19 and the risk of pneumonia. There are also limitations. First, while power was good for most infections, there was limited power to detect small odds ratios for low-frequency variants and less common phenotypes, such as TB/positive TB tests (N of cases, 102) and mononucleosis (N of cases, 116). Additional studies will be required for less common infections. Second, ICD codes serve primarily billing purposes and are not recorded by clinicians to facilitate research; misclassification or under or over coding of conditions may occur. Also, the study was conducted in White patients. For many infections the number of cases in Black patients was too small for GWAS and will require additional studies. Third, we matched controls to cases on age, sex, and year of first and last clinical visits. However, the potential for misclassification of controls remains. There is always a possibility that the control population was enriched for some co-segregating factors of infections. Future study is needed to validate our observations. Fourth, in TWAS analyses, the gene expression predicted by a single SNP may be less robust than those predicted by multiple variants. However, many examples show that a single SNP can contribute significantly to gene expression (e.g., LPA and rs10455872, CETP and rs18000777 etc.). In Table 3, although *LRP3* gene and *WDR88* gene expressions were both predicted using one SNP, it is worth noting that the same significant association was observed in multiple tissues. The replication of this LRP3/candidiasis and WDR88/candidiasis association in various tissues suggests that there may be mechanisms common across tissues. Response to infection can affect multiple organs, thus we presented data from all available tissues for readers. Lastly, the novel loci we identified were not detected in Tian’s study; [11] several study design factors may account for these differences among studies. For example, we studied a population obtaining medical care in a large hospital whereas Tian studied a presumably healthier population who sought a genetic test; we matched controls to cases whereas Tian did not; and the studies employed different disease phenotype definitions (diagnosis billing codes vs. self-report) that may vary in sensitivity and specificity. Also, environmental, social, and economic factors vary among populations, and neither Tian’s report nor our study included these potentially important factors as covariates. As the All of Us (AoU) project develops and collects information about those factors and links them to EHR and genetic data, such studies will be possible.

In conclusion, we conducted GWAS and TWAS for 12 common infectious diseases and identified novel genetic contributors to the susceptibility of infection diseases.

## Supplementary Information

**Additional file1:** **Supplementary Table 1.** ICD codes used to definephenotypes.

**Additional file 2:** **Supplementary Table 2.** List of phenotypes studied from 23andMe paper [11] and ICD codes. **Supplementary Table 3.** PheWAS of previous GWAS associations from Tian *et al*. report. **Supplementary Table 4. **Associations between genetically predicted gene expression and altered risk of common infections (*p*<1×10^-5^). **Supplementary Table 5.** PheWAS of genetic variants that were associated with common infections in BioVU (suggestive p-value cutoff, 0.001).

**Additional file 3:** **Supplementary Figure 1.** Manhattan plots of Phenome-wide associations studies.

**Additional file 4:** **Supplementary Figure 2.** Manhattan plots and Q-Q plots of GWAS results.

## Data Availability

The summary statistics is available in GWAS catalog (https://www.ebi.ac.uk/gwas/, GCP ID GCP000359).
