# Supplementary material for: Genome-wide association analyses of common infections in a large practice-based biobank
Source: BMC Genomics. 2022 Sep 27;23:672. doi: 10.1186/s12864-022-08888-9 (PMC9512962; doi:10.1186/s12864-022-08888-9)
Supplement: Supplementary file 1 — Additional file1: Supplementary Table 1. ICD codes used to definephenotypes. [file 12864_2022_8888_MOESM1_ESM.docx]

**Supplementary Table 1. ICD codes that used to identify phenotypes**

| Phenotypes | ICD9CM | ICD10CM |
| --- | --- | --- |
| UTI | 590.00,590.01,590.10,590.11,590.80,590.81,595.0,599.0,646.60,646.61,646.62,646.63,646.64,771.82 | A02.25,N10,N11.0,N11.1,N13.6,N30.00,N30.01,N39.0,O03.38,O03.88,O04.88,O07.38,O08.83,O23.00,O23.01,O23.02,O23.03,O23.10,O23.11,O23.12,O23.13,O23.20,O23.21,O23.22,O23.23,O23.30,O23.31,O23.32,O23.33,O23.40,O23.41,O23.42,O23.43,O86.20,O86.21,O86.22,O86.29,P39.3 |
| Cold sores | 54.9 | B00.9 |
| Ear Infection | 055.2,381.00,381.01,381.02,381.03,381.04,381.05,381.06,381.10,381.19,381.20,381.29,381.3,381.4,382.00,382.01,382.02,382.1,382.2,382.3,382.4,382.9 | A38.0,B05.3,B37.84,H65.00,H65.01,H65.02,H65.03,H65.04,H65.05,H65.06,H65.07,H65.111,H65.112,H65.113,H65.114,H65.115,H65.116,H65.117,H65.119,H65.191,H65.192,H65.193,H65.194,H65.195,H65.196,H65.197,H65.199,H65.20,H65.21,H65.22,H65.23,H65.30,H65.31,H65.32,H65.33,H65.411,H65.412,H65.413,H65.419,H65.491,H65.492,H65.493,H65.499,H65.90,H65.91,H65.92,H65.93,H66.001,H66.002,H66.003,H66.004,H66.005,H66.006,H66.007,H66.009,H66.011,H66.012,H66.013,H66.014,H66.015,H66.016,H66.017,H66.019,H66.10,H66.11,H66.12,H66.13,H66.20,H66.21,H66.22,H66.23,H66.3X1,H66.3X2,H66.3X3,H66.3X9,H66.40,H66.41,H66.42,H66.43,H66.90,H66.91,H66.92,H66.93,H67.1,H67.2,H67.3,H67.9,J10.83,J11.83 |
| Hepatitis B | 070.20,070.21,070.22,070.23,070.30,070.31,070.32,070.33,070.42,070.52,V02.61 | B16.0,B16.1,B16.2,B16.9,B17.0,B18.0,B18.1,B19.10,B19.11 |
| Hepatitis C | 070.41,070.44,070.51,070.54,070.70,070.71,V02.62 | B17.10,B17.11,B18.2,B19.20,B19.21 |
| infectous mononucleosis | 75 | B27.00,B27.01,B27.02,B27.09,B27.10,B27.11,B27.12,B27.19,B27.80,B27.81,B27.82,B27.89,B27.90,B27.91,B27.92,B27.99 |
| pneumonia | 003.22,052.1,055.1,073.0,115.05,115.15,115.95,480.0,480.1,480.2,480.3,480.8,480.9,481,482.0,482.1,482.2,482.30,482.31,482.32,482.39,482.40,482.41,482.42,482.49,482.81,482.82,482.83,482.84,482.89,482.9,483.0,483.1,483.8,484.1,484.3,484.5,484.6,484.7,484.8,485,486,487.0,488.01,488.11,488.81,997.31,997.32,V12.61 | A01.03,A02.22,A22.1,A37.01,A37.11,A37.81,A37.91,A48.1,A50.04,A54.84,A70,B01.2,B05.2,B06.81,B25.0,B39.0,B44.0,B96.0,B96.1,J09.X1,J10.00,J10.01,J10.08,J11.00,J11.08,J12.0,J12.1,J12.2,J12.3,J12.81,J12.89,J12.9,J13,J14,J15.0,J15.1,J15.20,J15.211,J15.212,J15.29,J15.3,J15.4,J15.5,J15.6,J15.7,J15.8,J15.9,J16.0,J16.8,J17,J18.0,J18.1,J18.2,J18.8,J18.9,J20.0,J85.1,J95.851,P23.0,P23.1,P23.2,P23.3,P23.4,P23.5,P23.6,P23.8,P23.9,Z87.01 |
| shingles | 053.0,053.10,053.11,053.14,053.19,053.20,053.21,053.22,053.29,053.71,053.79,053.8,053.9 | B02.0,B02.1,B02.21,B02.24,B02.29,B02.30,B02.31,B02.32,B02.33,B02.34,B02.39,B02.7,B02.8,B02.9 |
| Chronic sinus infection | 473.0,473.1,473.2,473.3,473.8,473.9 | J32.0,J32.1,J32.2,J32.3,J32.4,J32.8,J32.9 |
| strep throat | 034.0,034.1 | J02.0,J03.00,J03.01 |
| Positive TB test | 010.00,010.01,010.02,010.03,010.04,010.05,010.06,010.10,010.11,010.12,010.13,010.14,010.15,010.16,010.80,010.81,010.82,010.83,010.84,010.85,010.86,010.90,010.91,010.92,010.93,010.94,010.95,010.96,011.00,011.01,011.02,011.03,011.04,011.05,011.06,011.10,011.11,011.12,011.13,011.14,011.15,011.16,011.20,011.21,011.22,011.23,011.24,011.25,011.26,011.30,011.31,011.32,011.33,011.34,011.35,011.36,011.40,011.41,011.42,011.43,011.44,011.45,011.46,011.50,011.51,011.52,011.53,011.54,011.55,011.56,011.60,011.61,011.62,011.63,011.64,011.65,011.66,011.70,011.71,011.72,011.73,011.74,011.75,011.76,011.80,011.81,011.82,011.83,011.84,011.85,011.86,011.90,011.91,011.92,011.93,011.94,011.95,011.96,012.00,012.01,012.02,012.03,012.04,012.05,012.06,012.10,012.11,012.12,012.13,012.14,012.15,012.16,012.20,012.21,012.22,012.23,012.24,012.25,012.26,012.30,012.31,012.32,012.33,012.34,012.35,012.36,012.80,012.81,012.82,012.83,012.84,012.85,012.86,013.00,013.01,013.02,013.03,013.04,013.05,013.06,013.10,013.11,013.12,013.13,013.14,013.15,013.16,013.20,013.21,013.22,013.23,013.24,013.25,013.26,013.30,013.31,013.32,013.33,013.34,013.35,013.36,013.40,013.41,013.42,013.43,013.44,013.45,013.46,013.50,013.51,013.52,013.53,013.54,013.55,013.56,013.60,013.61,013.62,013.63,013.64,013.65,013.66,013.80,013.81,013.82,013.83,013.84,013.85,013.86,013.90,013.91,013.92,013.93,013.94,013.95,013.96,014.00,014.01,014.02,014.03,014.04,014.05,014.06,014.80,014.81,014.82,014.83,014.84,014.85,014.86,015.00,015.01,015.02,015.03,015.04,015.05,015.06,015.10,015.11,015.12,015.13,015.14,015.15,015.16,015.20,015.21,015.22,015.23,015.24,015.25,015.26,015.50,015.51,015.52,015.53,015.54,015.55,015.56,015.60,015.61,015.62,015.63,015.64,015.65,015.66,015.70,015.71,015.72,015.73,015.74,015.75,015.76,015.80,015.81,015.82,015.83,015.84,015.85,015.86,015.90,015.91,015.92,015.93,015.94,015.95,015.96,016.00,016.01,016.02,016.03,016.04,016.05,016.06,016.10,016.11,016.12,016.13,016.14,016.15,016.16,016.20,016.21,016.22,016.23,016.24,016.25,016.26,016.30,016.31,016.32,016.33,016.34,016.35,016.36,016.40,016.41,016.42,016.43,016.44,016.45,016.46,016.50,016.51,016.52,016.53,016.54,016.55,016.56,016.60,016.61,016.62,016.63,016.64,016.65,016.66,016.70,016.71,016.72,016.73,016.74,016.75,016.76,016.90,016.91,016.92,016.93,016.94,016.95,016.96,017.00,017.01,017.02,017.03,017.04,017.05,017.06,017.10,017.11,017.12,017.13,017.14,017.15,017.16,017.20,017.21,017.22,017.23,017.24,017.25,017.26,017.30,017.31,017.32,017.33,017.34,017.35,017.36,017.40,017.41,017.42,017.43,017.44,017.45,017.46,017.50,017.51,017.52,017.53,017.54,017.55,017.56,017.60,017.61,017.62,017.63,017.64,017.65,017.66,017.70,017.71,017.72,017.73,017.74,017.75,017.76,017.80,017.81,017.82,017.83,017.84,017.85,017.86,017.90,017.91,017.92,017.93,017.94,017.95,017.96,018.00,018.01,018.02,018.03,018.04,018.05,018.06,018.80,018.81,018.82,018.83,018.84,018.85,018.86,018.90,018.91,018.92,018.93,018.94,018.95,018.96,137.0,137.1,137.2,137.3,137.4,371.05,647.30,647.31,647.32,647.33,647.34,V12.01,795.5,795.51,795.52 | A15.0,A15.4,A15.5,A15.6,A15.7,A15.8,A15.9,A17.0,A17.1,A17.81,A17.82,A17.83,A17.89,A17.9,A18.01,A18.02,A18.03,A18.09,A18.10,A18.11,A18.12,A18.13,A18.14,A18.15,A18.16,A18.17,A18.18,A18.2,A18.31,A18.32,A18.39,A18.4,A18.50,A18.51,A18.52,A18.53,A18.54,A18.59,A18.6,A18.7,A18.81,A18.82,A18.83,A18.84,A18.85,A18.89,A19.0,A19.1,A19.2,A19.8,A19.9,B90.0,B90.1,B90.2,B90.8,B90.9,J65,O98.011,O98.012,O98.013,O98.019,O98.02,O98.03,P37.0,Z86.11,R76.1,R76.11,R76.12 |
| yeast infection | 112.0,112.1,112.2,112.3,112.4,112.5,112.81,112.82,112.83,112.84,112.85,112.89,112.9,771.7 | B37.0,B37.1,B37.2,B37.3,B37.41,B37.42,B37.49,B37.5,B37.6,B37.7,B37.81,B37.82,B37.83,B37.84,B37.89,B37.9,P37.5 |
| Cystic fibrosis | 277.0, 277.00, 277.01, 277.02, 277.03, 277.09 | E84, E84.0, E84.1, E84.8, E84.9 |
