# Supplementary material for: Genome-wide association analyses of common infections in a large practice-based biobank
Source: BMC Genomics. 2022 Sep 27;23:672. doi: 10.1186/s12864-022-08888-9 (PMC9512962; doi:10.1186/s12864-022-08888-9)
Supplement: Supplementary file 2 — Additional file 2: Supplementary Table 2. List of phenotypes studied from 23andMe paper [11] and ICD codes. Supplementary Table 3. PheWAS of previous GWAS associations from Tian et al. report. Supplementary Table 4. Associations between genetically predicted gene expression and altered risk of common infections (p<1×10^-5^). Supplementary Table 5. PheWAS of genetic variants that were associated with common infections in BioVU (suggestive p-value cutoff, 0.001). [file 12864_2022_8888_MOESM2_ESM.pdf]

Supplementary Table 2. List of phenotypes studied using 23andMe data (Tian et al. paper) and ICD code.

23andMe	ICD Phenotype
chickenpox	NA
Shingles	herpes zoster
cold sores	herpes labialis
mononucleosis	infectious mononucleosis
mumps	NA
hepatitis B	hepatitis B
positive tuberculosis test results	positive tuberculosis test
strep throat	streptococcal pharyngitis
scarlet fever	NA
pneumonia	pneumonia
bacterial meningitis	NA
yeast infection	candidiasis
urinary tract infections	urinary tract infection
tonsillectomy	NA
childhood ear infections	otitis media
myringotomy	NA
plantar warts	NA
measles	NA
hepatitis A	NA
rheumatic fever	NA
common colds	NA
rubella	NA
chronic sinus infection	chronic sinus infection

**NA**	**hepatitis C**

Supplementary Table 3. PheWAS of previous GWAS associations from Tian *et al.* report.

snp	mapped to gene(s)	Cytoband	Associated infections in Tian *et al.*	Associated clinical phenotypes in BioVU							Observations from GWAS catalog
				Effect allele	PheCode	Description	OR	95% CI		p	
rs885950	POU5F1, HLA	6p21.33	Herpes labialis	A	250.1	Type 1 diabetes	0.84	0.80	0.89	2.07E-09	Variants in POU5F1 gene has been associated with diabetes. (PMID: 28869590)
rs885950	POU5F1, HLA	6p21.33	Herpes labialis	A	250.12	Type 1 diabetes with renal manifestations	0.75	0.66	0.86	1.72E-05	
rs885950	POU5F1, HLA	6p21.33	Herpes labialis	A	250.13	Type 1 diabetes with ophthalmic manifestations	0.71	0.62	0.82	3.29E-06	
rs885950	POU5F1, HLA	6p21.33	Herpes labialis	A	250.2	Type 2 diabetes	0.92	0.90	0.95	2.36E-07	
rs885950	POU5F1, HCG27, HLA	6p21.33	Herpes labialis	A	250.3	Insulin pump user	0.86	0.81	0.90	3.56E-08	
rs885950	POU5F1, HCG27, HLA	6p21.33	Herpes labialis	A	287.3	Thrombocytopenia	0.91	0.87	0.95	1.06E-05	Variants in POU5F1 gene has been associated with platelet cirt and mean platelet volume. (PMID: 32888494)
rs885950	POU5F1, HCG27, HLA	6p21.33	Herpes labialis	A	287.32	Secondary thrombocytopenia	0.84	0.78	0.91	2.70E-05	
rs885950	POU5F1, HCG27, HLA	6p21.33	Herpes labialis	A	557.1	Celiac disease	0.63	0.55	0.72	1.33E-12	
rs2523591	HLA-B	6p21.33	Herpes zoster	G	250.1	Type 1 diabetes	1.13	1.07	1.20	2.36E-05	Variants in HLA-B gene has been associated with diabetes.
rs2523591	HLA-B	6p21.33	Herpes zoster	G	250.11	Type 1 diabetes with ketoacidosis	1.44	1.25	1.66	3.93E-07	

rs2523591	HLA-B	6p21.33	Herpes zoster	G	250.12	Type 1 diabetes with renal manifestations	1.35	1.18	1.55	1.49E-05	(PMID: 33385400)
rs2523591	HLA-B	6p21.33	Herpes zoster	G	250.13	Type 1 diabetes with ophthalmic manifestations	1.43	1.23	1.67	2.59E-06	
rs2523591	HLA-B	6p21.33	Herpes zoster	G	335	Multiple sclerosis	1.20	1.11	1.29	7.61E-06	
rs2523591	HLA-B	6p21.33	Herpes zoster	G	557.1	Celiac disease	1.39	1.22	1.59	1.58E-06	
rs2523591	HLA-B	6p21.33	Herpes zoster	G	715.2	Ankylosing spondylitis	0.48	0.39	0.58	3.39E-13	Variants in HLA-B have been associated with Ankylosing spondylitis. (PMID: 30946743)
rs2596465	HLA, LINC01149	6p21.33	Infectious mononucleosis	T	250.1	Type 1 diabetes	0.85	0.80	0.90	1.17E-08	Variants within LINC01149 has been associated with age at onset of type 1 diabetes (PMID: 33179336)
rs2596465	HLA, LINC01149	6p21.33	Infectious mononucleosis	T	250.11	Type 1 diabetes with ketoacidosis	0.71	0.62	0.81	6.54E-07	
rs2596465	HLA, LINC01149	6p21.33	Infectious mononucleosis	T	250.21	Type 2 diabetes with ketoacidosis	0.72	0.62	0.83	1.54E-05	
rs2596465	HLA, LINC01149	6p21.33	Infectious mononucleosis	T	250.3	Insulin pump user	0.85	0.81	0.90	3.06E-08	
rs2596465	HLA, LINC01149	6p21.33	Infectious mononucleosis	T	250.7	Diabetic retinopathy	0.83	0.76	0.91	2.87E-05	
rs2596465	HLA, LINC01149	6p21.33	Infectious mononucleosis	T	335	Multiple sclerosis	1.20	1.11	1.29	2.12E-06	

rs2596465	HLA, LINC01149	6p21.33	Infectious mononucleosis	T	557.1	Celiac disease	0.59	0.51	0.67	1.54E-14	
rs2596465	HLA, LINC01149	6p21.33	Infectious mononucleosis	T	715.2	Ankylosing spondylitis	0.61	0.49	0.75	2.39E-06	
rs3131623	HLA, LINC01149	6p21.33	pneumonia	T	70.4	Chronic hepatitis	0.70	0.60	0.80	5.07E-07	
rs3131623	HLA, LINC01149	6p21.33	pneumonia	T	242.1	Graves' disease	0.65	0.57	0.75	2.09E-09	
rs3131623	HLA, LINC01149	6p21.33	pneumonia	T	244.4	Hypothyroidism NOS	0.90	0.86	0.94	5.96E-06	
rs3131623	HLA, LINC01149	6p21.33	pneumonia	T	245.21	Chronic lymphocytic thyroiditis	0.72	0.65	0.80	2.30E-09	
rs3131623	HLA, LINC01149	6p21.33	pneumonia	T	250.1	Type 1 diabetes	0.79	0.73	0.85	7.86E-11	Variants within LINC01149 has been associated with age at onset of type 1 diabetes. (PMID: 33179336)
rs3131623	HLA, LINC01149	6p21.33	pneumonia	T	250.11	Type 1 diabetes with ketoacidosis	0.66	0.56	0.77	2.67E-07	
rs3131623	HLA, LINC01149	6p21.33	pneumonia	T	250.12	Type 1 diabetes with renal manifestations	0.65	0.56	0.76	8.63E-08	
rs3131623	HLA, LINC01149	6p21.33	pneumonia	T	250.13	Type 1 diabetes with ophthalmic manifestations	0.63	0.53	0.74	5.07E-08	
rs3131623	HLA, LINC01149	6p21.33	pneumonia	T	250.14	Type 1 diabetes with neurological manifestations	0.74	0.65	0.84	4.14E-06	

rs3131623	HLA, LINC01149	6p21.33	pneumonia	T	250.21	Type 2 diabetes with ketoacidosis	0.66	0.55	0.79	3.50E-06	
rs3131623	HLA, LINC01149	6p21.33	pneumonia	T	250.3	Insulin pump user	0.80	0.74	0.86	3.88E-10	
rs3131623	HLA, LINC01149	6p21.33	pneumonia	T	250.7	Diabetic retinopathy	0.76	0.69	0.85	1.44E-06	
rs3131623	HLA, LINC01149	6p21.33	pneumonia	T	557.1	Celiac disease	0.37	0.32	0.42	5.06E-47	
rs3131623	HLA, LINC01149	6p21.33	pneumonia	T	575.1	Cholangitis	0.68	0.58	0.80	2.31E-06	
rs3131623	HLA, LINC01149	6p21.33	pneumonia	T	585.32	End stage renal disease	0.82	0.75	0.90	1.24E-05	
rs3131623	HLA, LINC01149	6p21.33	pneumonia	T	695.42	Systemic lupus erythematosus	0.69	0.62	0.77	8.81E-12	Variants in LINC01149 has been associated with Lupus. (PMID: 33272962)
rs3131623	HLA, LINC01149	6p21.33	pneumonia	T	709	Diffuse diseases of connective tissue	0.64	0.52	0.78	1.98E-05	
rs3131623	HLA, LINC01149	6p21.33	pneumonia	T	709.2	Sicca syndrome	0.71	0.62	0.81	9.82E-07	
rs3131623	HLA, LINC01149	6p21.33	pneumonia	T	709.4	Polymyositis	0.45	0.33	0.60	1.34E-07	

rs3131623	HLA, LINC01149	6p21.33	pneumonia	T	709.5	Dermatomyositis	0.47	0.37	0.61	2.54E-09	
rs9268652	HLA-DRA	6p21.32	Hepatitis B	A	244.4	Hypothyroidism NOS	0.90	0.86	0.93	4.99E-08	
rs9268652	HLA-DRA	6p21.32	Hepatitis B	A	245.21	Chronic lymphocytic thyroiditis	0.78	0.71	0.87	8.43E-06	
rs9268652	HLA-DRA	6p21.32	Hepatitis B	A	250	Diabetes mellitus	0.79	0.72	0.86	2.20E-07	variants wit HLA-DRA have been associated with type 1 diabetes. (PMID: 19430480)
rs9268652	HLA-DRA	6p21.32	Hepatitis B	A	250.1	Type 1 diabetes	0.68	0.63	0.73	2.15E-26	
rs9268652	HLA-DRA	6p21.32	Hepatitis B	A	250.11	Type 1 diabetes with ketoacidosis	0.31	0.25	0.39	1.89E-23	
rs9268652	HLA-DRA	6p21.32	Hepatitis B	A	250.12	Type 1 diabetes with renal manifestations	0.47	0.39	0.57	9.94E-15	
rs9268652	HLA-DRA	6p21.32	Hepatitis B	A	250.13	Type 1 diabetes with ophthalmic manifestations	0.37	0.30	0.47	1.61E-17	
rs9268652	HLA-DRA	6p21.32	Hepatitis B	A	250.14	Type 1 diabetes with neurological manifestations	0.65	0.57	0.74	2.31E-10	
rs9268652	HLA-DRA	6p21.32	Hepatitis B	A	250.2	Type 2 diabetes	0.92	0.89	0.95	2.19E-06	
rs9268652	HLA-DRA	6p21.32	Hepatitis B	A	250.21	Type 2 diabetes with ketoacidosis	0.60	0.50	0.73	3.75E-07	
rs9268652	HLA-DRA	6p21.32	Hepatitis B	A	250.3	Insulin pump user	0.74	0.69	0.79	2.23E-18	
rs9268652	HLA-DRA	6p21.32	Hepatitis B	A	250.7	Diabetic retinopathy	0.74	0.67	0.83	9.24E-08	
rs9268652	HLA-DRA	6p21.32	Hepatitis B	A	335	Multiple sclerosis	1.86	1.72	2.01	8.37E-54	
rs9268652	HLA-DRA	6p21.32	Hepatitis B	A	341	Other demyelinating	1.69	1.51	1.88	1.19E-20	

						diseases of central nervous system					
rs9268652	HLA-DRA	6p21.32	Hepatitis B	A	350.3	Lack of coordination	1.28	1.19	1.38	7.45E-12	
rs9268652	HLA-DRA	6p21.32	Hepatitis B	A	557.1	Celiac disease	0.59	0.50	0.71	4.04E-09	
rs9268652	HLA-DRA	6p21.32	Hepatitis B	A	714.1	Rheumatoid arthritis	0.83	0.77	0.89	6.70E-07	Variants in HLA-DRA have been associated with rheumatoid arthritis. (PMID: 24449572)
rs9270656	HLA-DRB1, HLA-DQA1	6p21.32	Otitis media (rs4329147)	C	244.4	Hypothyroidism NOS	0.86	0.83	0.90	5.30E-10	variants in HLA-DRB1, HAL-DQA1 have been associated with hypothyroidism. (PMID: 34594039)
rs9270656	HLA-DRB1, HLA-DQA1	6p21.32	Otitis media (rs4329147)	C	245.21	Chronic lymphocytic thyroiditis	0.70	0.62	0.80	1.97E-07	
rs9270656	HLA-DRB1, HLA-DQA1	6p21.32	Otitis media (rs4329147)	C	250	Diabetes mellitus	0.69	0.62	0.78	1.07E-10	variants within HLA-DRB1, HLA-DQA1 have been associated with type 1 and 2 diabetes. (PMID: 30297969,28566273,33179336)
rs9270656	HLA-DRB1, HLA-DQA1	6p21.32	Otitis media (rs4329147)	C	250.1	Type 1 diabetes	0.58	0.53	0.63	3.29E-32	
rs9270656	HLA-DRB1, HLA-DQA1	6p21.32	Otitis media (rs4329147)	C	250.11	Type 1 diabetes with ketoacidosis	0.21	0.15	0.30	5.17E-19	
rs9270656	HLA-DRB1, HLA-DQA1	6p21.32	Otitis media (rs4329147)	C	250.12	Type 1 diabetes with renal manifestations	0.39	0.30	0.50	2.39E-13	

rs9270656	HLA-DRB1, HLA-DQA1	6p21.32	Otitis media (rs4329147)	C	250.13	Type 1 diabetes with ophthalmic manifestations	0.29	0.21	0.40	1.04E-14	
rs9270656	HLA-DRB1, HLA-DQA1	6p21.32	Otitis media (rs4329147)	C	250.14	Type 1 diabetes with neurological manifestations	0.53	0.44	0.63	5.23E-13	
rs9270656	HLA-DRB1, HLA-DQA1	6p21.32	Otitis media (rs4329147)	C	250.2	Type 2 diabetes	0.85	0.81	0.89	5.72E-14	
rs9270656	HLA-DRB1, HLA-DQA1	6p21.32	Otitis media (rs4329147)	C	250.21	Type 2 diabetes with ketoacidosis	0.52	0.40	0.67	3.66E-07	
rs9270656	HLA-DRB1, HLA-DQA1	6p21.32	Otitis media (rs4329147)	C	250.3	Insulin pump user	0.66	0.60	0.72	7.33E-22	
rs9270656	HLA-DRB1, HLA-DQA1	6p21.32	Otitis media (rs4329147)	C	250.6	Polyneuropathy in diabetes	0.77	0.70	0.85	1.08E-07	
rs9270656	HLA-DRB1, HLA-DQA1	6p21.32	Otitis media (rs4329147)	C	250.7	Diabetic retinopathy	0.67	0.58	0.77	7.03E-09	
rs9270656	HLA-DRB1, HLA-DQA1	6p21.32	Otitis media (rs4329147)	C	251.1	Hypoglycemia	0.74	0.65	0.84	6.07E-06	
rs9270656	HLA-DRB1, HLA-DQA1	6p21.32	Otitis media (rs4329147)	C	335	Multiple sclerosis	2.10	1.93	2.29	8.50E-66	variants within HLA-DRB1, HLA-DQA1 have been associated with multiple sclerosis. (PMID: 19525955)
rs9270656	HLA-DRB1, HLA-DQA1	6p21.32	Otitis media (rs4329147)	C	341	Other demyelinating	1.87	1.66	2.10	2.06E-24	

						diseases of central nervous system					
rs9270656	HLA-DRB1, HLA-DQA1	6p21.32	Otitis media (rs4329147)	C	350.2	Abnormality of gait	1.14	1.08	1.21	5.11E-06	
rs9270656	HLA-DRB1, HLA-DQA1	6p21.32	Otitis media (rs4329147)	C	350.3	Lack of coordination	1.44	1.33	1.56	3.62E-19	
rs9270656	HLA-DRB1, HLA-DQA1	6p21.32	Otitis media (rs4329147)	C	557.1	Celiac disease	0.53	0.42	0.66	1.53E-08	
rs9270656	HLA-DRB1, HLA-DQA1	6p21.32	Otitis media (rs4329147)	C	714.1	Rheumatoid arthritis	0.79	0.72	0.87	4.19E-07	variants within HLA-DRB1, HLA-DQA1 have been associated with rheumatoid arthritis. (PMID: 34594039, 24532677, 23143596)
rs73027818	PLG	6q26	Otitis media	C	696	Psoriasis and related disorders	0.14	0.06	0.34	1.36E-05	
rs600038	ABO	9q34.2	Otitis media	T	112	Candidiasis	1.14	1.07	1.21	2.35E-05	
rs600038	ABO	9q34.2	Otitis media	T	286.12	Congenital deficiency of other clotting factors (including factor VII)	0.49	0.38	0.64	4.41E-08	rs600038 has been associated with heart failure. (PMID: 31919418, 34594039)
rs600038	ABO	9q34.2	Otitis media	T	286.2	Encounter for long-term (current) use of anticoagulants	0.85	0.80	0.89	4.28E-10	
rs600038	ABO	9q34.2	Otitis media	T	286.81	Primary hypercoagulable	0.65	0.58	0.74	2.17E-12	

						state hematopoietic					
rs600038	ABO	9q34.2	Otitis media	T	415	Pulmonary heart disease	0.78	0.71	0.86	5.12E-07	
rs600038	ABO	9q34.2	Otitis media	T	415.11	Pulmonary embolism and infarction, acute circulatory	0.76	0.70	0.82	2.17E-12	
rs600038	ABO	9q34.2	Otitis media	T	451.2	Phlebitis and thrombophlebitis of lower extremities	0.74	0.65	0.85	2.48E-05	Variants within ABO have been associated with venous thromboembolism. (PMID: 30659681,25772935, 21980494, 19278955, 22672568)
rs600038	ABO	9q34.2	Otitis media	T	452	Other venous embolism and thrombosis	0.82	0.77	0.86	5.38E-14	
rs600038	ABO	9q34.2	Otitis media	T	452.2	Deep vein thrombosis [DVT]	0.77	0.72	0.82	5.54E-16	
rs681343	FUT2	19q13.3	Otitis media	C	261.2	Vitamin B-complex deficiencies	1.17	1.10	1.23	8.63E-08	variants within FUT2 have been associated with vitamin B levels. (PMID: 18776911,19744961,19303062, 28334792)
rs1978060	TBX1	22q11.2	Otitis media	A	245.21	Chronic lymphocytic thyroiditis	1.21	1.10	1.31	2.69E-05	

Supplementary Table 4. Associations between genetically predicted gene expression and common infections (p<10E-5).

UTI								
tissue	gene	gene_name	zscore	effect_size	pvalue	var_g	n_snps_used	n_snps_
Adrenal_Gland	ENSG00000106113.18	CRHR2	-4.68409	-0.38391	2.81E-06	0.020811	1	1
Colon_Transverse	ENSG00000106113.18	CRHR2	-4.68409	-0.43271	2.81E-06	0.016683	1	2
Esophagus_Gastroesophageal_Junction	ENSG00000106113.18	CRHR2	-4.68409	-0.3722	2.81E-06	0.025608	1	2
Heart_Left_Ventricle	ENSG00000132669.12	RIN2	-4.69822	-0.95774	2.62E-06	0.003368	1	1
chronic_sinus_infection								
tissue	gene	gene_name	zscore	effect_size	pvalue	var_g	n_snps_used	n_snps_
Skin_Sun_Exposed_Lower_leg	ENSG00000140948.11	ZCCHC14	-4.56265	-1.16201	5.05E-06	0.007082	1	2
Testis	ENSG00000140948.11	ZCCHC14	-4.51979	-1.09635	6.19E-06	0.008323	1	2
childhood_ear_infection								
tissue	gene	gene_name	zscore	effect_size	pvalue	var_g	n_snps_used	n_snps_
Brain_Amygdala	ENSG00000014824.13	SLC30A9	4.656591	0.923067	3.21E-06	0.021219	3	3
Brain_Anterior_cingulate_cortex_BA24	ENSG00000014824.13	SLC30A9	4.616235	1.423244	3.91E-06	0.008456	2	2
Brain_Hippocampus	ENSG00000014824.13	SLC30A9	4.586956	1.130658	4.5E-06	0.014015	3	3
Brain_Nucleus_accumbens_basal_ganglia	ENSG00000014824.13	SLC30A9	4.933922	1.029812	8.06E-07	0.014934	2	2
Pancreas	ENSG00000224397.5	SMIM25	-4.43797	-0.43518	9.08E-06	0.062722	4	4
Testis	ENSG00000168269.8	FOXI1	4.517515	0.330174	6.26E-06	0.096741	1	1
yeast_infection								

tissue	gene	gene_name	zscore	effect_size	pvalue	var_g	n_snps_used	n_snps_
Adrenal_Gland	ENSG00000130881.13	LRP3	5.073193	2.332661	3.91E-07	0.003467	1	1
Artery_Coronary	ENSG00000130881.13	LRP3	5.134841	2.667267	2.82E-07	0.0029	2	2
Brain_Cortex	ENSG00000166359.10	WDR88	4.758537	0.392814	1.95E-06	0.087545	1	1
Brain_Spinal_cord_cervical_c-1	ENSG00000130881.13	LRP3	5.170222	6.49855	2.34E-07	0.000383	2	2
Esophagus_Mucosa	ENSG00000130881.13	LRP3	5.680319	2.126066	1.34E-08	0.005041	2	2
Liver	ENSG00000166359.10	WDR88	5.535248	0.548602	3.11E-08	0.060999	1	1
Minor_Salivary_Gland	ENSG00000130881.13	LRP3	5.073193	2.363088	3.91E-07	0.003164	1	1
Prostate	ENSG00000130881.13	LRP3	5.073193	3.394779	3.91E-07	0.001917	1	1
Skin_Sun_Exposed_Lower_leg	ENSG00000170191.4	NANP	-4.50925	-0.74099	6.51E-06	0.021215	2	2
Spleen	ENSG00000130881.13	LRP3	5.090514	2.03693	3.57E-07	0.004629	2	2
hepatitisC								
tissue	gene	gene_name	zscore	effect_size	pvalue	var_g	n_snps_used	n_snps_
Brain_Cortex	ENSG00000188338.14	SLC38A3	-4.49912	-52.4181	6.82E-06	7.95E-06	1	2
Brain_Nucleus_accumbens_basal_ganglia	ENSG00000054356.13	PTPRN	4.492813	0.666677	7.03E-06	0.034922	2	2
strep_throat								
tissue	gene	gene_name	zscore	effect_size	pvalue	var_g	n_snps_used	n_snps_
Adipose_Subcutaneous	ENSG00000196505.10	GDAP2	4.6461	4.296129	3.38E-06	0.002578	2	2
Cells_Cultured_fibroblasts	ENSG00000112167.9	SAYSD1	4.651023	1.372833	3.3E-06	0.012694	2	2

Nerve_Tibial	ENSG00000196505.10	GDAP2	4.678264	3.662003	2.89E-06	0.003461	3	3
Thyroid	ENSG00000196505.10	GDAP2	4.676107	3.099468	2.92E-06	0.004247	2	2
shingles								
tissue	gene	gene_name	zscore	effect_size	pvalue	var_g	n_snps_used	n_snps_[illegible]
Brain_Frontal_Cortex_BA9	ENSG00000197406.7	DIO3	-4.47725	-10.2009	7.56E-06	0.000223	1	1
coldsores								
tissue	gene	gene_name	zscore	effect_size	pvalue	var_g	n_snps_used	n_snps_[illegible]
Whole_Blood	ENSG00000153208.16	MERTK	4.470918	1.383602	7.79E-06	0.032734	2	2
hepatitisB								
tissue	gene	gene_name	zscore	effect_size	pvalue	var_g	n_snps_used	n_snps_[illegible]
Adipose_Visceral_Omentum	ENSG00000165312.6	OTUD1	-4.47136	-165.698	7.77E-06	6.4E-06	1	3
Brain_Amygdala	ENSG00000165312.6	OTUD1	-4.47136	-67.0356	7.77E-06	1.87E-05	1	2
Brain_Caudate_basal_ganglia	ENSG00000165312.6	OTUD1	-4.47136	-86.2198	7.77E-06	2.32E-05	1	2
Brain_Caudate_basal_ganglia	ENSG00000087884.14	AAMDC	-4.43142	-2.09463	9.36E-06	0.026704	2	2
Brain_Cerebellar_Hemisphere	ENSG00000165312.6	OTUD1	-4.47136	-224.811	7.77E-06	2.51E-06	1	2
Brain_Hippocampus	ENSG00000165312.6	OTUD1	-4.47136	-480.509	7.77E-06	8.56E-07	1	1
Brain_Putamen_basal_ganglia	ENSG00000165312.6	OTUD1	-4.47136	-46.1975	7.77E-06	6.1E-05	1	1
Brain_Substantia_nigra	ENSG00000165312.6	OTUD1	-4.47136	-69.2969	7.77E-06	2.09E-05	1	2
Breast_Mammary_Tissue	ENSG00000087884.14	AAMDC	-4.44447	-2.59061	8.81E-06	0.014265	2	2

Cells_Cultured_fibroblasts	ENSG00000165312.6	OTUD1	-4.47136	-544.561	7.77E-06	4.97E-07	1	2
Colon_Transverse	ENSG00000165312.6	OTUD1	-4.47136	-1227.08	7.77E-06	1.33E-07	1	2
Heart_Atrial_Appendage	ENSG00000087884.14	AAMDC	-4.80938	-2.58799	1.51E-06	0.019472	3	3
Heart_Atrial_Appendage	ENSG00000165312.6	OTUD1	4.471356	278.2932	7.77E-06	2.42E-06	1	2
Heart_Left_Ventricle	ENSG00000087884.14	AAMDC	-4.62229	-3.14159	3.8E-06	0.013152	3	3
Heart_Left_Ventricle	ENSG00000165312.6	OTUD1	-4.47136	-3657.13	7.77E-06	1.57E-08	1	2
Lung	ENSG00000165312.6	OTUD1	-4.47136	-230.925	7.77E-06	3.81E-06	1	2
Minor_Salivary_Gland	ENSG00000165312.6	OTUD1	4.471356	1461.076	7.77E-06	8.16E-08	1	1
Ovary	ENSG00000165312.6	OTUD1	-4.47136	-79.2295	7.77E-06	3.42E-05	1	2
Pancreas	ENSG00000165312.6	OTUD1	-4.47136	-113.335	7.77E-06	1.27E-05	1	2
Prostate	ENSG00000087884.14	AAMDC	-4.62531	-2.77616	3.74E-06	0.016749	2	2
Prostate	ENSG00000165312.6	OTUD1	-4.47136	-273.753	7.77E-06	1.47E-06	1	2
Skin_Not_Sun_Exposed_Suprapubic	ENSG00000087884.14	AAMDC	-4.88725	-3.35086	1.02E-06	0.015421	3	3
Stomach	ENSG00000276644.4	DACH1	4.700371	198.1288	2.6E-06	4.17E-06	1	1
Thyroid	ENSG00000276644.4	DACH1	-4.70037	-164.46	2.6E-06	5.69E-06	1	1
Uterus	ENSG00000165312.6	OTUD1	-4.47136	-326.599	7.77E-06	1.74E-06	1	1
Vagina	ENSG00000165312.6	OTUD1	-4.47136	-820.054	7.77E-06	3.66E-07	1	2

Whole_Blood	ENSG00000165312.6	OTUD1	-4.47136	-29.3828	7.77E-06	0.000245	1	2
mononucleosis								
tissue	gene	gene_name	zscore	effect_size	pvalue	var_g	n_snps_used	n_snps_
Adipose_Subcutaneous	ENSG00000229656.6	RP11-462L8.1	4.49438	22.99418	6.98E-06	0.000433	1	1
Adipose_Visceral_Omentum	ENSG00000179344.16	HLA-DQB1	4.701073	1.090348	2.59E-06	0.190738	1	3
Adipose_Visceral_Omentum	ENSG00000244731.7	C4A	4.497789	6.02939	6.87E-06	0.007252	2	4
Artery_Coronary	ENSG00000150093.18	ITGB1	-4.49438	-372.33	6.98E-06	1.66E-06	1	1
Artery_Tibial	ENSG00000133422.12	MORC2	4.467973	4.93058	7.9E-06	0.008518	2	2
Heart_Left_Ventricle	ENSG00000229656.6	RP11-462L8.1	4.49438	7.766285	6.98E-06	0.004046	1	1
Heart_Left_Ventricle	ENSG00000150093.18	ITGB1	-4.49438	-213.34	6.98E-06	5.36E-06	1	2
Minor_Salivary_Gland	ENSG00000150093.18	ITGB1	-4.58964	-208.641	4.44E-06	5.45E-06	2	2
Muscle_Skeletal	ENSG00000229656.6	RP11-462L8.1	4.49438	8.486562	6.98E-06	0.003163	1	1
Spleen	ENSG00000150093.18	ITGB1	-4.49438	-75.1629	6.98E-06	4.23E-05	1	1
positive_TB								
tissue	gene	gene_name	zscore	effect_size	pvalue	var_g	n_snps_used	n_snps_
Heart_Left_Ventricle	ENSG00000260484.1	RP11-1081M5.2	-4.41862	-18.1527	9.93E-06	0.000802	2	2
Thyroid	ENSG00000140470.13	ADAMTS17	-4.49724	-1.84346	6.88E-06	0.070979	4	4

Supplementary Table 5. PheWAS of genetic variants that were associated with common infections in BioVU (suggestive p-value cutoff, 0.001)

PheCode	snp	effect allele	OR	95% CI		p	n_total	n_cases	n_controls	allele_freq	description	group
359.2	rs113235453	A	0.77	0.71	0.89	0.000623	58791	1386	57405	0.95	Myopathy	neurological
588.2	rs113235453	A	0.69	0.62	0.84	0.000295	47907	670	47237	0.95	Secondary hyperparathyroidism (of renal origin)	genitourinary
598	rs113235453	A	0.43	0.35	0.64	5.20E-05	65030	110	64920	0.95	Abnormal findings on examinatiion of urine	genitourinary
687.1	rs113235453	A	0.87	0.84	0.94	0.000714	55843	6425	49418	0.95	Rash and other nonspecific skineruption	dermatologic
70.9	rs10422015	G	0.81	0.76	0.91	0.000753	51768	843	50925	0.85	Hepatitis NOS	infectious diseases
112	rs10422015	G	0.90	0.87	0.95	0.000718	58709	3646	55063	0.85	Candidiasis	infectious diseases
313.1	rs10422015	G	1.20	1.14	1.33	0.00051	61804	1762	60042	0.85	Attention deficit hyperactivity disorder	Mental disorders
526.1	rs10422015	G	0.48	0.40	0.71	0.000224	59239	63	59176	0.84	Cysts of the jaws	digestive
