# Supplementary material for: Genome-wide association analyses of common infections in a large practice-based biobank
Source: BMC Genomics. 2022 Sep 27;23:672. doi: 10.1186/s12864-022-08888-9 (PMC9512962; doi:10.1186/s12864-022-08888-9)
Supplement: Supplementary file 3 — Additional file 3: Supplementary Figure 1. Manhattan plots of Phenome-wide associations studies. [file 12864_2022_8888_MOESM3_ESM.pdf]

Additional file 3: Supplementary Figure 1. Manhattan plots of Phenome-wide associations studies.

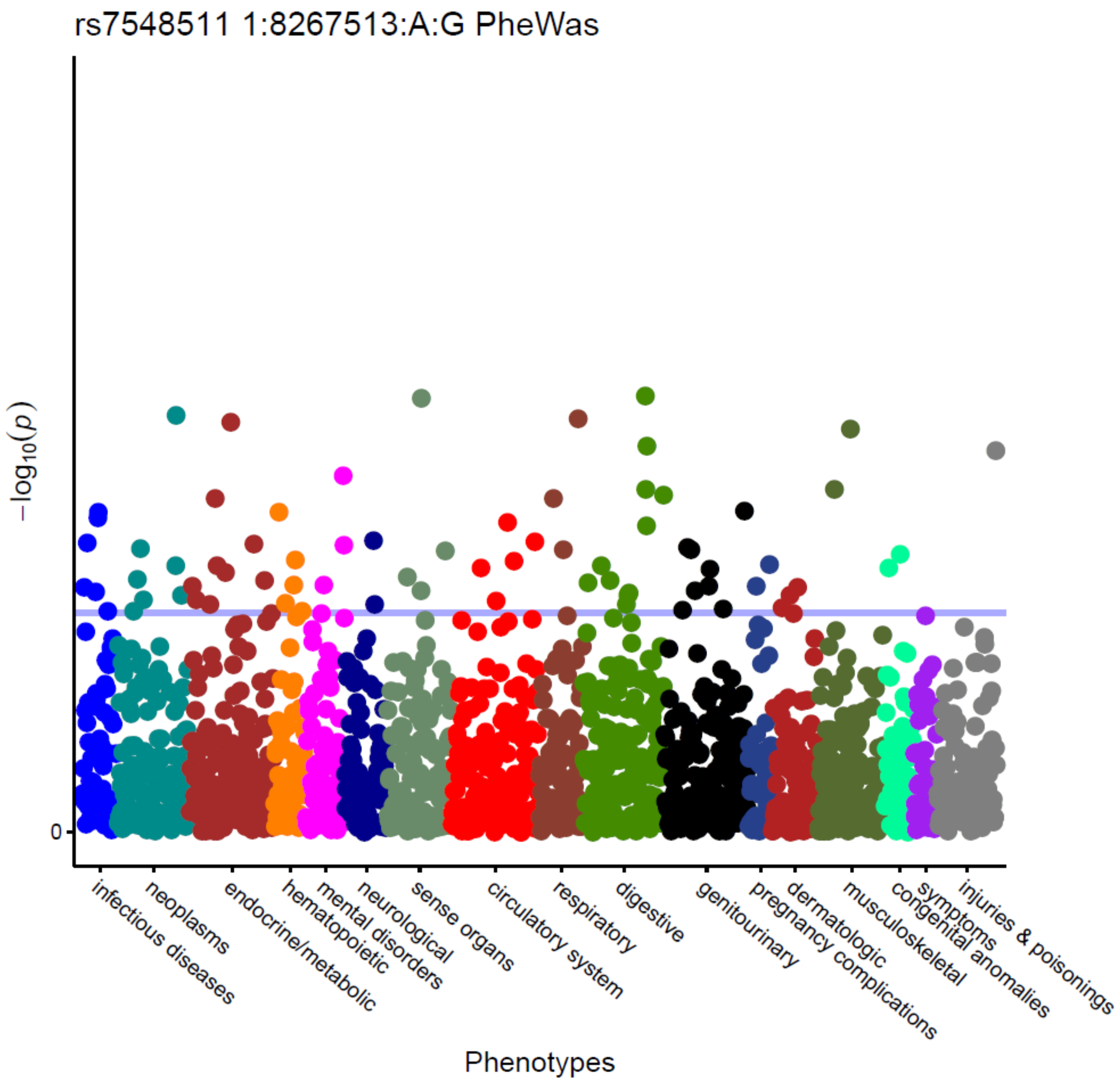

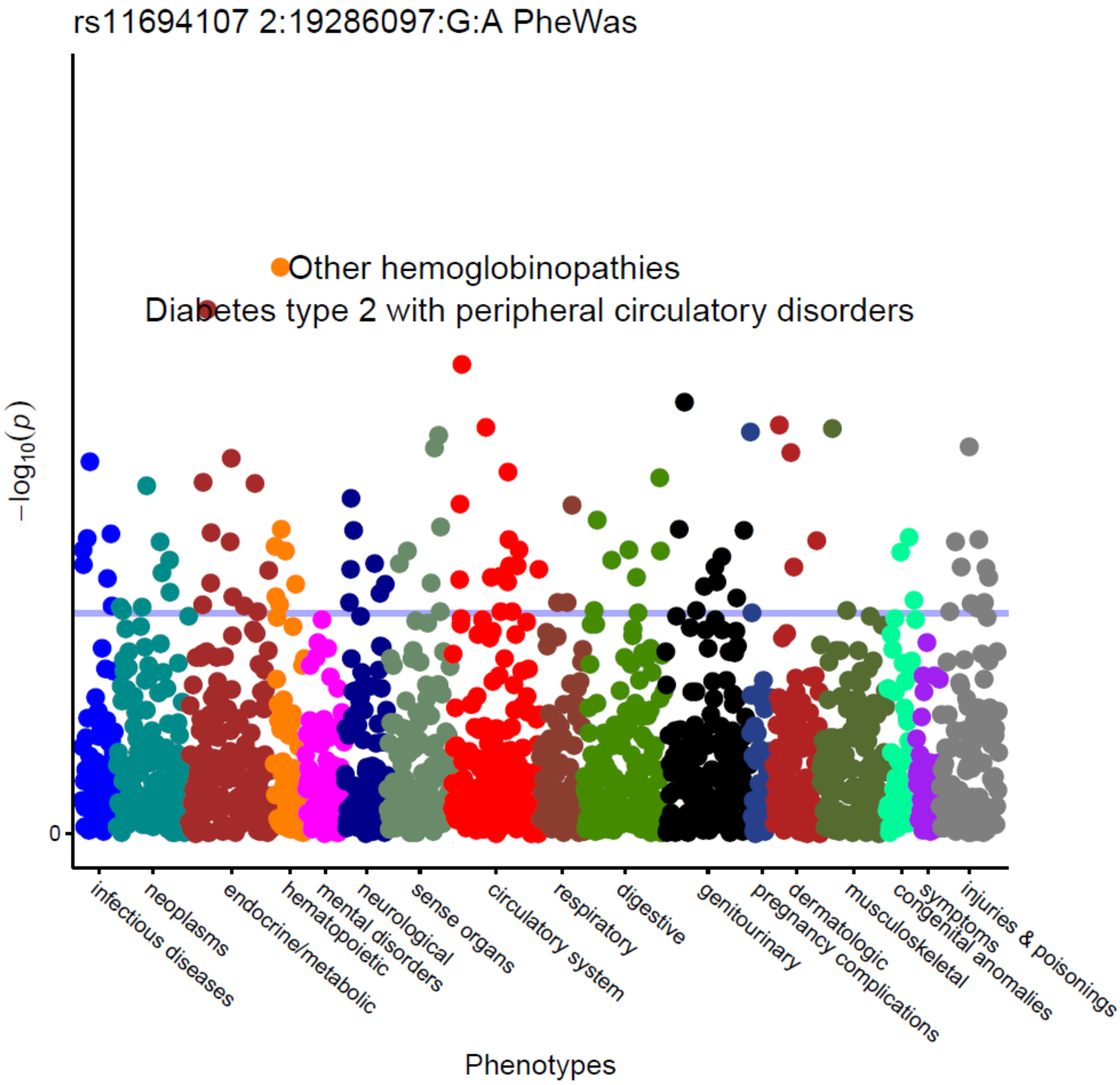
rs11694107 2:19286097:G:A PheWas
Other hemoglobinopathies
Diabetes type 2 with peripheral circulatory disorders
$-\log_{10}(p)$
0
infectious diseases
neoplasms
endocrine/metabolic
hematopoietic
mental disorders
neurological
sense organs
circulatory system
respiratory
digestive
genitourinary
pregnancy complications
dermatologic
musculoskeletal
congenital anomalies
symptoms
injuries & poisonings
Phenotypes

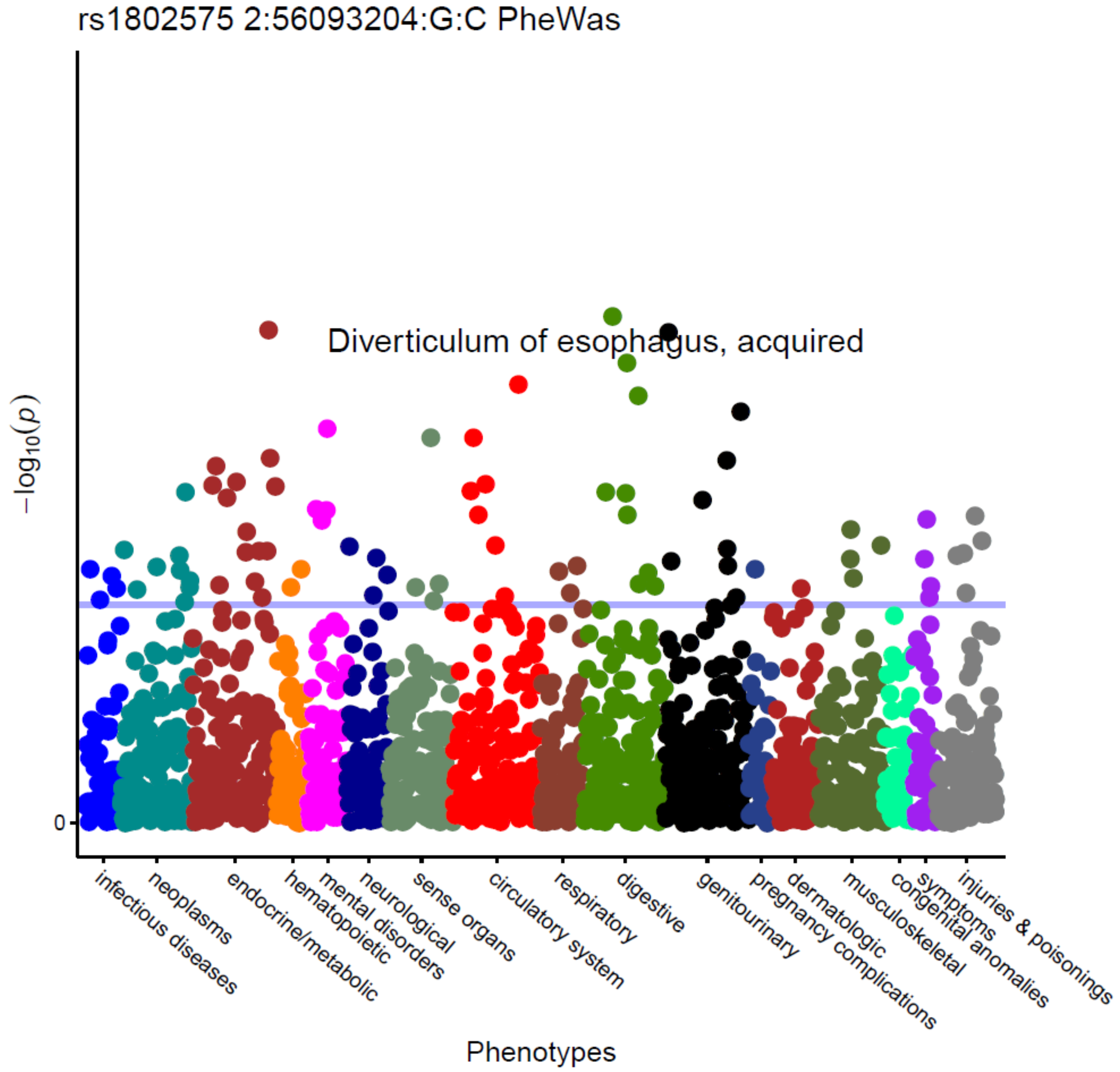
rs1802575 2:56093204:G:C PheWas
Diverticulum of esophagus, acquired
$-\log_{10}(p)$
0
infectious diseases
neoplasms
endocrine/metabolic
hematopoietic
mental disorders
neurological
sense organs
circulatory system
respiratory
digestive
genitourinary
pregnancy complications
dermatologic
musculoskeletal
congenital anomalies
symptoms
injuries & poisonings
Phenotypes

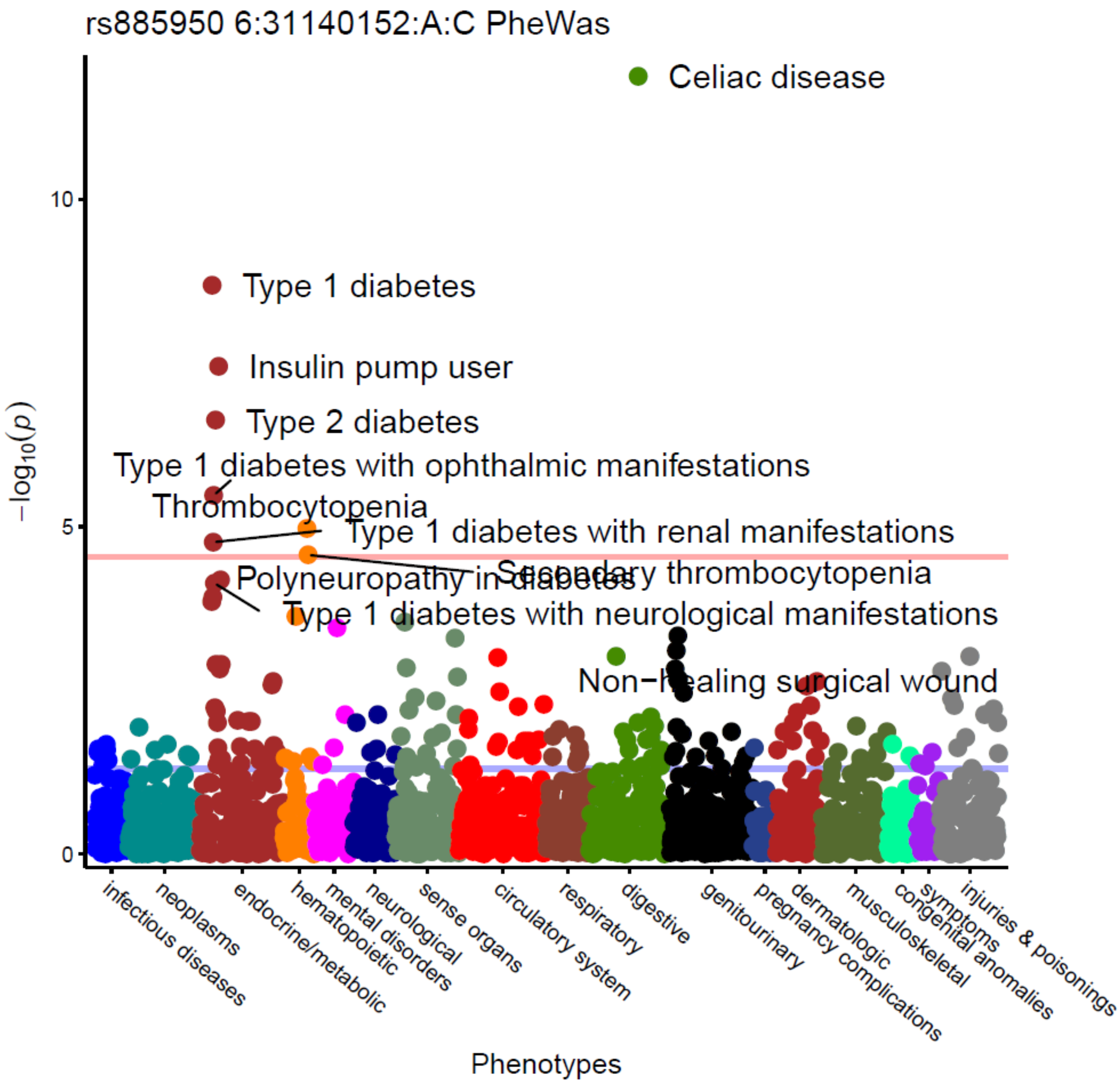
rs885950 6:31140152:A:C PheWas
Celiac disease
Type 1 diabetes
Insulin pump user
Type 2 diabetes
Type 1 diabetes with ophthalmic manifestations
Thrombocytopenia
Type 1 diabetes with renal manifestations
Secondary thrombocytopenia
Polyneuropathy in diabetes
Type 1 diabetes with neurological manifestations
Non−healing surgical wound
$-\log_{10}(p)$
10
5
0
infectious diseases
neoplasms
endocrine/metabolic
hematopoietic
mental disorders
neurological
sense organs
circulatory system
respiratory
digestive
genitourinary
pregnancy complications
dermatologic
musculoskeletal
congenital anomalies
symptoms
injuries & poisonings
Phenotypes

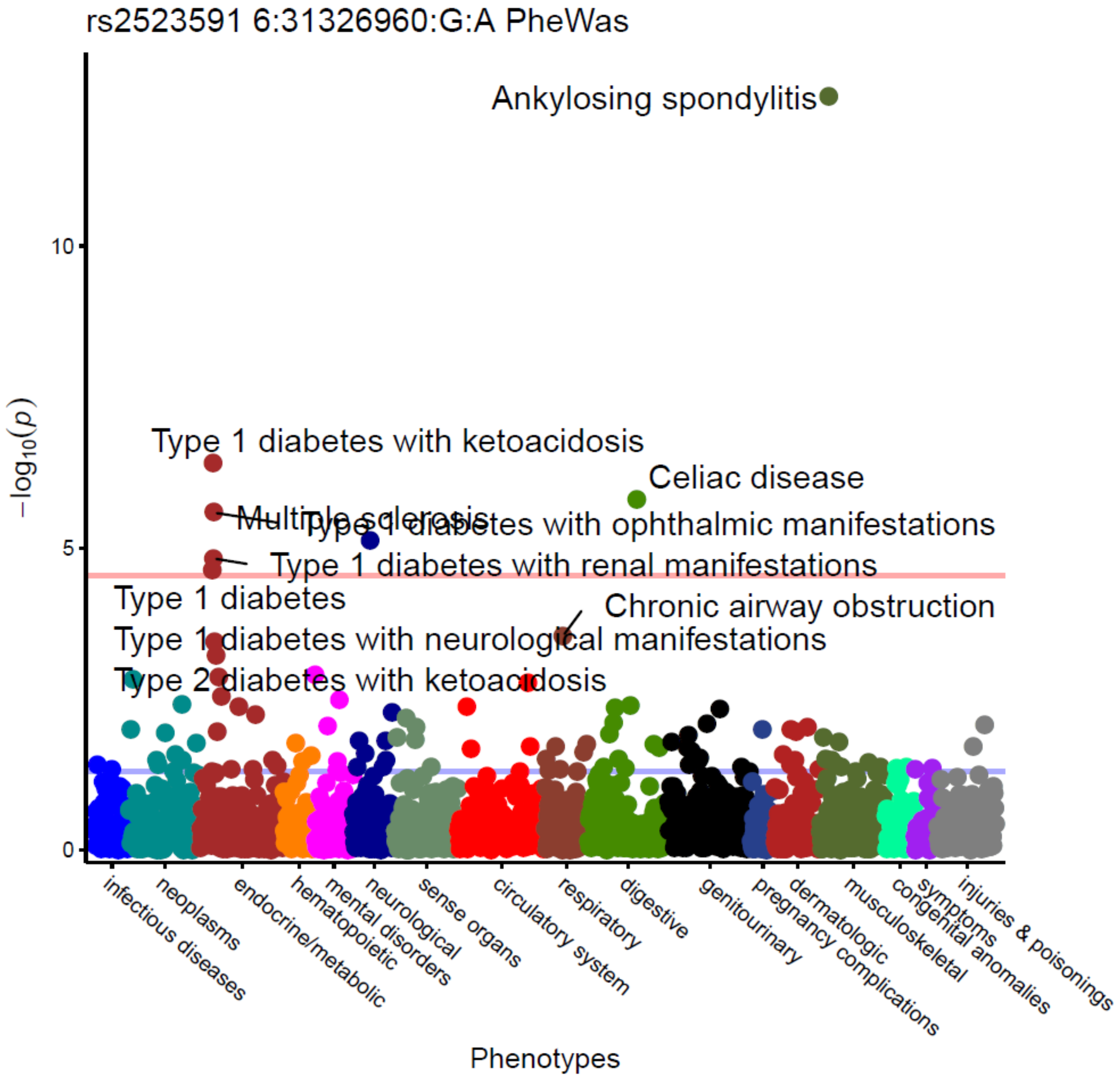

rs2523591 6:31326960:G:A PheWas
Ankylosing spondylitis
Type 1 diabetes with ketoacidosis
Celiac disease
Multiple sclerosis
Type 1 diabetes with ophthalmic manifestations
Type 1 diabetes with renal manifestations
Type 1 diabetes
Chronic airway obstruction
Type 1 diabetes with neurological manifestations
Type 2 diabetes with ketoacidosis
−log10(p)
10
5
0
infectious diseases
neoplasms
endocrine/metabolic
hematopoietic
mental disorders
neurological
sense organs
circulatory system
respiratory
digestive
genitourinary
pregnancy complications
dermatologic
musculoskeletal
congenital anomalies
symptoms
injuries & poisonings
Phenotypes

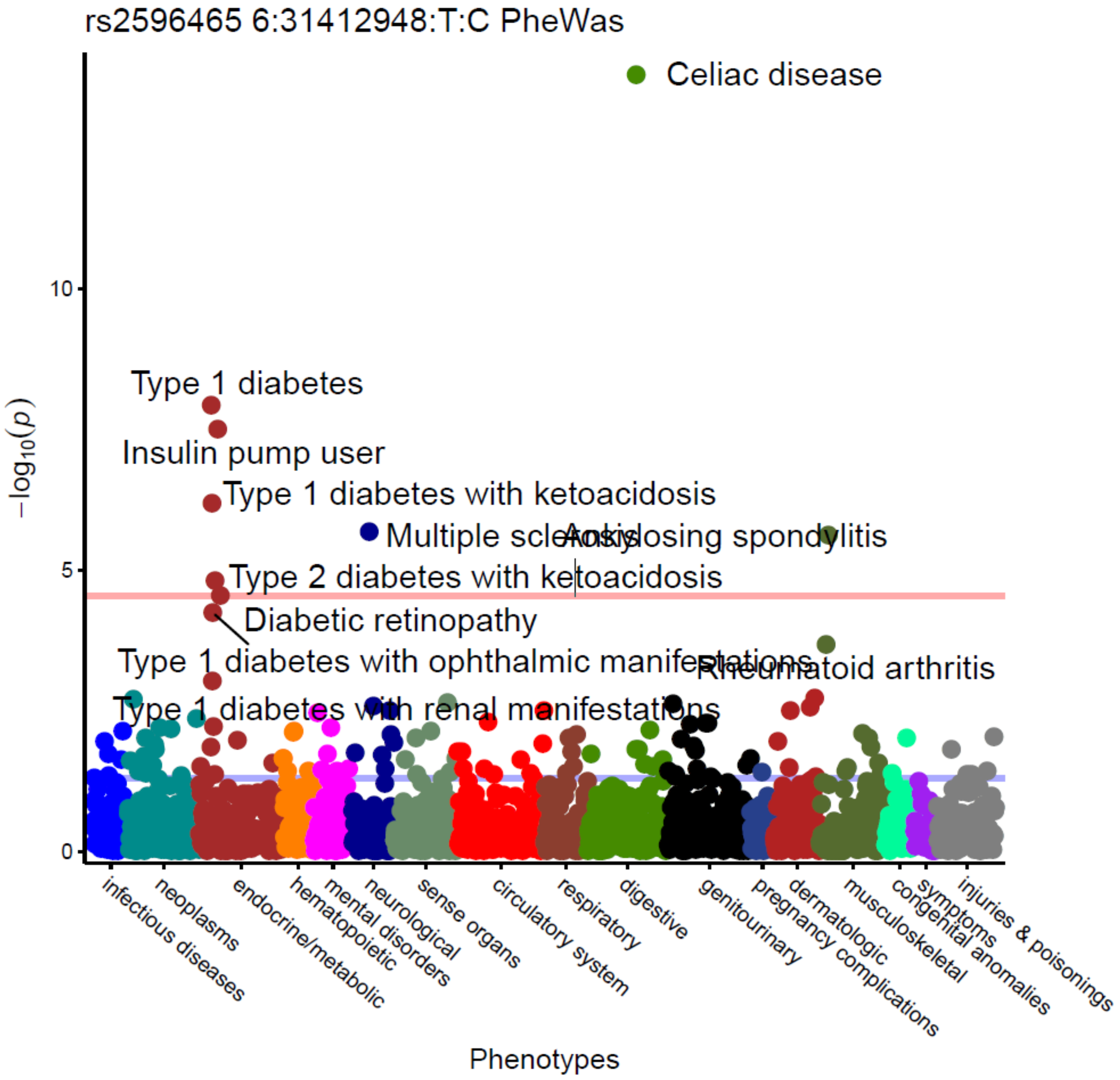
rs2596465 6:31412948:T:C PheWas
Celiac disease
Type 1 diabetes
Insulin pump user
Type 1 diabetes with ketoacidosis
Multiple sclerosis
Ankylosing spondylitis
Type 2 diabetes with ketoacidosis
Diabetic retinopathy
Type 1 diabetes with ophthalmic manifestations
Rheumatoid arthritis
Type 1 diabetes with renal manifestations
$-\log_{10}(p)$
0
5
10
infectious diseases
neoplasms
endocrine/metabolic
hematopoietic
mental disorders
neurological
sense organs
circulatory system
respiratory
digestive
genitourinary
pregnancy complications
dermatologic
musculoskeletal
congenital anomalies
symptoms
injuries & poisonings
Phenotypes

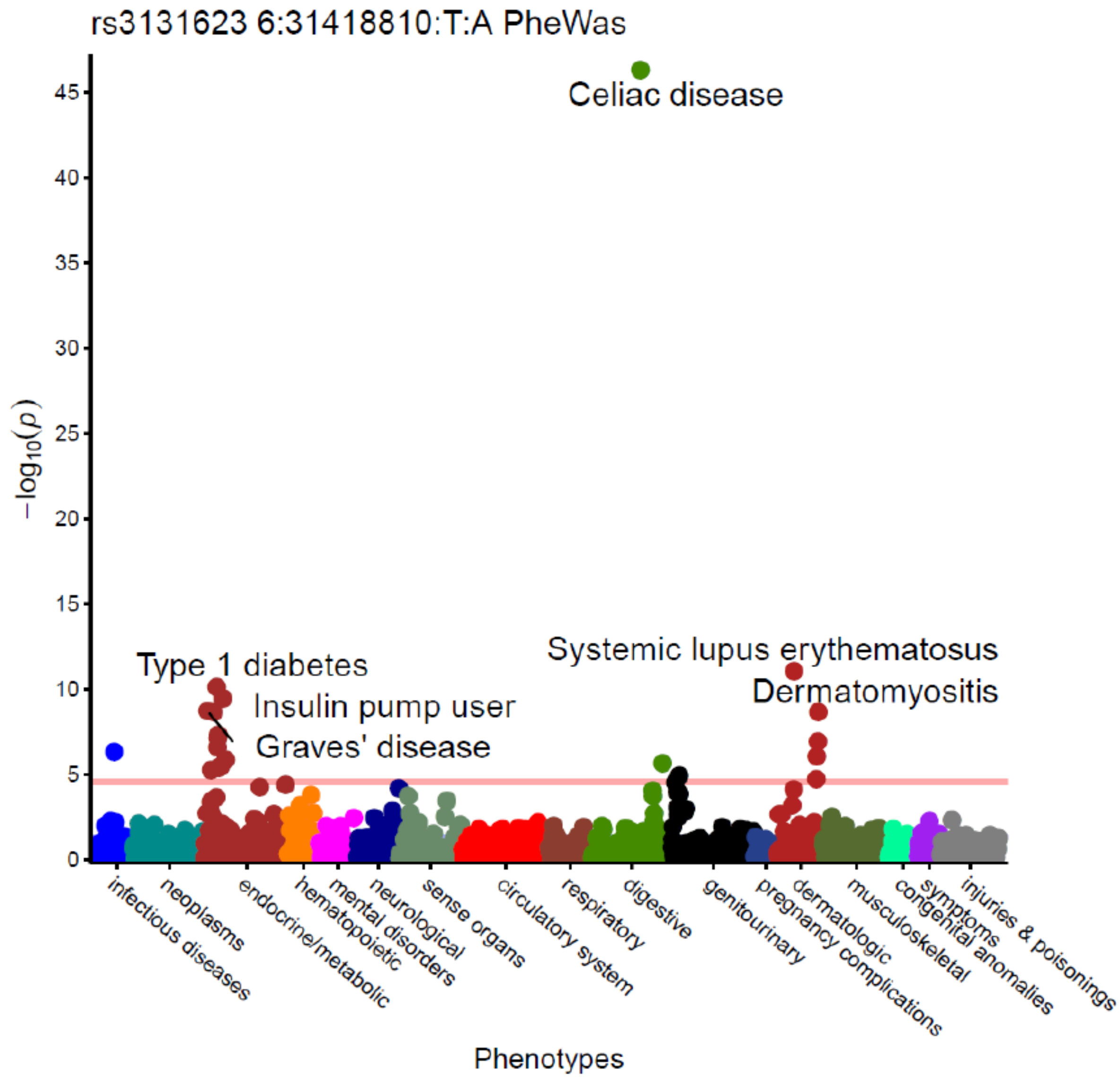

rs3131623 6:31418810:T:A PheWas
Celiac disease
Type 1 diabetes
Insulin pump user
Graves' disease
Systemic lupus erythematosus
Dermatomyositis
$-\log_{10}(p)$
0
5
10
15
20
25
30
35
40
45
infectious diseases
neoplasms
endocrine/metabolic
hematopoietic
mental disorders
neurological
sense organs
circulatory system
respiratory
digestive
genitourinary
pregnancy complications
dermatologic
musculoskeletal
congenital anomalies
symptoms
injuries & poisonings
Phenotypes

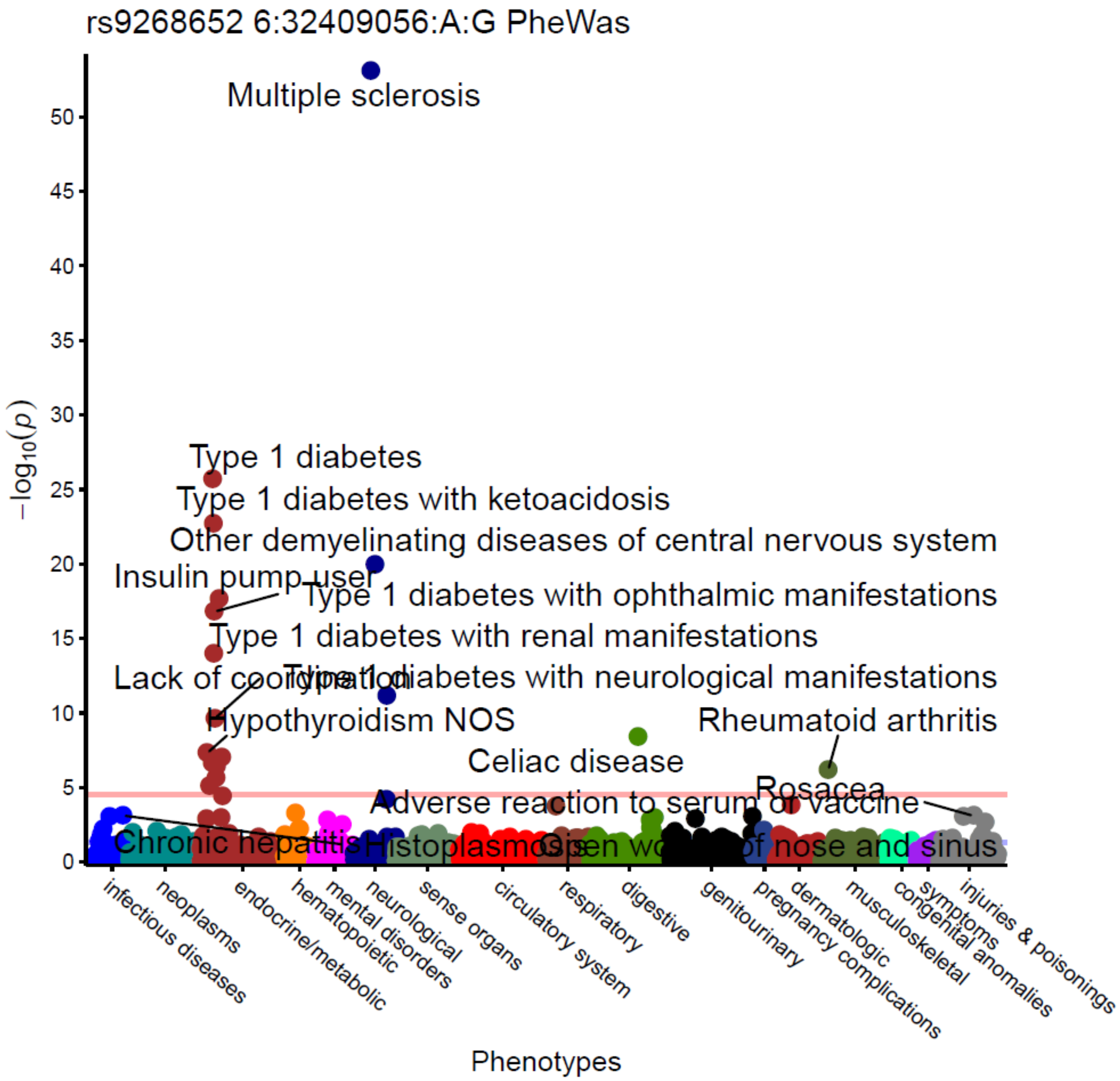
rs9268652 6:32409056:A:G PheWas
Multiple sclerosis
Type 1 diabetes
Type 1 diabetes with ketoacidosis
Other demyelinating diseases of central nervous system
Insulin pump user
Type 1 diabetes with ophthalmic manifestations
Type 1 diabetes with renal manifestations
Type 1 diabetes with neurological manifestations
Hypothyroidism NOS
Rheumatoid arthritis
Celiac disease
Rosacea
Adverse reaction to serum or vaccine
Chronic hepatitis
nose and sinus
−log10(p)
0
5
10
15
20
25
30
35
40
45
50
Phenotypes
infectious diseases
neoplasms
endocrine/metabolic
hematopoietic
mental disorders
neurological
sense organs
circulatory system
respiratory
digestive
genitourinary
pregnancy complications
dermatologic
musculoskeletal
congenital anomalies
symptoms
injuries & poisonings

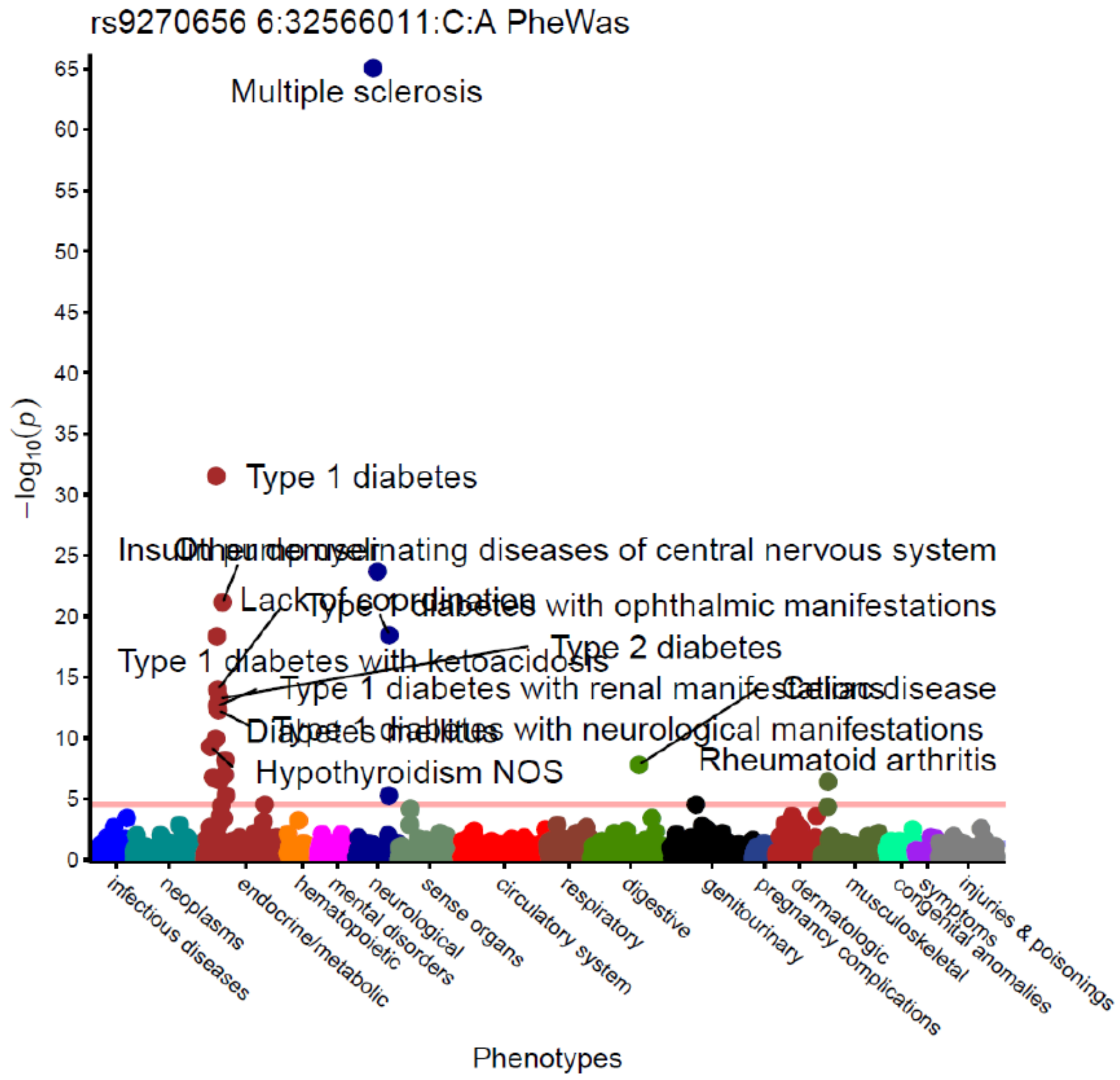
rs9270656 6:32566011:C:A PheWas
Multiple sclerosis
Type 1 diabetes
Type 2 diabetes
Rheumatoid arthritis
Hypothyroidism NOS
Type 1 diabetes with ketoacidosis
$-\log_{10}(p)$
0
5
10
15
20
25
30
35
40
45
50
55
60
65
infectious diseases
neoplasms
endocrine/metabolic
hematopoietic
mental disorders
neurological
sense organs
circulatory system
respiratory
digestive
genitourinary
pregnancy complications
dermatologic
musculoskeletal
congenital anomalies
symptoms
injuries & poisonings
Phenotypes

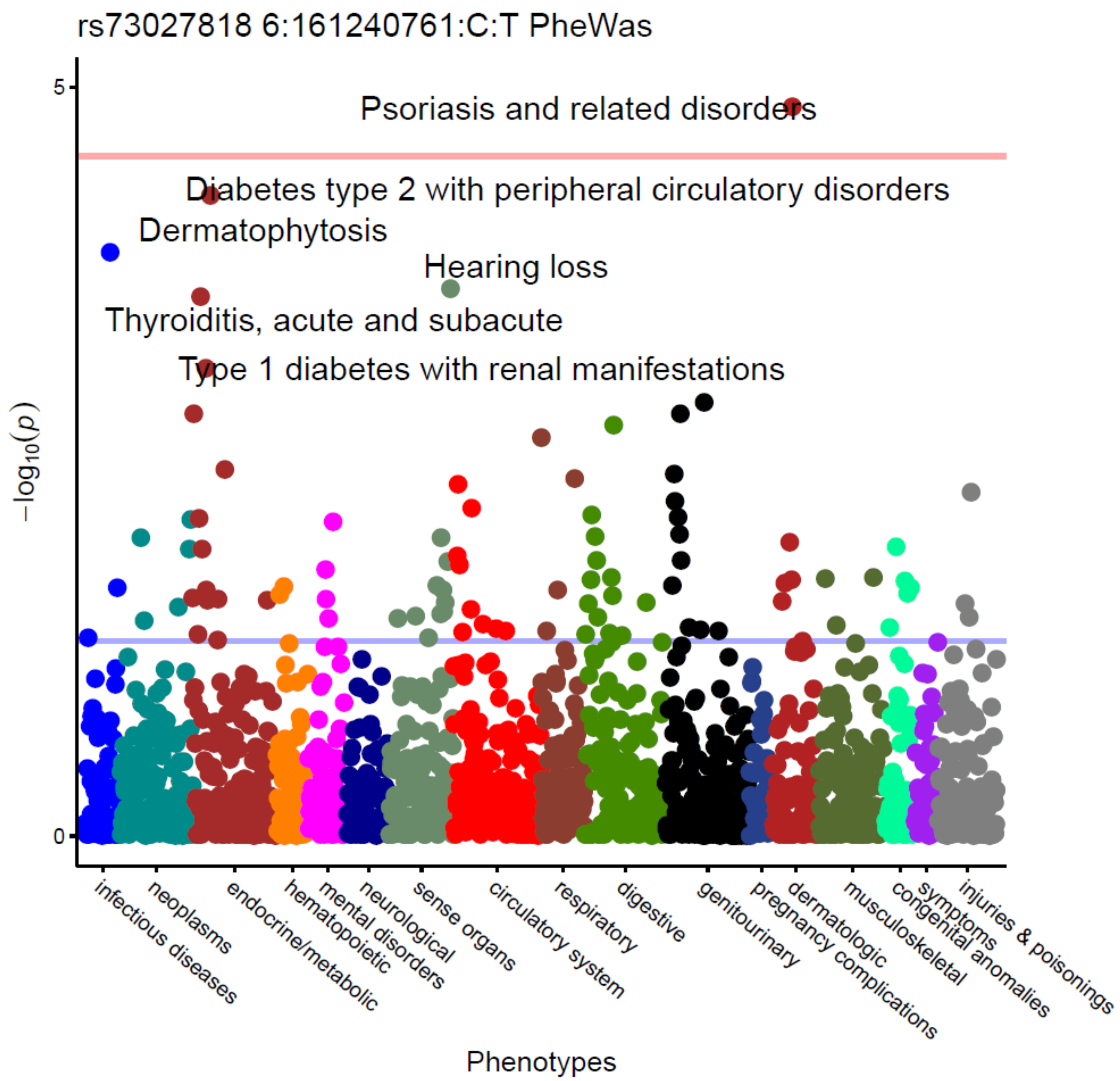
rs73027818 6:161240761:C:T PheWas
Psoriasis and related disorders
Diabetes type 2 with peripheral circulatory disorders
Dermatophytosis
Hearing loss
Thyroiditis, acute and subacute
Type 1 diabetes with renal manifestations
5
0
−log10(p)
infectious diseases
neoplasms
endocrine/metabolic
hematopoietic
mental disorders
neurological
sense organs
circulatory system
respiratory
digestive
genitourinary
pregnancy complications
dermatologic
musculoskeletal
congenital anomalies
symptoms
injuries & poisonings
Phenotypes

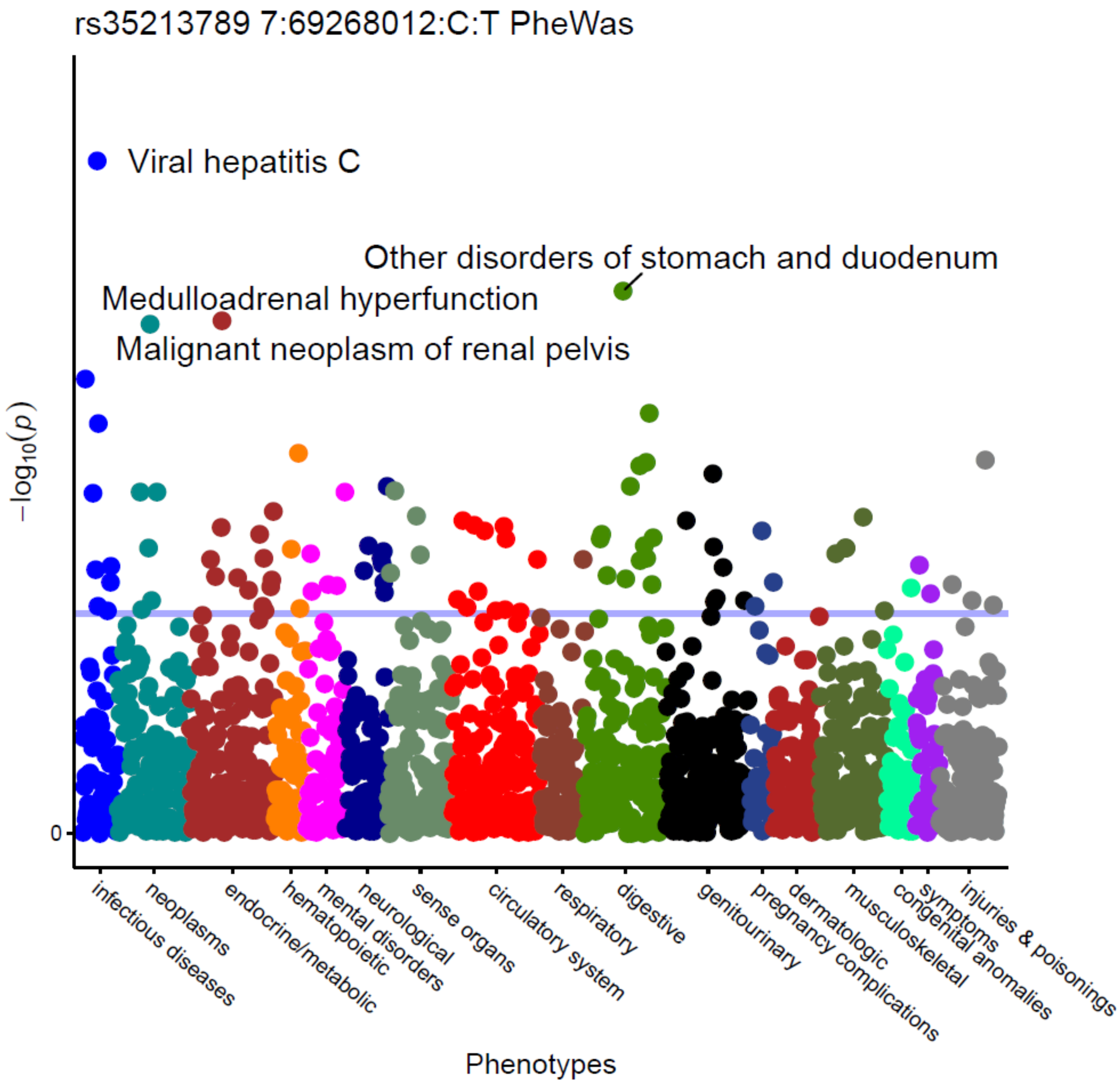
rs35213789 7:69268012:C:T PheWas
Viral hepatitis C
Other disorders of stomach and duodenum
Medulloadrenal hyperfunction
Malignant neoplasm of renal pelvis
−log10(p)
0
infectious diseases
neoplasms
endocrine/metabolic
hematopoietic
mental disorders
neurological
sense organs
circulatory system
respiratory
digestive
genitourinary
pregnancy complications
dermatologic
musculoskeletal
congenital anomalies
symptoms
injuries & poisonings
Phenotypes

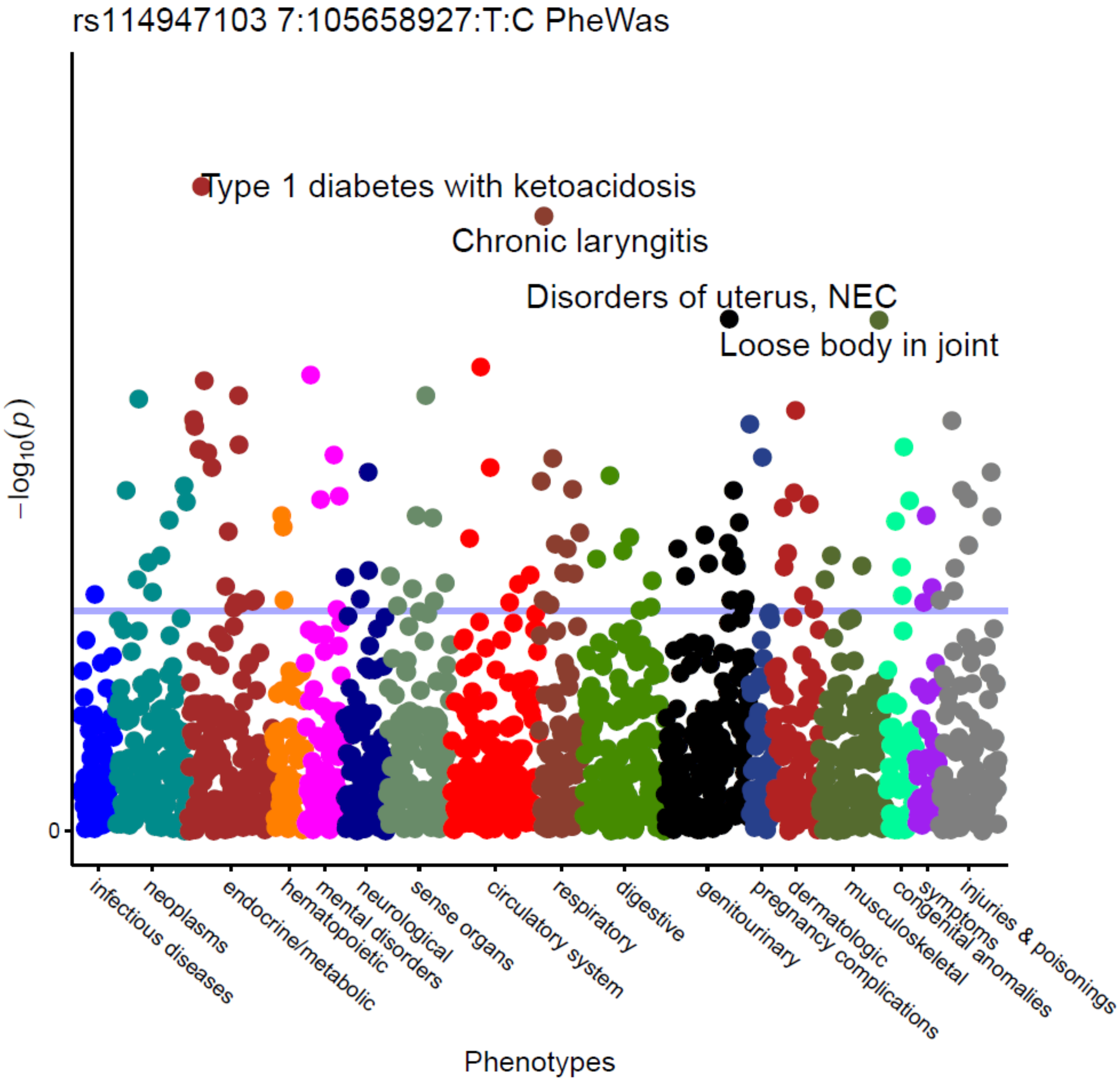
rs114947103 7:105658927:T:C PheWas
Type 1 diabetes with ketoacidosis
Chronic laryngitis
Disorders of uterus, NEC
Loose body in joint
$-\log_{10}(p)$
0
infectious diseases
neoplasms
endocrine/metabolic
hematopoietic
mental disorders
neurological
sense organs
circulatory system
respiratory
digestive
genitourinary
pregnancy complications
dermatologic
musculoskeletal
congenital anomalies
symptoms
injuries & poisonings
Phenotypes

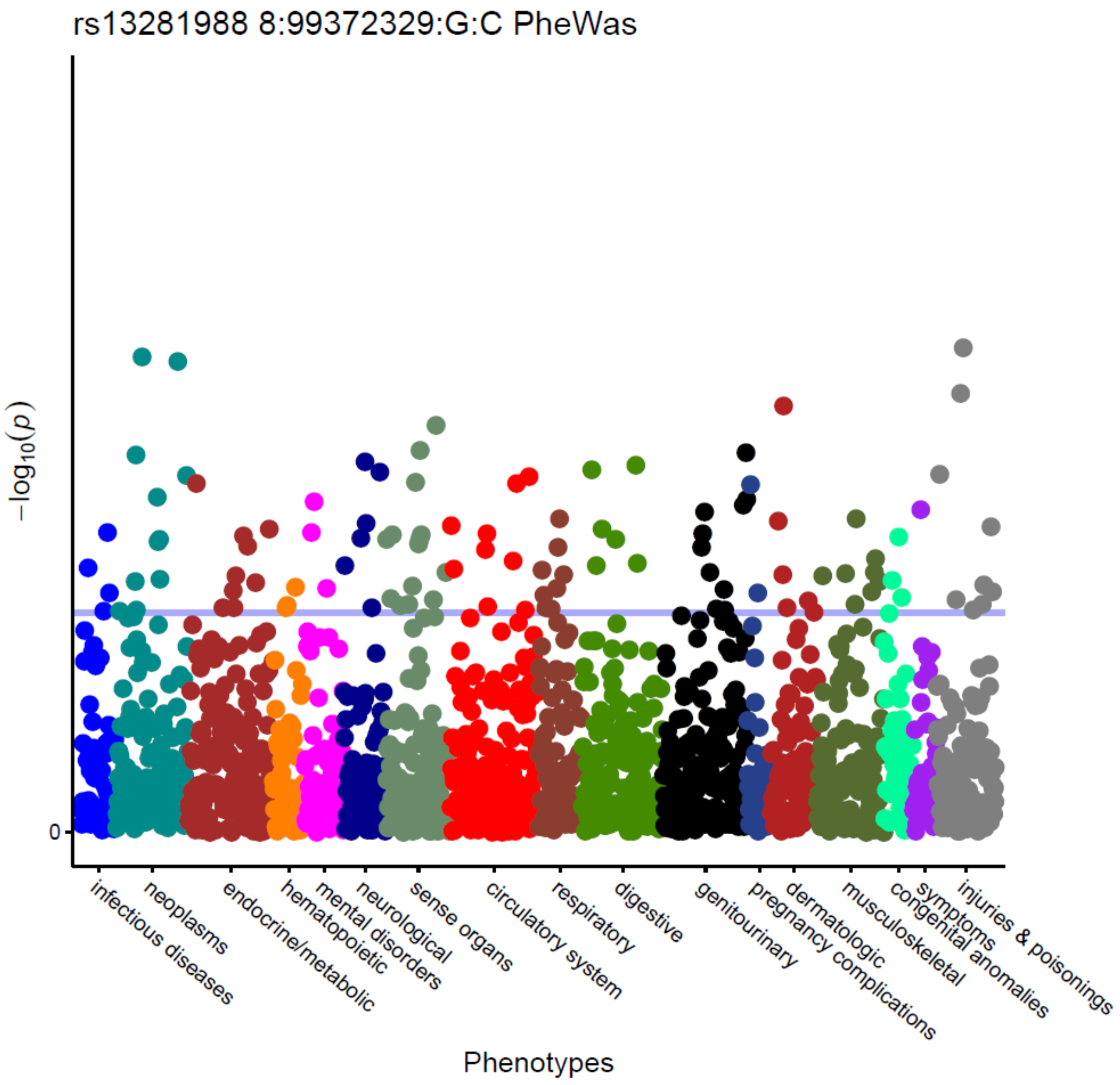

rs13281988 8:99372329:G:C PheWas
−log10(p)
0
infectious diseases
neoplasms
endocrine/metabolic
hematopoietic
mental disorders
neurological
sense organs
circulatory system
respiratory
digestive
genitourinary
pregnancy complications
dermatologic
musculoskeletal
congenital anomalies
symptoms
injuries & poisonings
Phenotypes

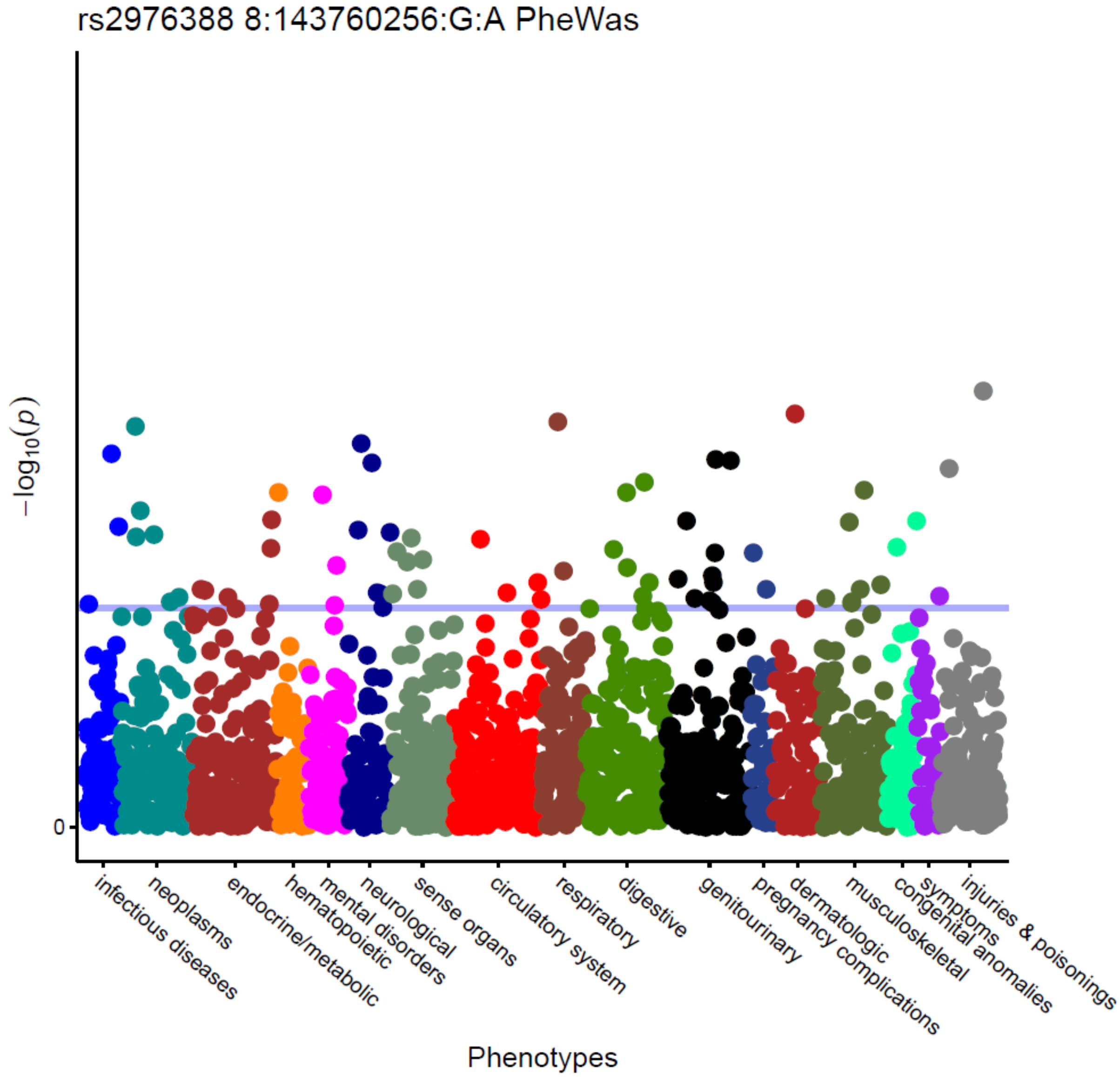

rs2976388 8:143760256:G:A PheWas
$-\log_{10}(p)$
0
infectious diseases
neoplasms
endocrine/metabolic
hematopoietic
mental disorders
neurological
sense organs
circulatory system
respiratory
digestive
genitourinary
pregnancy complications
dermatologic
musculoskeletal
congenital anomalies
symptoms
injuries & poisonings
Phenotypes

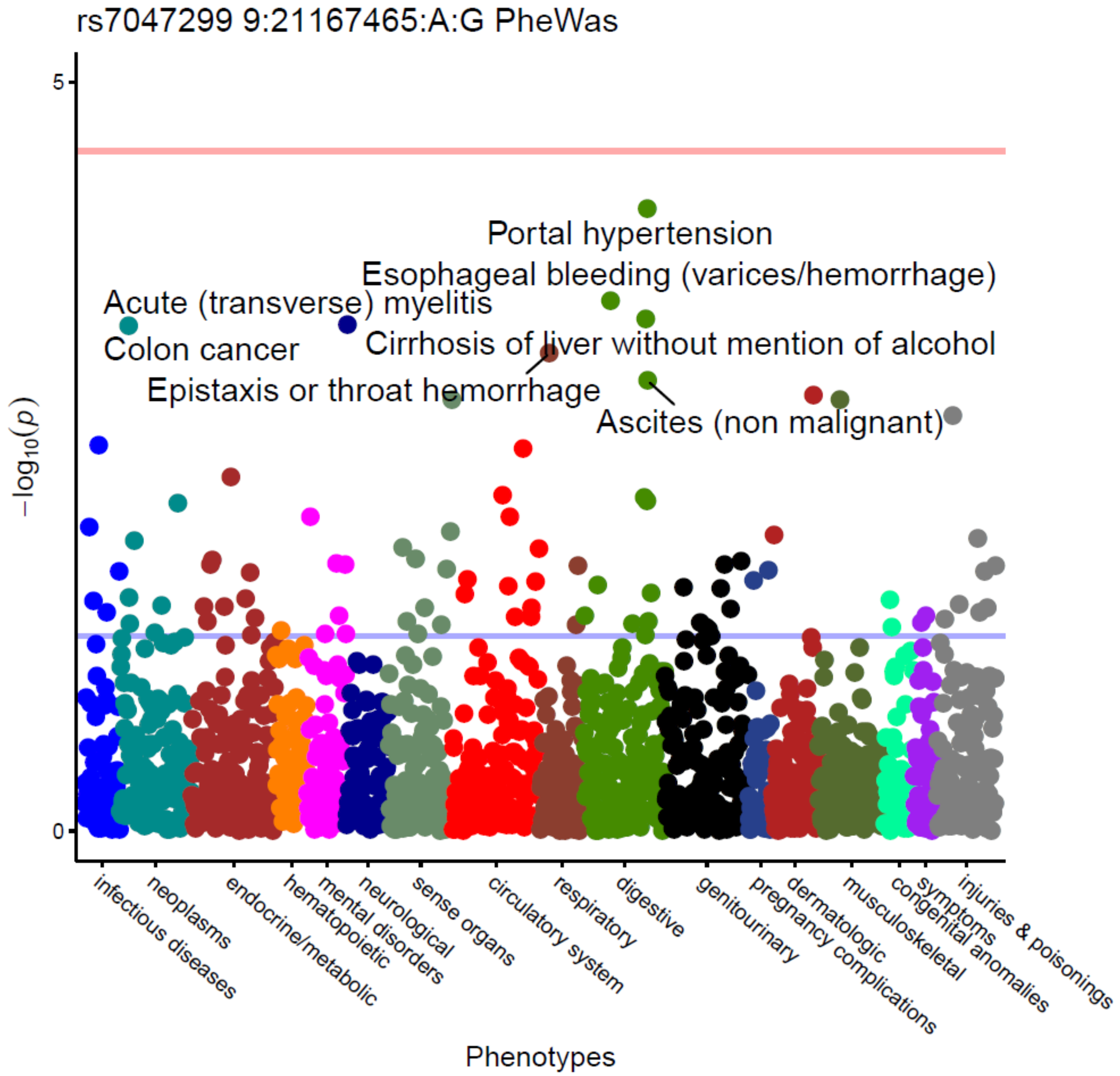
rs7047299 9:21167465:A:G PheWas
5
0
$-\log_{10}(p)$
Portal hypertension
Esophageal bleeding (varices/hemorrhage)
Acute (transverse) myelitis
Colon cancer
Cirrhosis of liver without mention of alcohol
Epistaxis or throat hemorrhage
Ascites (non malignant)
infectious diseases
neoplasms
endocrine/metabolic
hematopoietic
mental disorders
neurological
sense organs
circulatory system
respiratory
digestive
genitourinary
pregnancy complications
dermatologic
musculoskeletal
congenital anomalies
symptoms
injuries & poisonings
Phenotypes

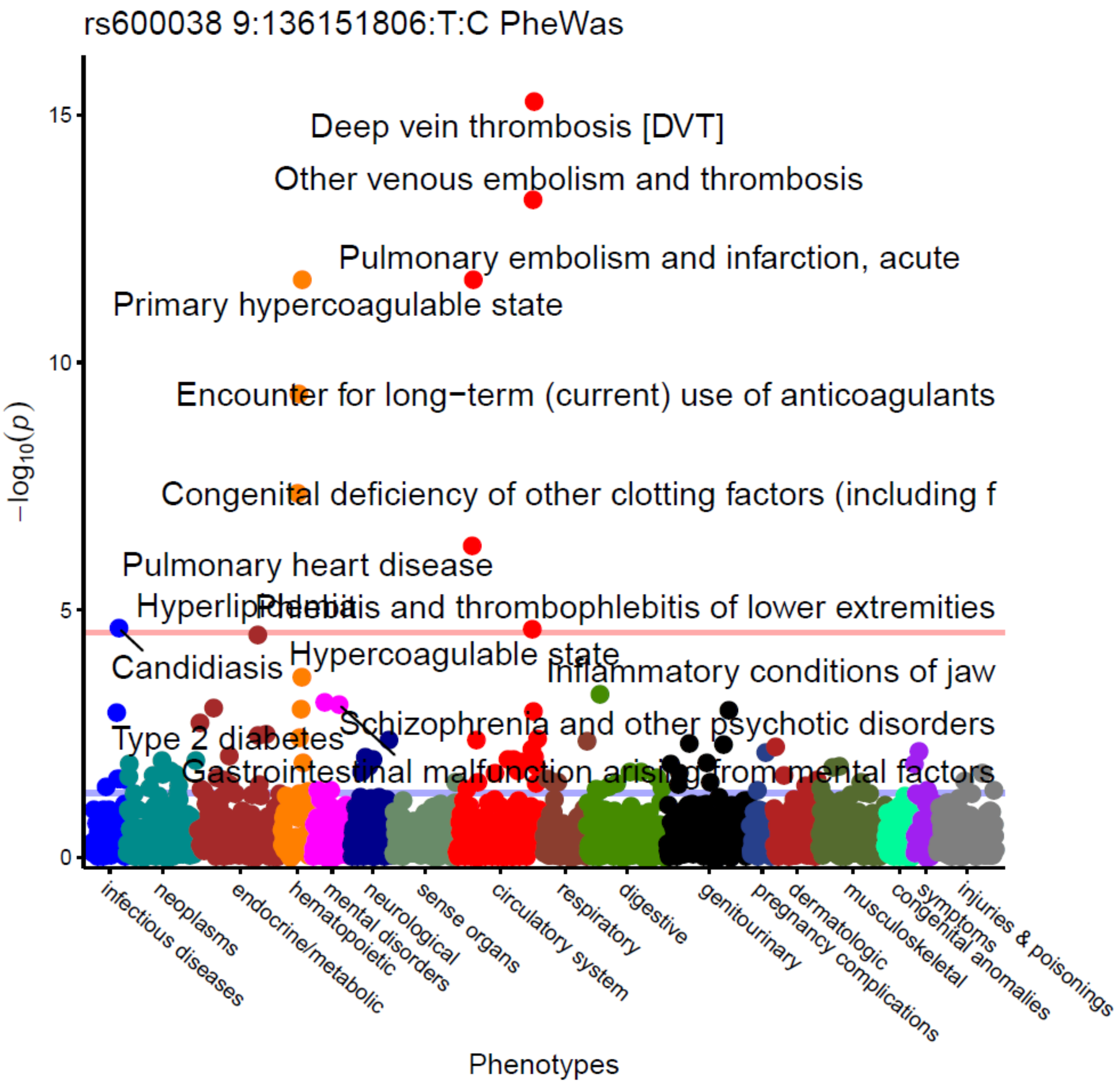

rs600038 9:136151806:T:C PheWas
Deep vein thrombosis [DVT]
Other venous embolism and thrombosis
Pulmonary embolism and infarction, acute
Primary hypercoagulable state
Encounter for long−term (current) use of anticoagulants
Congenital deficiency of other clotting factors (including f
Pulmonary heart disease
Hyperlipidemia
Phlebitis and thrombophlebitis of lower extremities
Candidiasis
Hypercoagulable state
Inflammatory conditions of jaw
Schizophrenia and other psychotic disorders
Type 2 diabetes
Gastrointestinal malfunction arising from mental factors
$-\log_{10}(p)$
15
10
5
0
infectious diseases
neoplasms
endocrine/metabolic
hematopoietic
mental disorders
neurological
sense organs
circulatory system
respiratory
digestive
genitourinary
pregnancy complications
dermatologic
musculoskeletal
congenital anomalies
symptoms
injuries & poisonings
Phenotypes

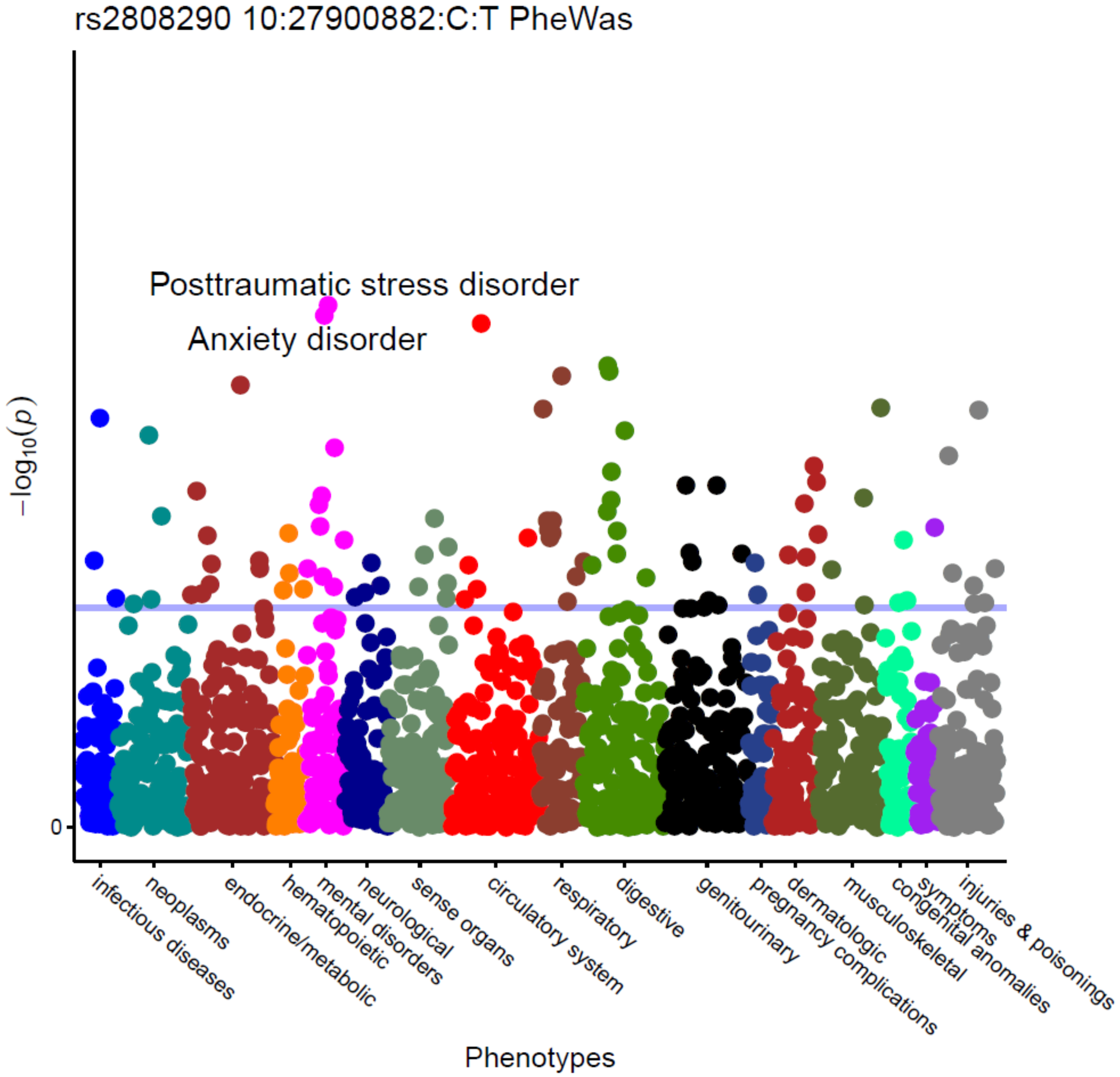
rs2808290 10:27900882:C:T PheWas
Posttraumatic stress disorder
Anxiety disorder
$-\log_{10}(p)$
0
infectious diseases
neoplasms
endocrine/metabolic
hematopoietic
mental disorders
neurological
sense organs
circulatory system
respiratory
digestive
genitourinary
pregnancy complications
dermatologic
musculoskeletal
congenital anomalies
symptoms
injuries & poisonings
Phenotypes

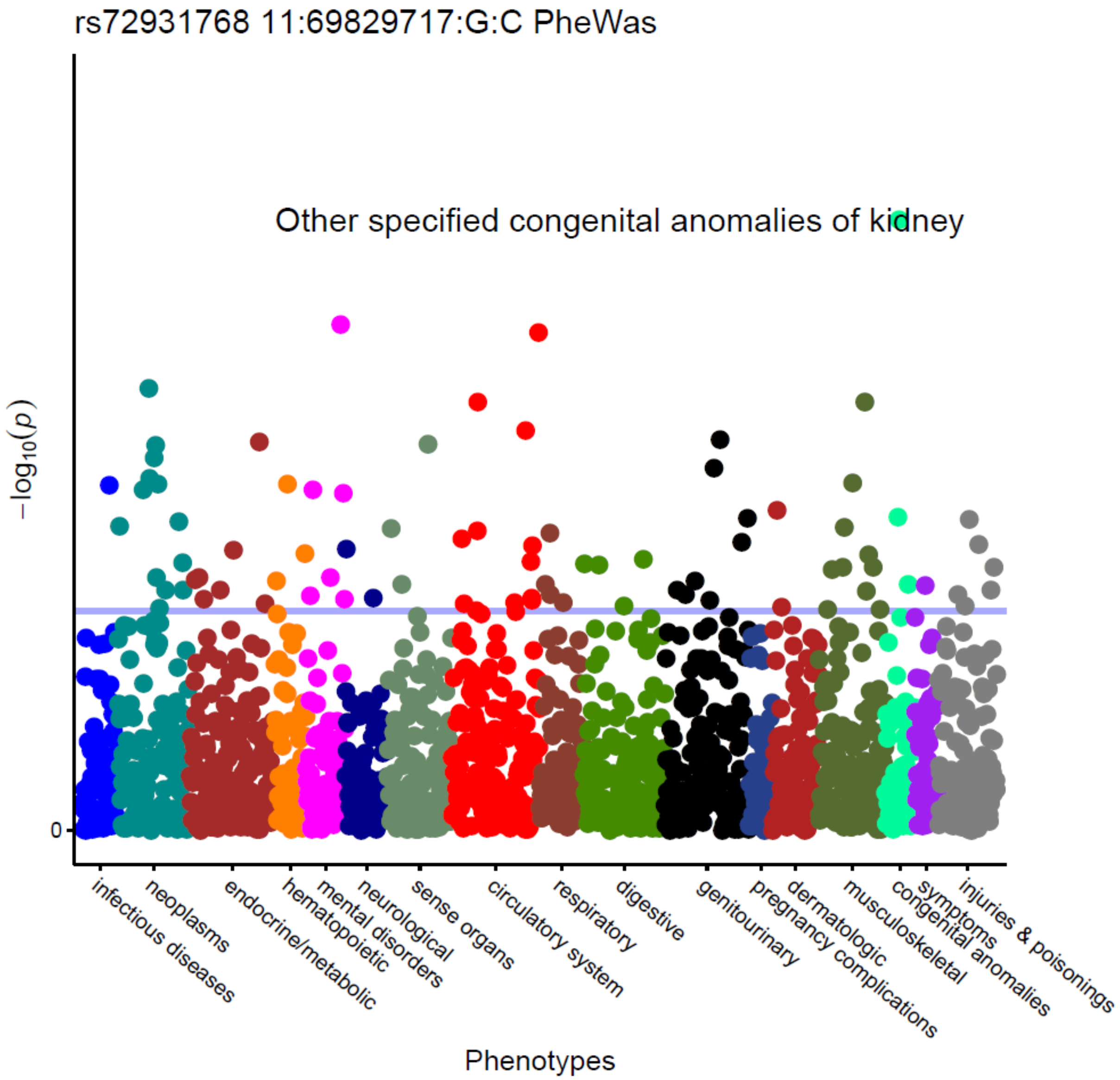
rs72931768 11:69829717:G:C PheWas
Other specified congenital anomalies of kidney
$-\log_{10}(p)$
0
infectious diseases
neoplasms
endocrine/metabolic
hematopoietic
mental disorders
neurological
sense organs
circulatory system
respiratory
digestive
genitourinary
pregnancy complications
dermatologic
musculoskeletal
congenital anomalies
symptoms
injuries & poisonings
Phenotypes

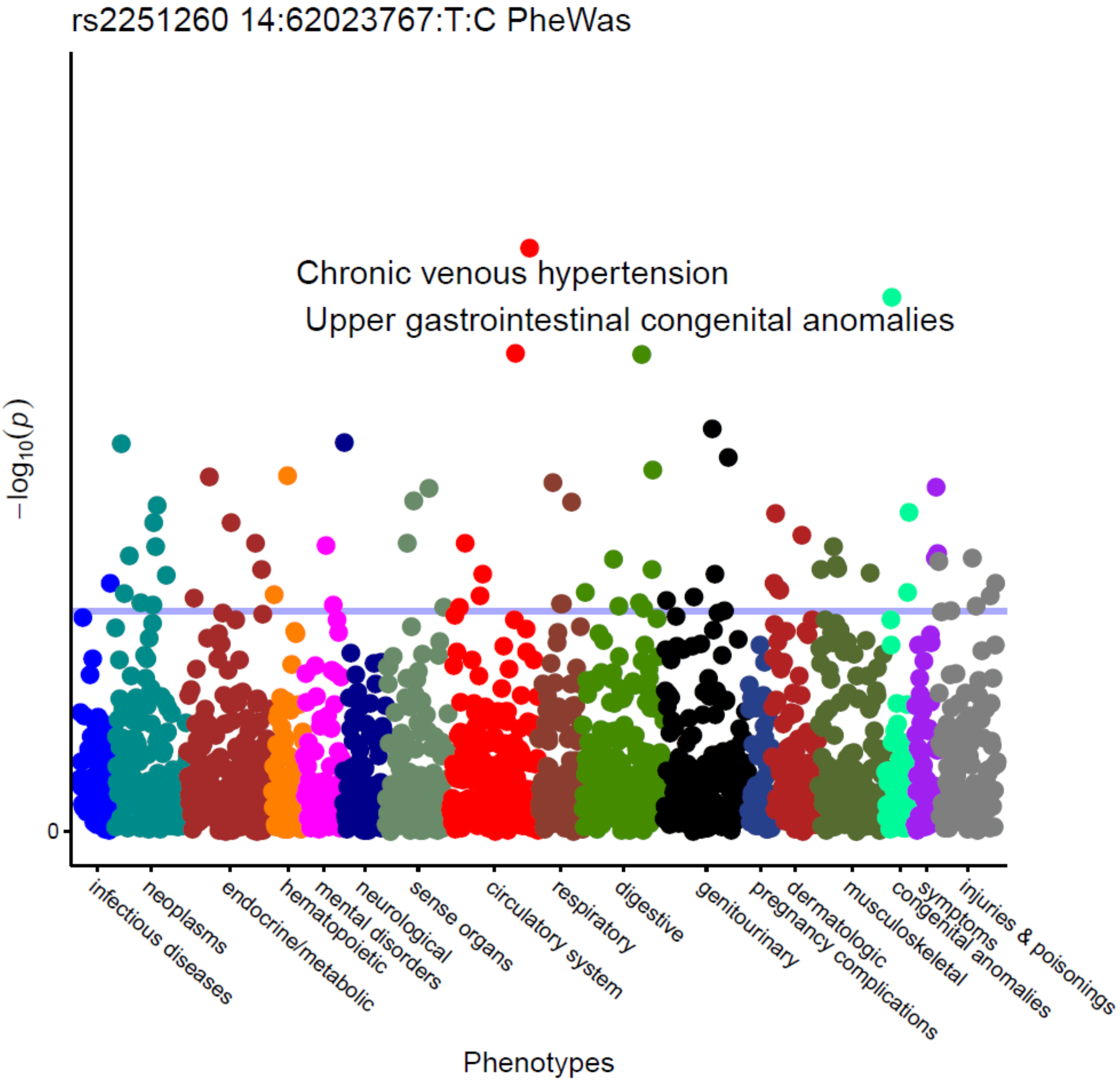

rs2251260 14:62023767:T:C PheWas
Chronic venous hypertension
Upper gastrointestinal congenital anomalies
−log10(p)
0
infectious diseases
neoplasms
endocrine/metabolic
hematopoietic
mental disorders
neurological
sense organs
circulatory system
respiratory
digestive
genitourinary
pregnancy complications
dermatologic
musculoskeletal
congenital anomalies
symptoms
injuries & poisonings
Phenotypes

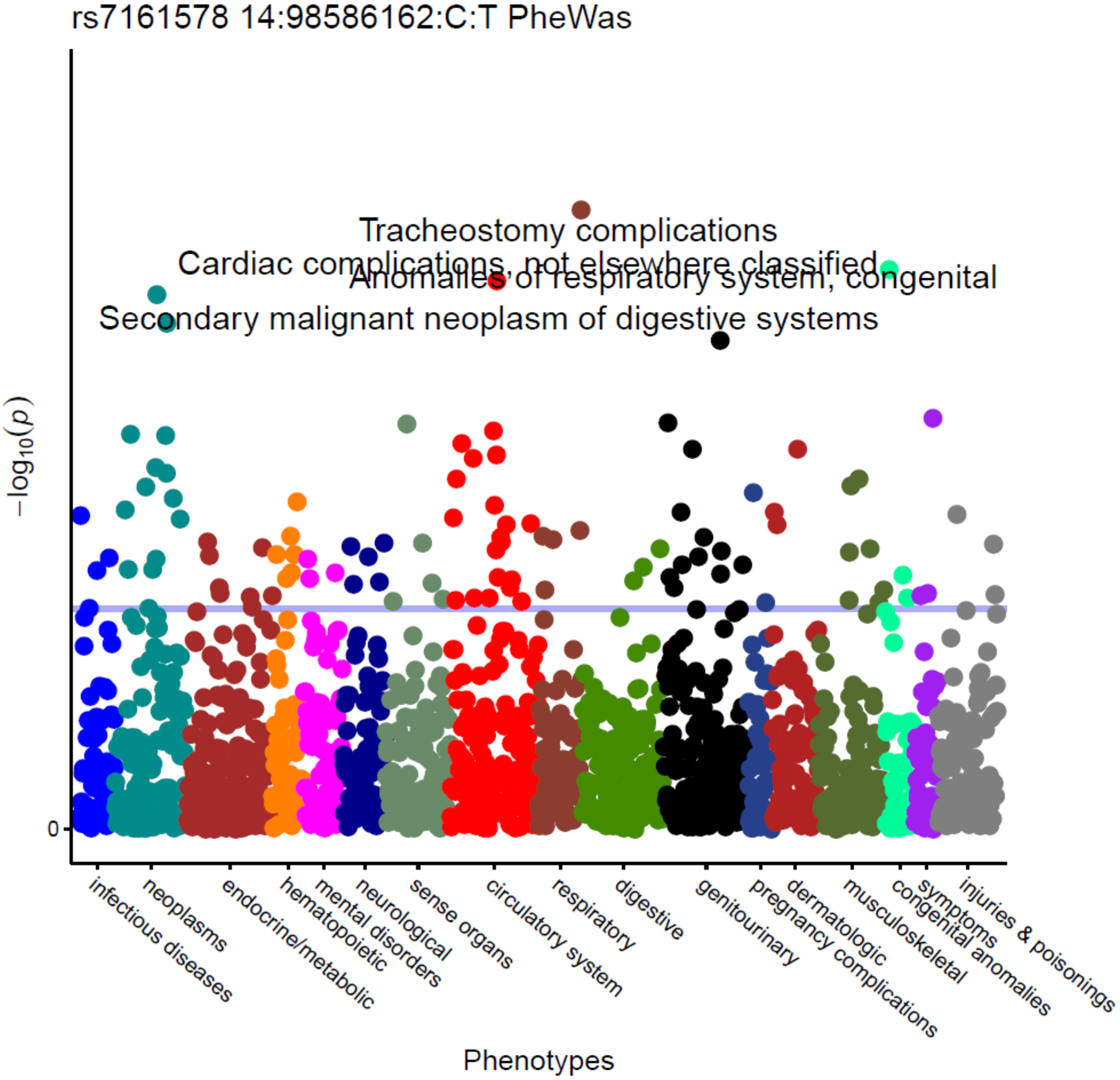
rs7161578 14:98586162:C:T PheWas
Tracheostomy complications
Cardiac complications, not elsewhere classified
Anomalies of respiratory system, congenital
Secondary malignant neoplasm of digestive systems
−log10(p)
0
infectious diseases
neoplasms
endocrine/metabolic
hematopoietic
mental disorders
neurological
sense organs
circulatory system
respiratory
digestive
genitourinary
pregnancy complications
dermatologic
musculoskeletal
congenital anomalies
symptoms
injuries & poisonings
Phenotypes

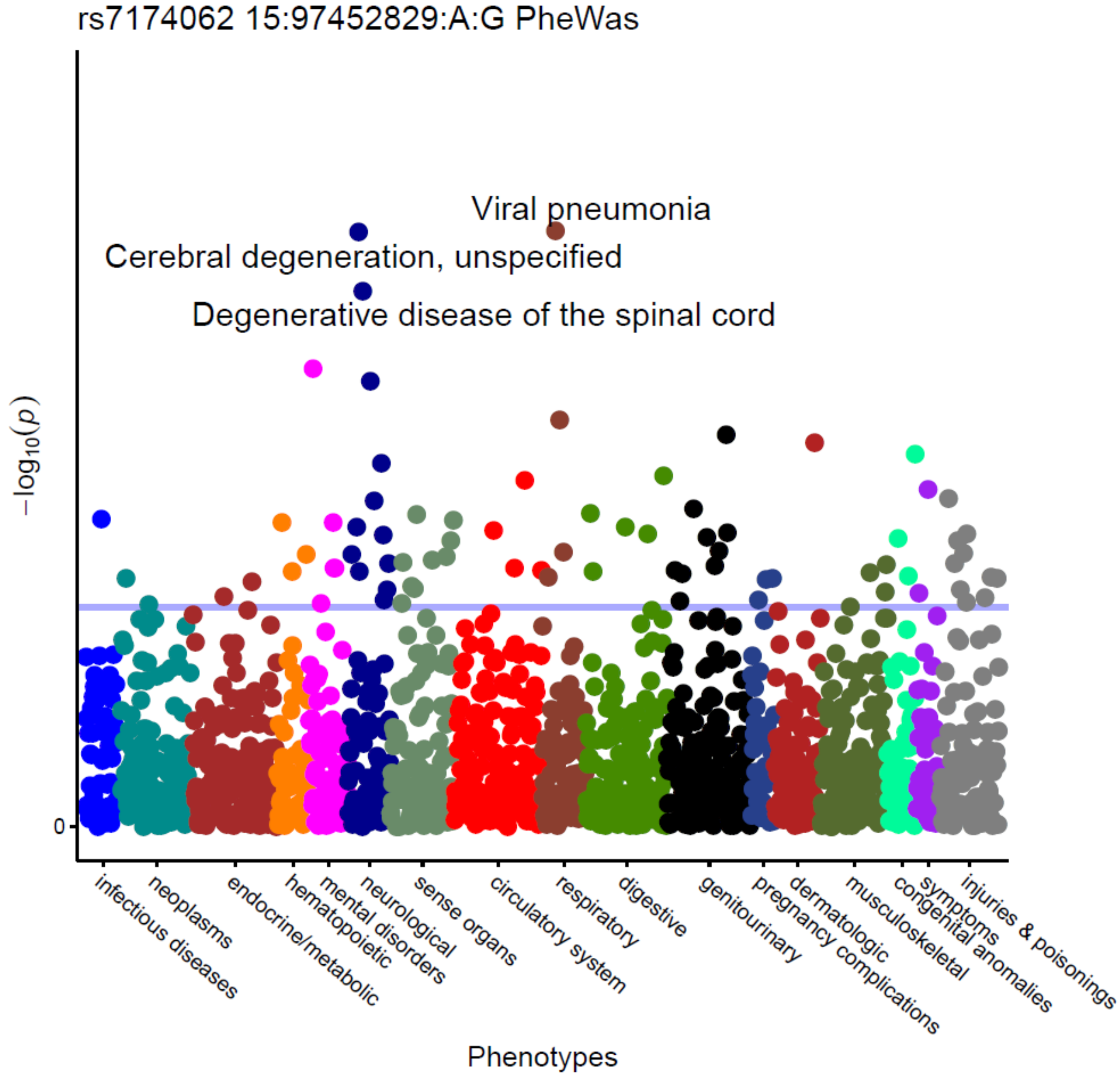

rs7174062 15:97452829:A:G PheWas
Viral pneumonia
Cerebral degeneration, unspecified
Degenerative disease of the spinal cord
−log10(p)
0
infectious diseases
neoplasms
endocrine/metabolic
hematopoietic
mental disorders
neurological
sense organs
circulatory system
respiratory
digestive
genitourinary
pregnancy complications
dermatologic
musculoskeletal
congenital anomalies
symptoms
injuries & poisonings
Phenotypes

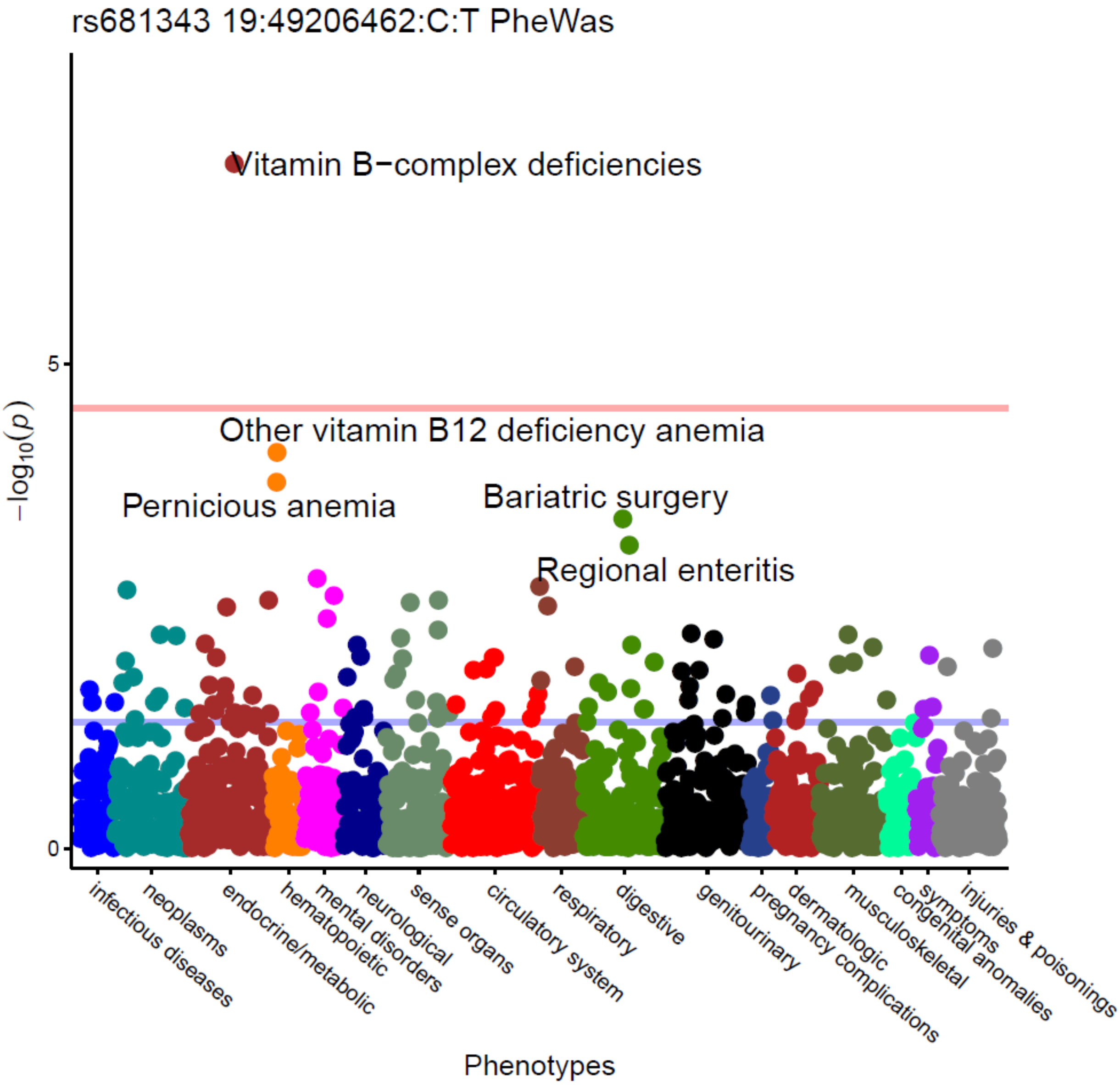
rs681343 19:49206462:C:T PheWas
Vitamin B−complex deficiencies
Other vitamin B12 deficiency anemia
Pernicious anemia
Bariatric surgery
Regional enteritis
−log10(p)
5
0
infectious diseases
neoplasms
endocrine/metabolic
hematopoietic
mental disorders
neurological
sense organs
circulatory system
respiratory
digestive
genitourinary
pregnancy complications
dermatologic
musculoskeletal
congenital anomalies
symptoms
injuries & poisonings
Phenotypes

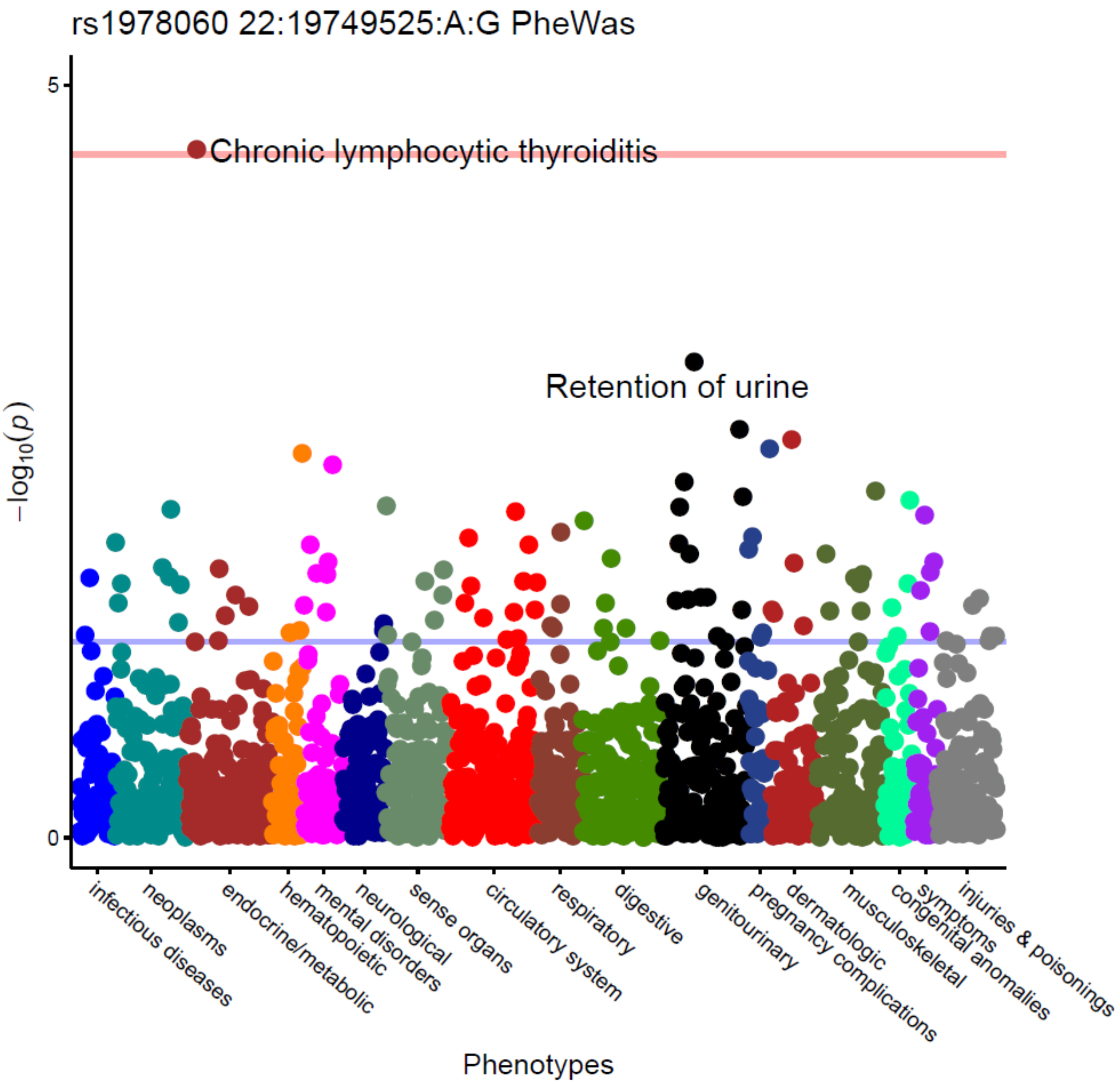

rs1978060 22:19749525:A:G PheWas
5
0
−log10(p)
Chronic lymphocytic thyroiditis
Retention of urine
infectious diseases
neoplasms
endocrine/metabolic
hematopoietic
mental disorders
neurological
sense organs
circulatory system
respiratory
digestive
genitourinary
pregnancy complications
dermatologic
musculoskeletal
congenital anomalies
symptoms
injuries & poisonings
Phenotypes

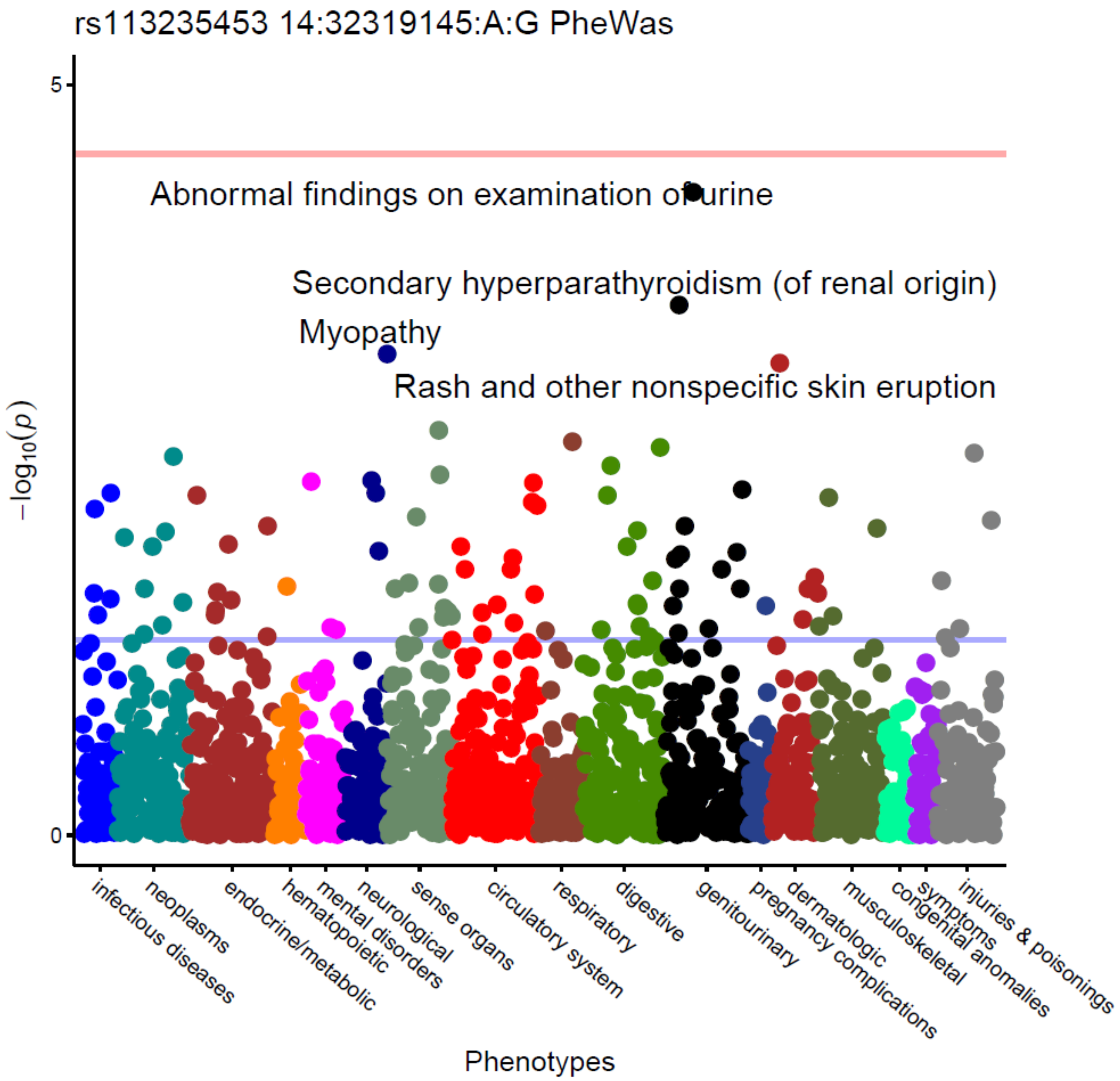

rs113235453 14:32319145:A:G PheWas
5
0
−log10(p)
Abnormal findings on examination of urine
Secondary hyperparathyroidism (of renal origin)
Myopathy
Rash and other nonspecific skin eruption
infectious diseases
neoplasms
endocrine/metabolic
hematopoietic
mental disorders
neurological
sense organs
circulatory system
respiratory
digestive
genitourinary
pregnancy complications
dermatologic
musculoskeletal
congenital anomalies
symptoms
injuries & poisonings
Phenotypes

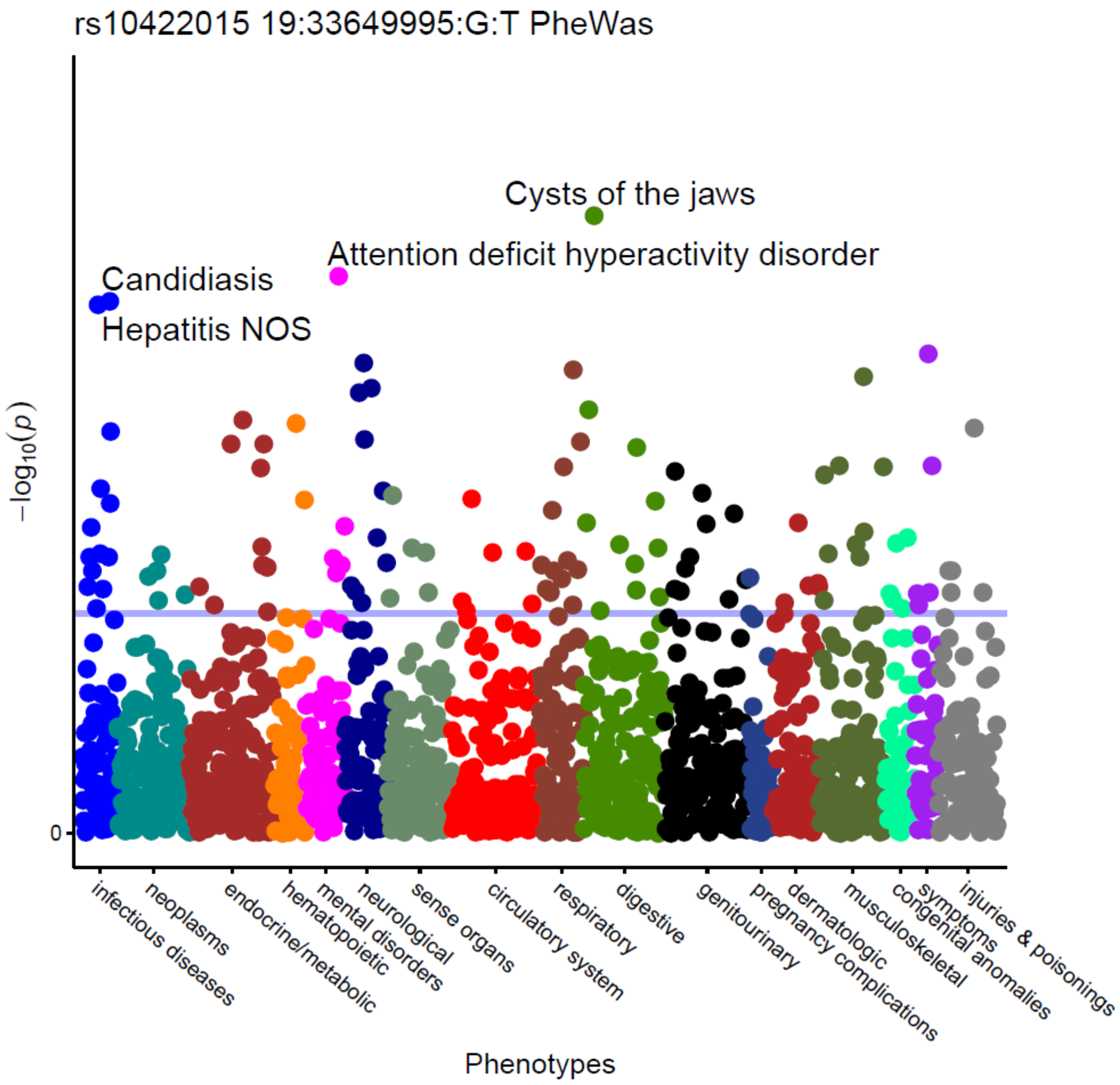
rs10422015 19:33649995:G:T PheWas
Cysts of the jaws
Attention deficit hyperactivity disorder
Candidiasis
Hepatitis NOS
−log10(p)
0
infectious diseases
neoplasms
endocrine/metabolic
hematopoietic
mental disorders
neurological
sense organs
circulatory system
respiratory
digestive
genitourinary
pregnancy complications
dermatologic
musculoskeletal
congenital anomalies
symptoms
injuries & poisonings
Phenotypes
