# Supplementary figures and images for: Genome-wide association analyses of common infections in a large practice-based biobank
Source: BMC Genomics. 2022 Sep 27;23:672. doi: 10.1186/s12864-022-08888-9 (PMC9512962; doi:10.1186/s12864-022-08888-9)

Additional file 4: Supplementary Figure 2. Manhattan plots and Q-Q plots of GWAS results

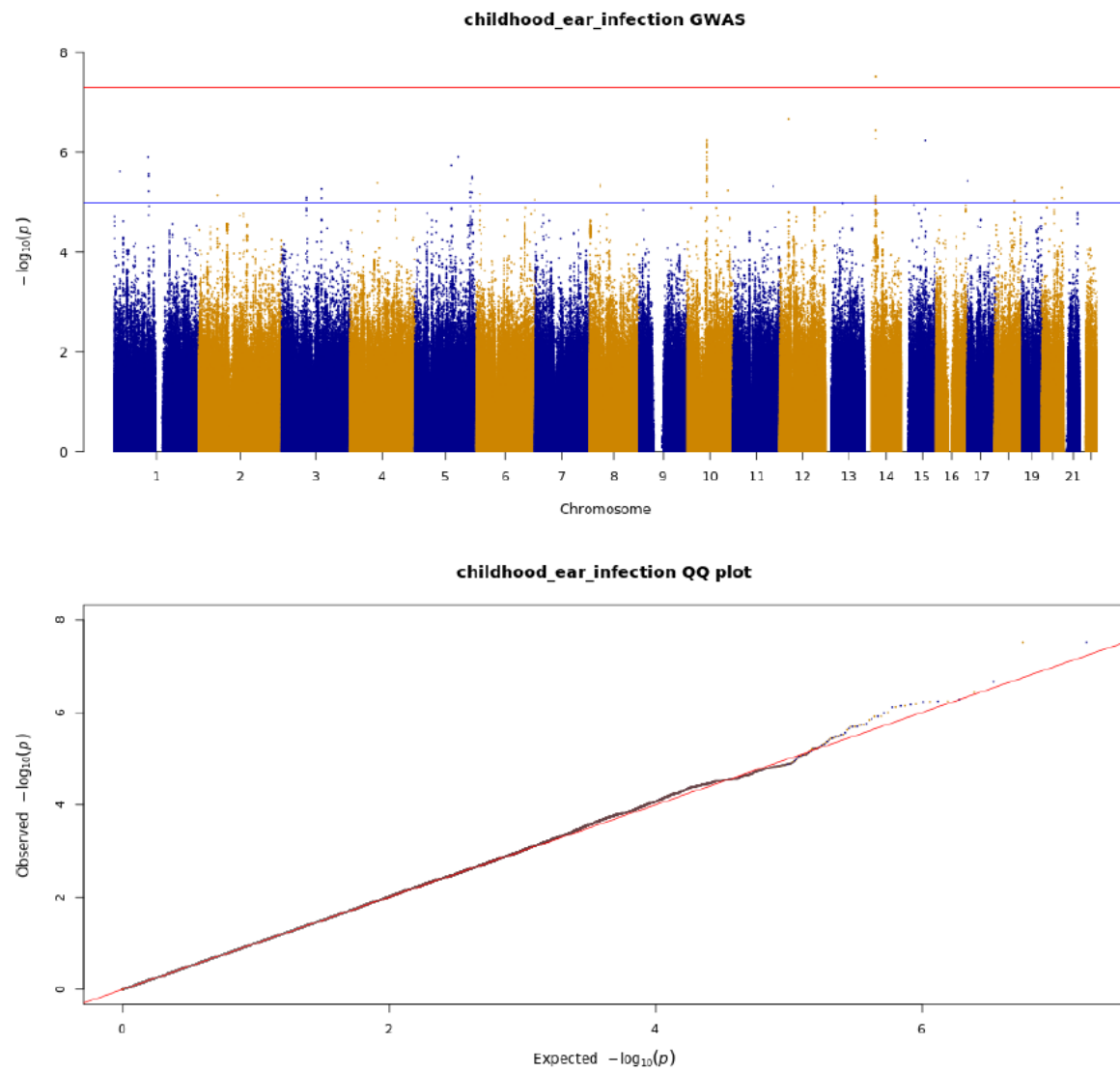

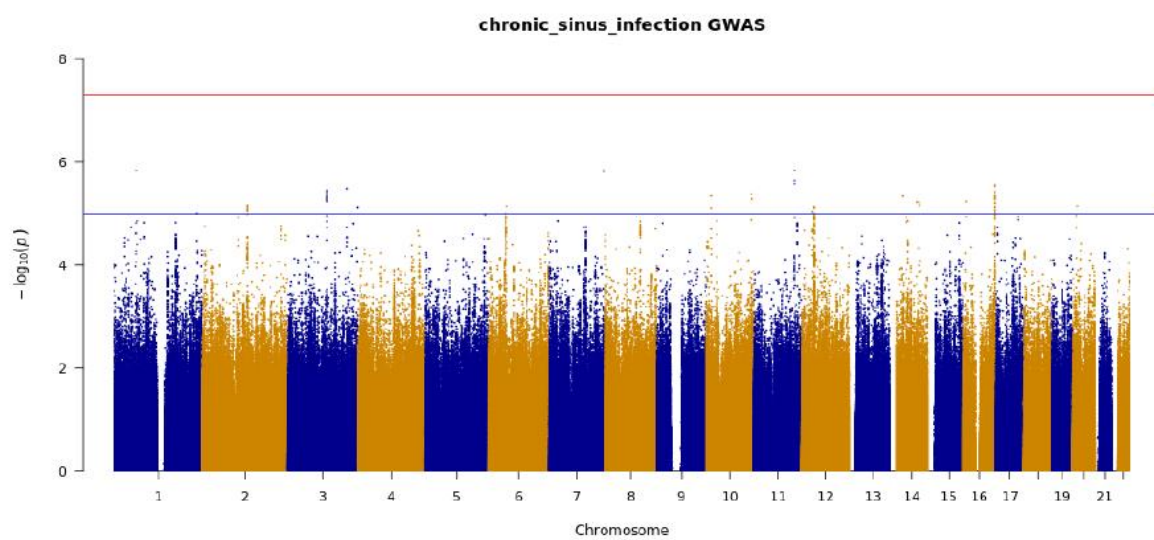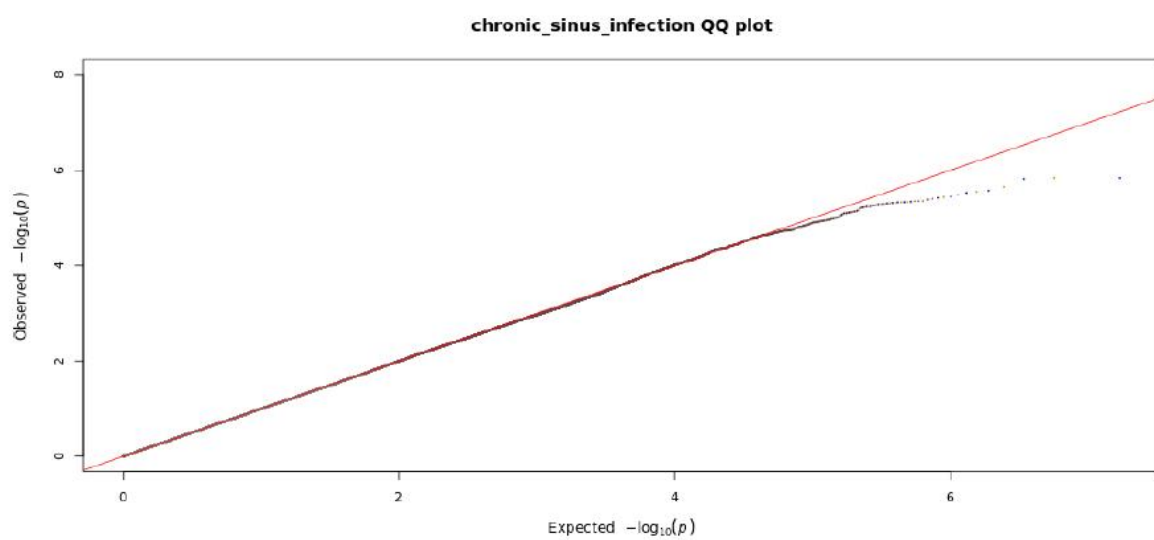

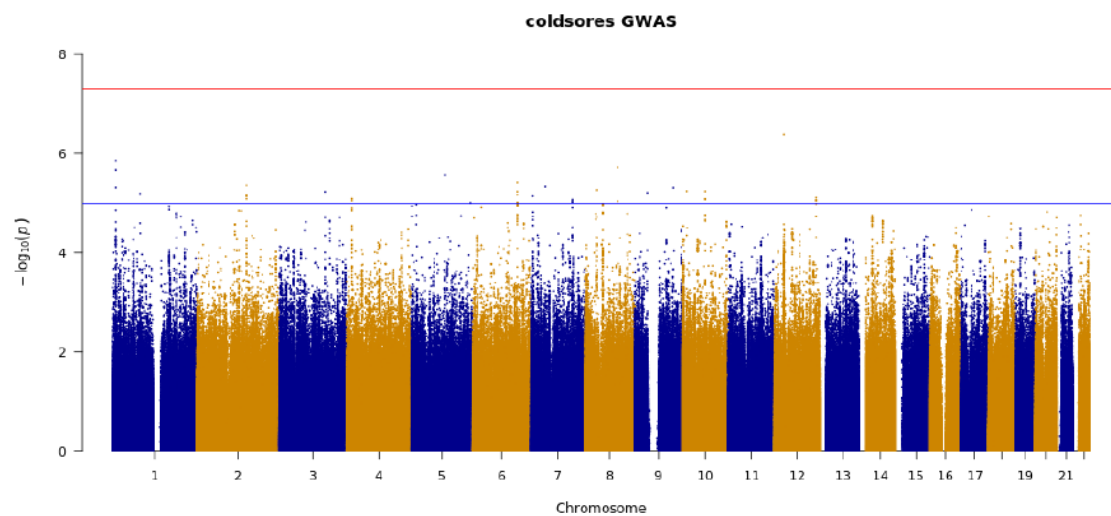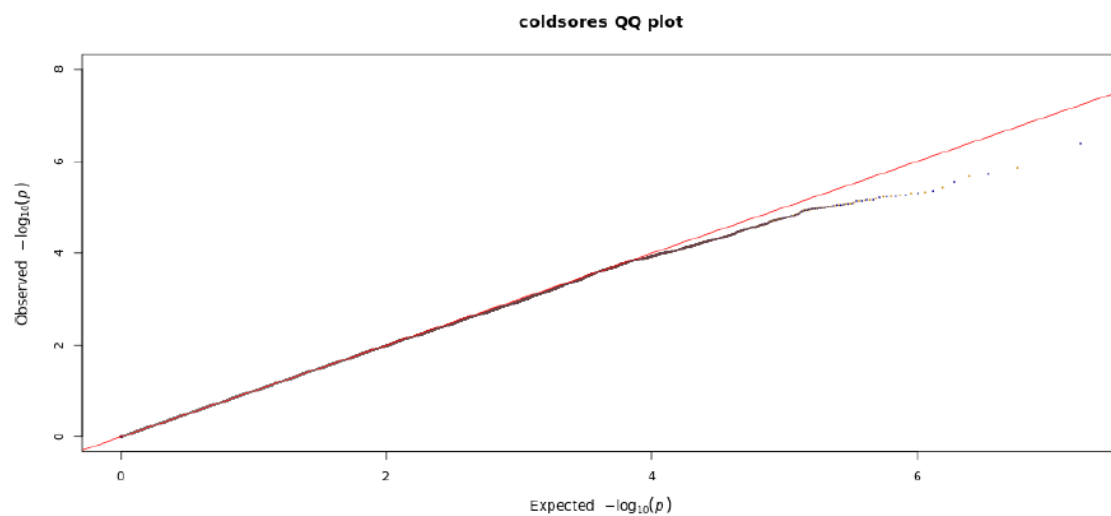

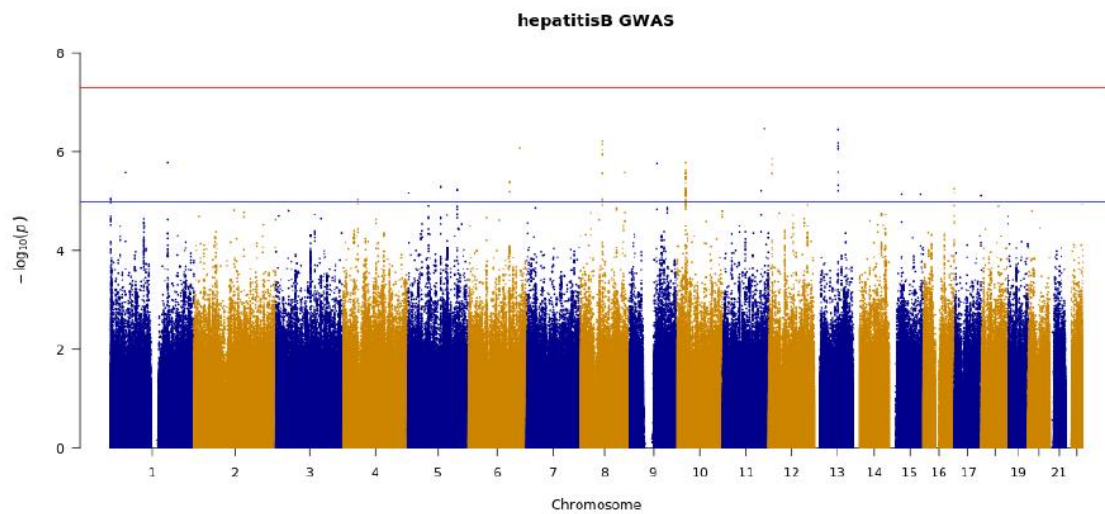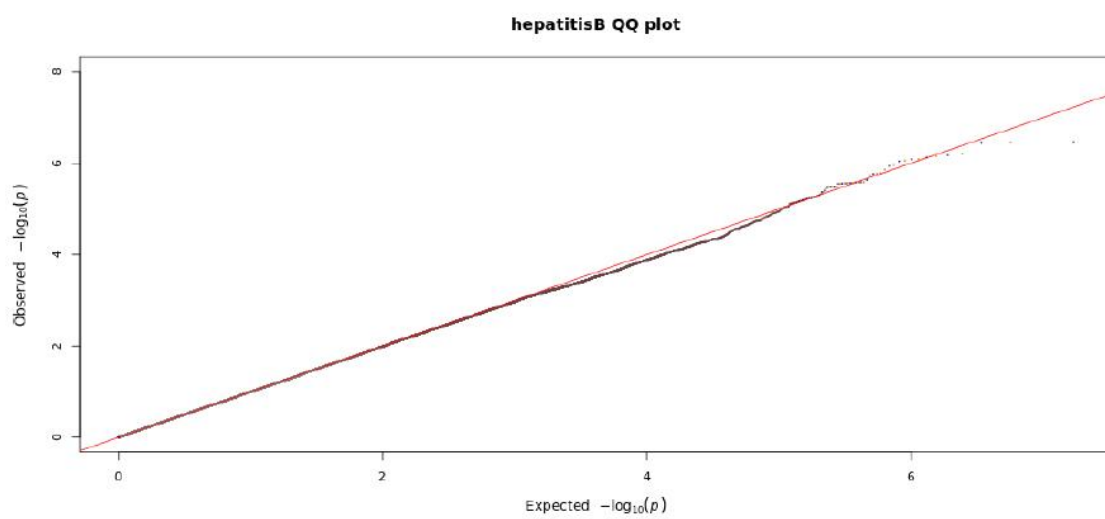

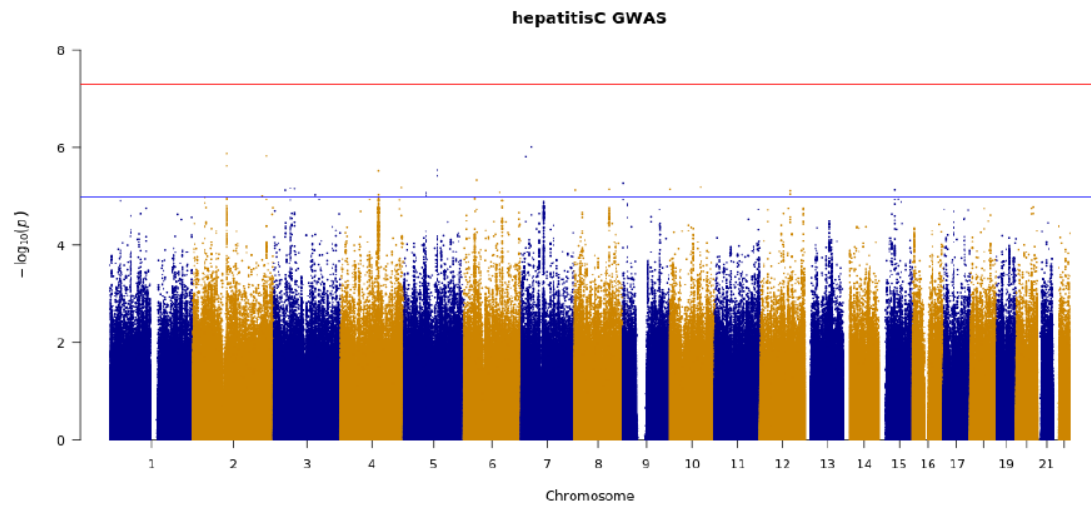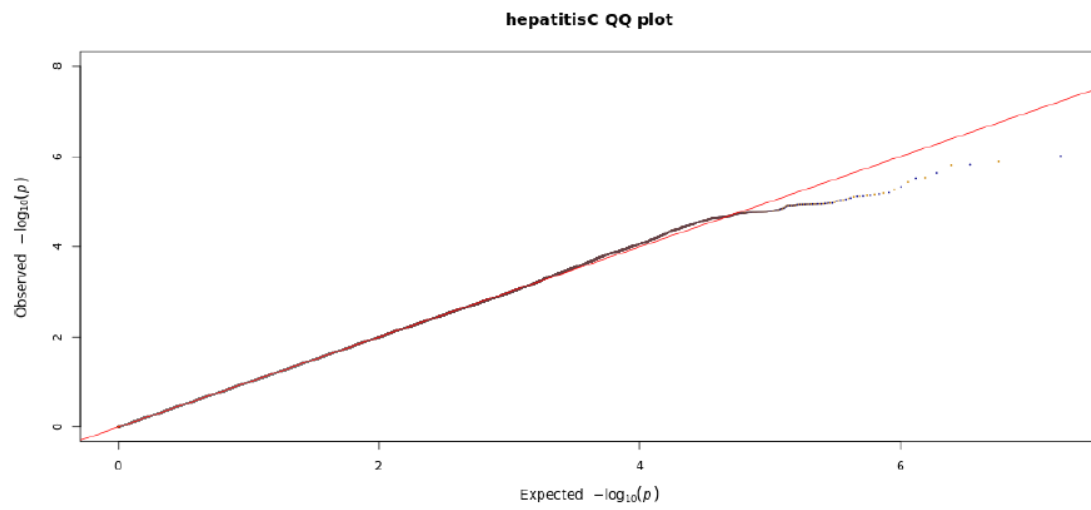

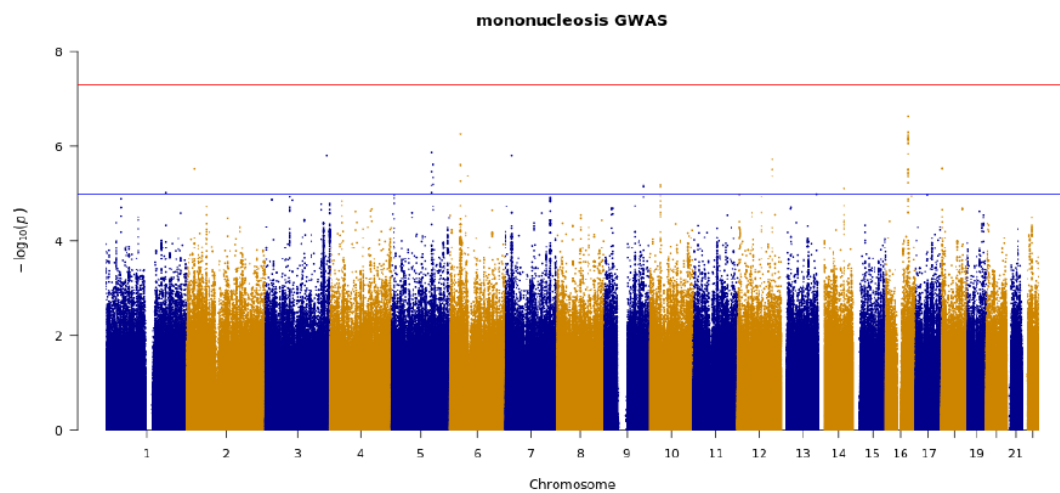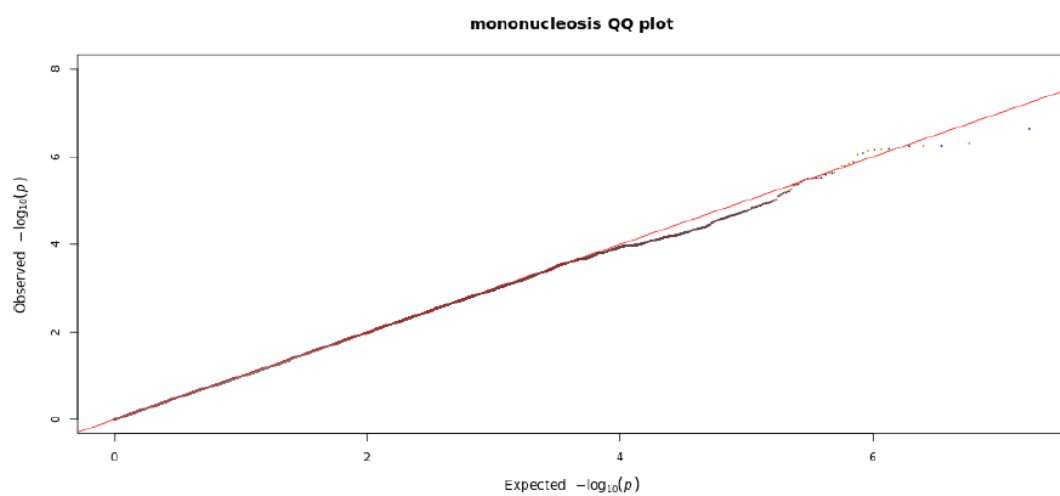

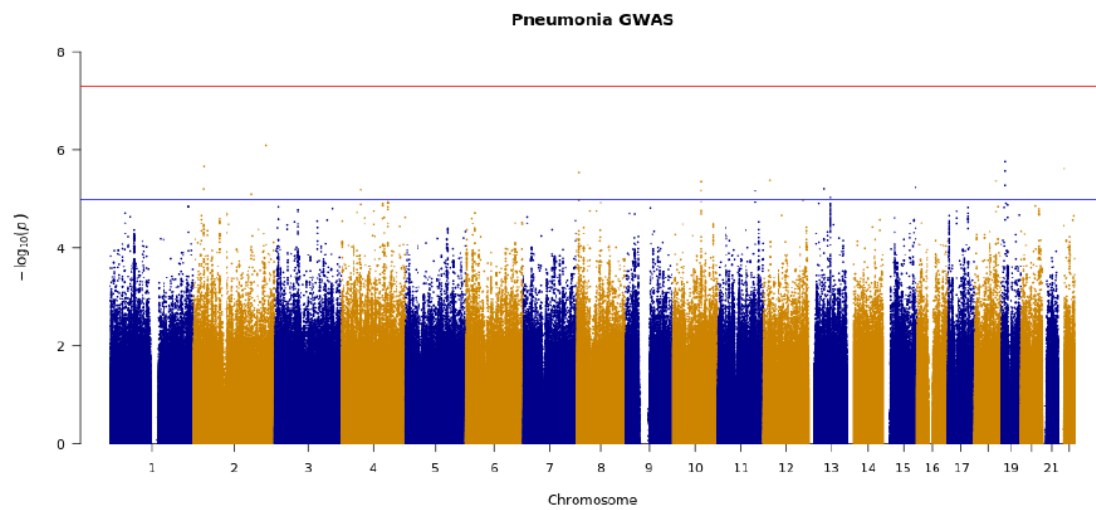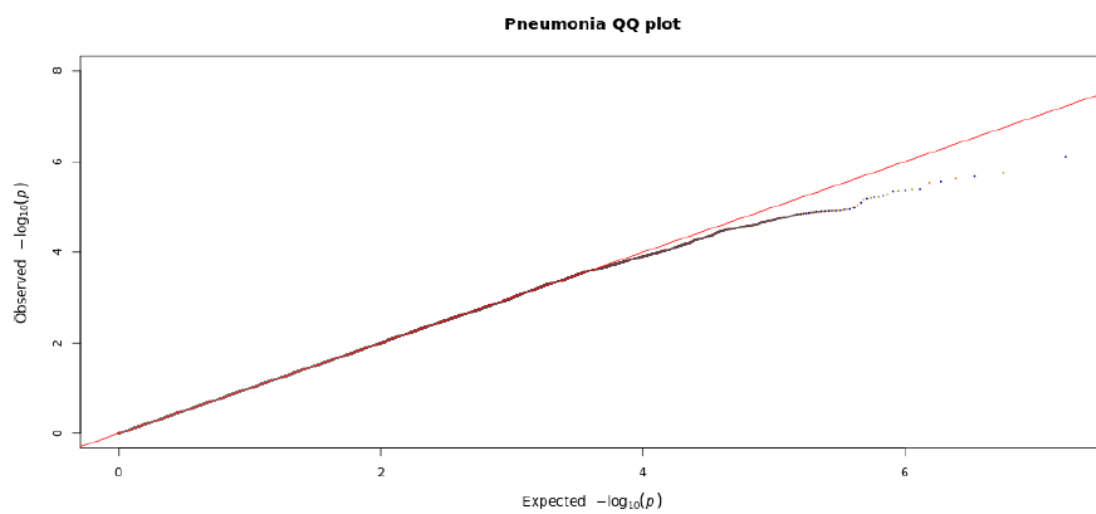

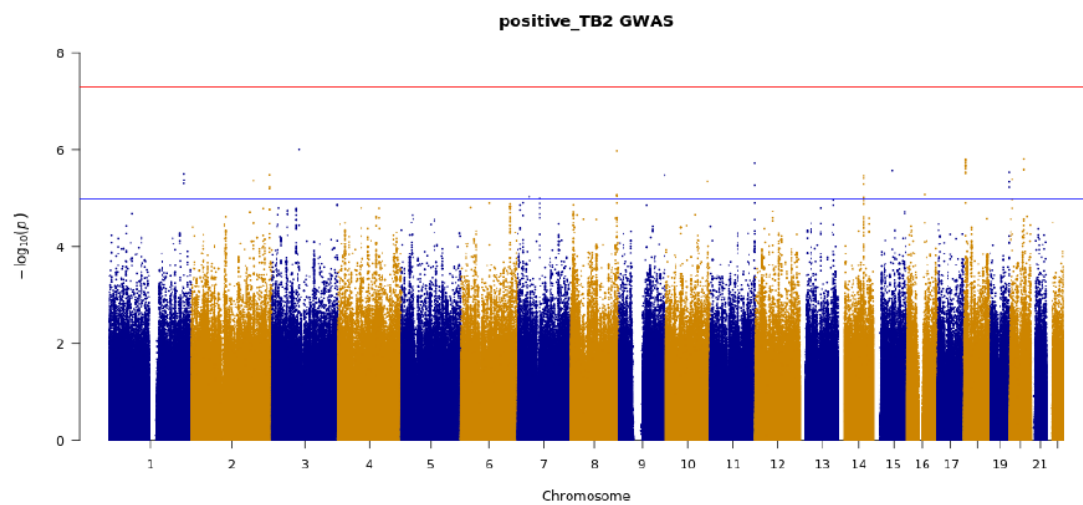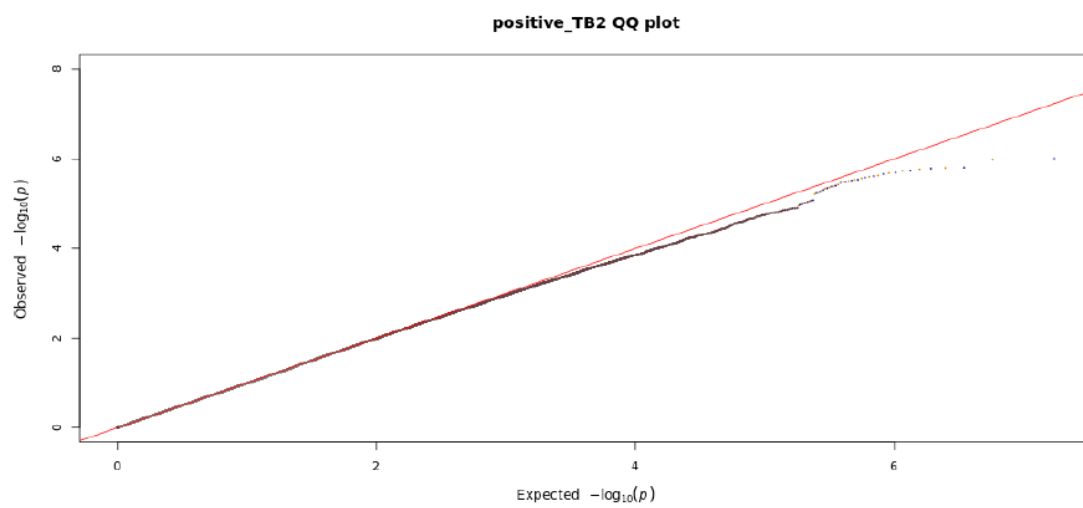

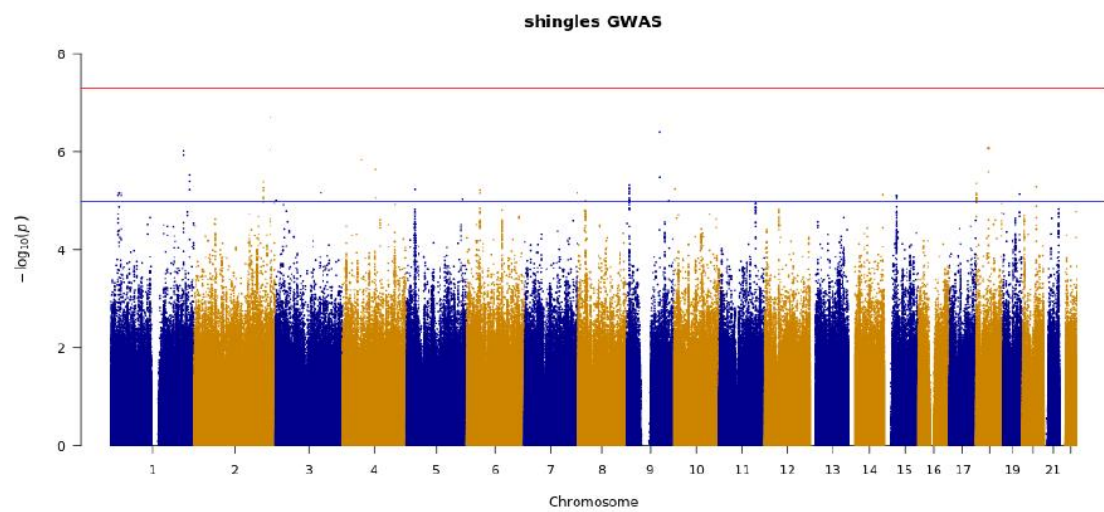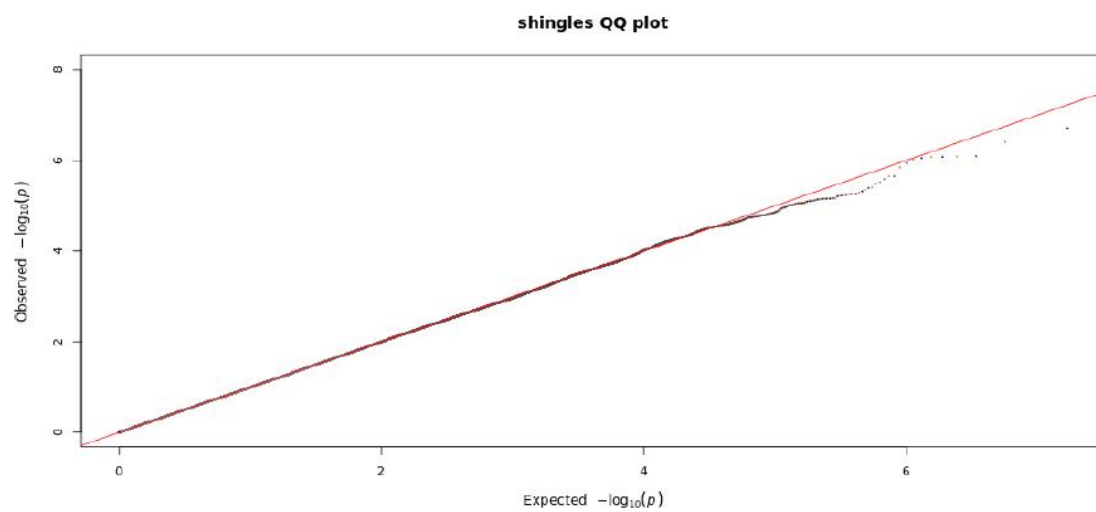

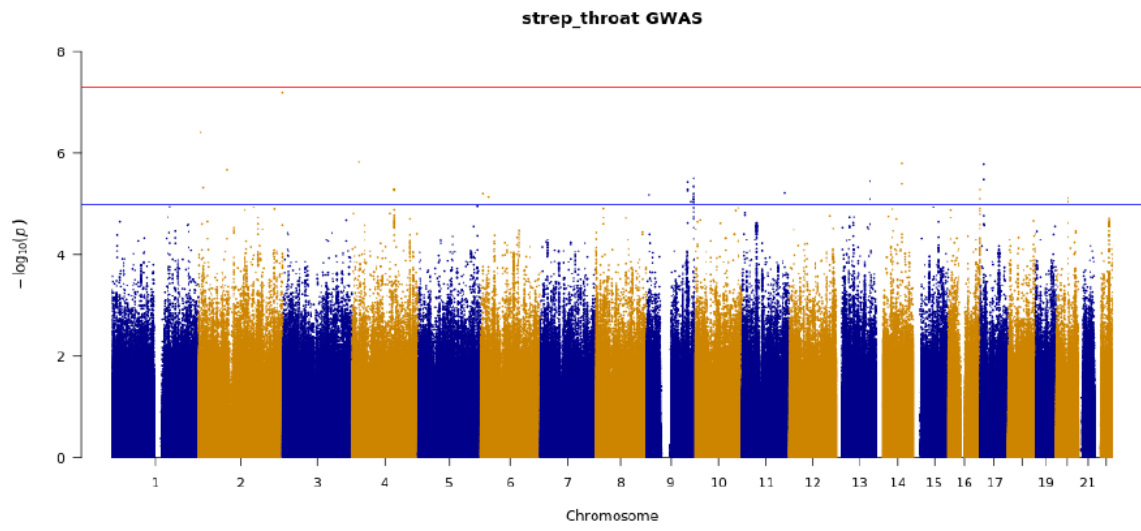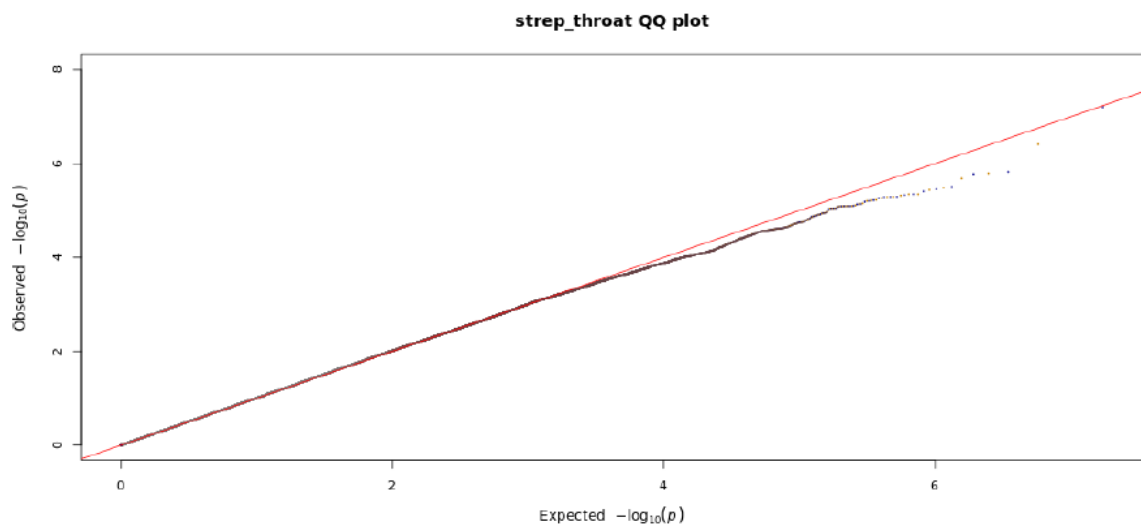

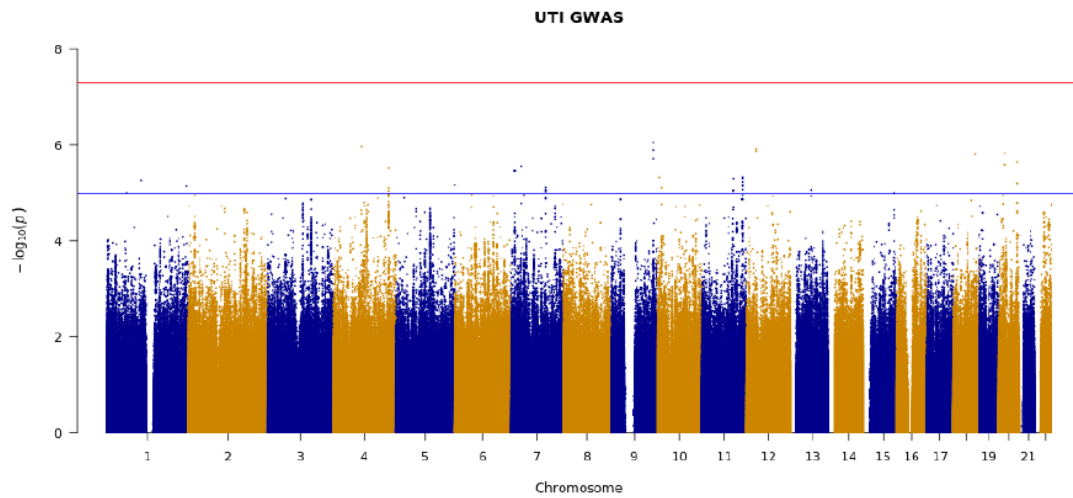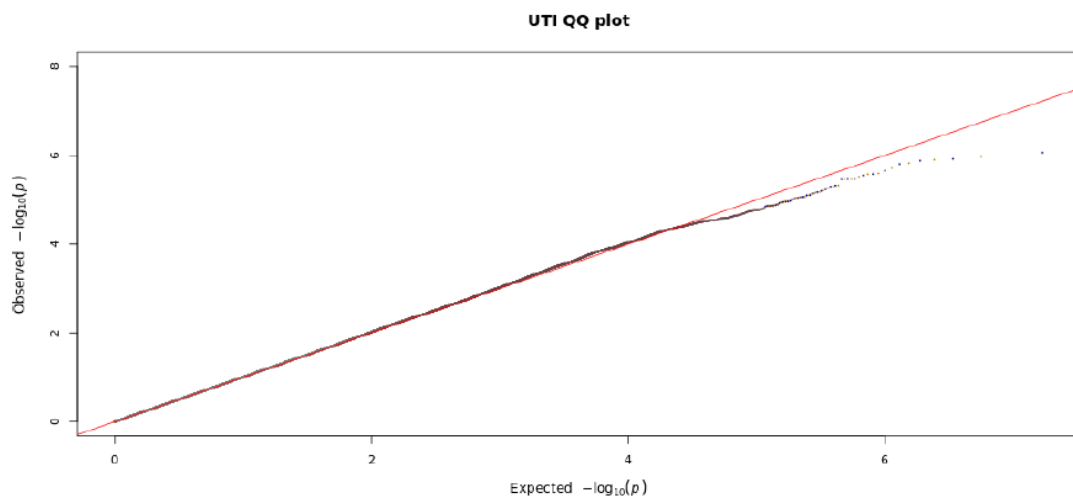

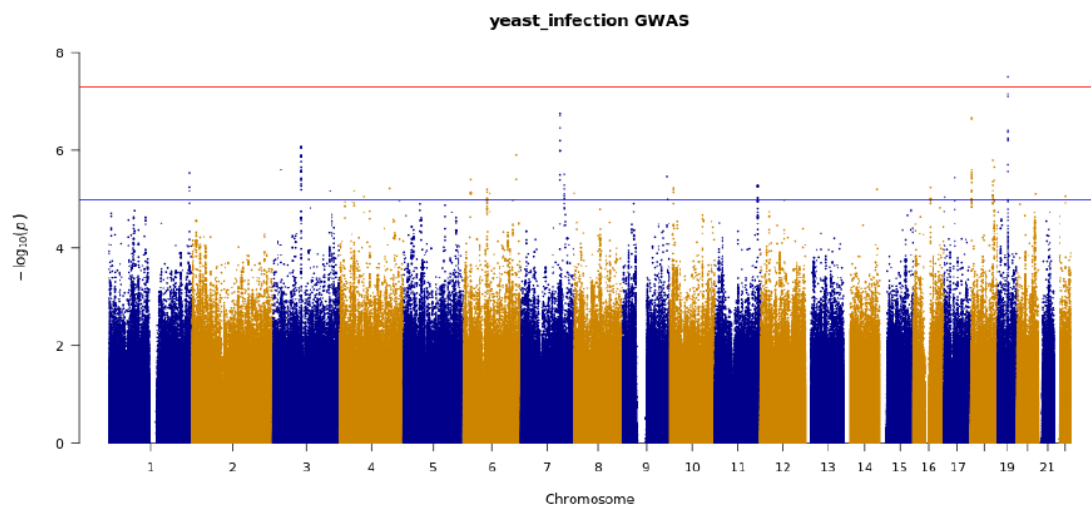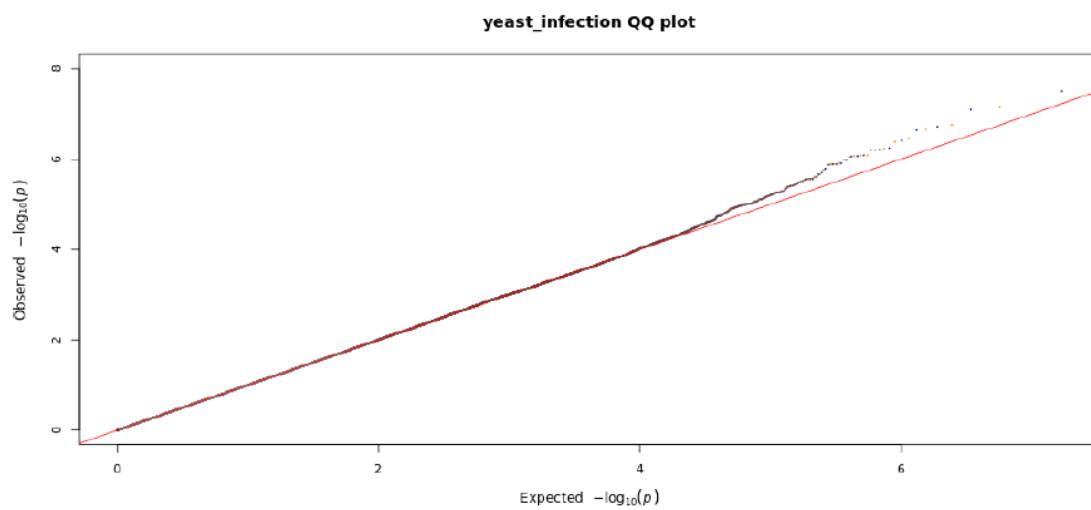

Supplement: Supplementary file 4 — Additional file 4: Supplementary Figure 2. Manhattan plots and Q-Q plots of GWAS results. [file 12864_2022_8888_MOESM4_ESM.pdf]
